# Supplementary material for: RagB stimulates the activity of the peptidoglycan polymerase RodA in Bacillus subtilis
Source: EMBO Rep. 2025 Aug 15;26(18):4587–606. doi: 10.1038/s44319-025-00547-w (PMC12457691; doi:10.1038/s44319-025-00547-w)
Supplement: Supplementary file 1 — Appendix [file 44319_2025_547_MOESM1_ESM.pdf]

APPENDIX

RagB stimulates the activity of the peptidoglycan polymerase RodA in *Bacillus subtilis*.

Pompeo *et al.*

Table of content

Appendix Figure S1……………………………………………………………………………………………………Page 2

Appendix Figure S2……………………………………………………………………………………………………Page 3

Appendix Figure S3……………………………………………………………………………………………………Page 4

Appendix Figure S4……………………………………………………………………………………………………Page 5

Appendix Figure S5……………………………………………………………………………………………………Page 6

Appendix Figure S6……………………………………………………………………………………………………Page 7

Appendix Figure S7……………………………………………………………………………………………………Pages 8-9

Appendix Figure S8……………………………………………………………………………………………………Pages 10-11

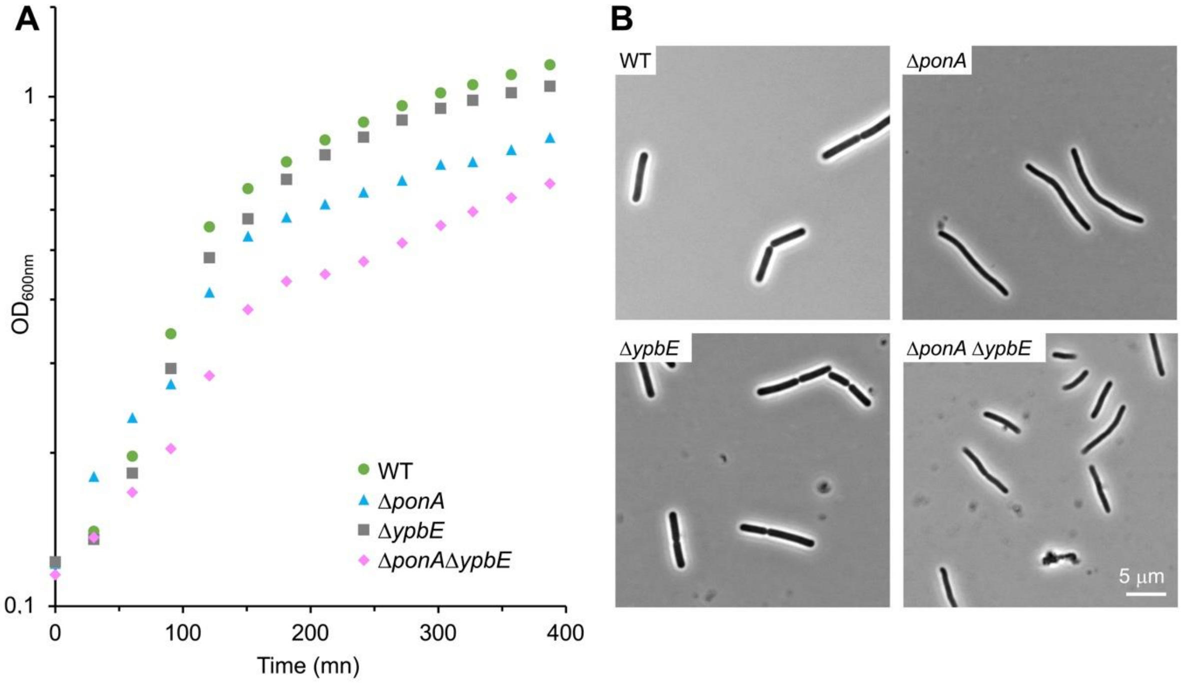

Appendix Figure S1 : Growth and morphology of strains deleted for *ypbE*.

To monitor growth of the different strains tested, adequate volume of a pre-culture in LB supplemented with 15 mM $MgSO_4$ was used to inoculate 150 µl of LB at initial OD_{600nm} = 0.1 in triplicate on a 96-well plate and incubated on a microplate reader at 37°C for 13 h with stirring and OD measurements every 30 minutes. The representative growth curves of 3 independent experiments are shown. **A.** Growth of strains WT168 (wild-type) in green, PS2062 (*ponA::spec*) in blue, BKE23000 (*ypbE::erm*) in grey and SG865 (*ponA::spec ypbE::erm*) in pink. **B.** Microscopy images of strains WT168, PS2062 (*ponA::spec*), BKE23000 (*ypbE::erm*) and SG865 (*ponA::spec ypbE::erm*) cultured in LB at 37°C up to OD_{600nm} = 0.3 were taken to analyse their cell morphology.

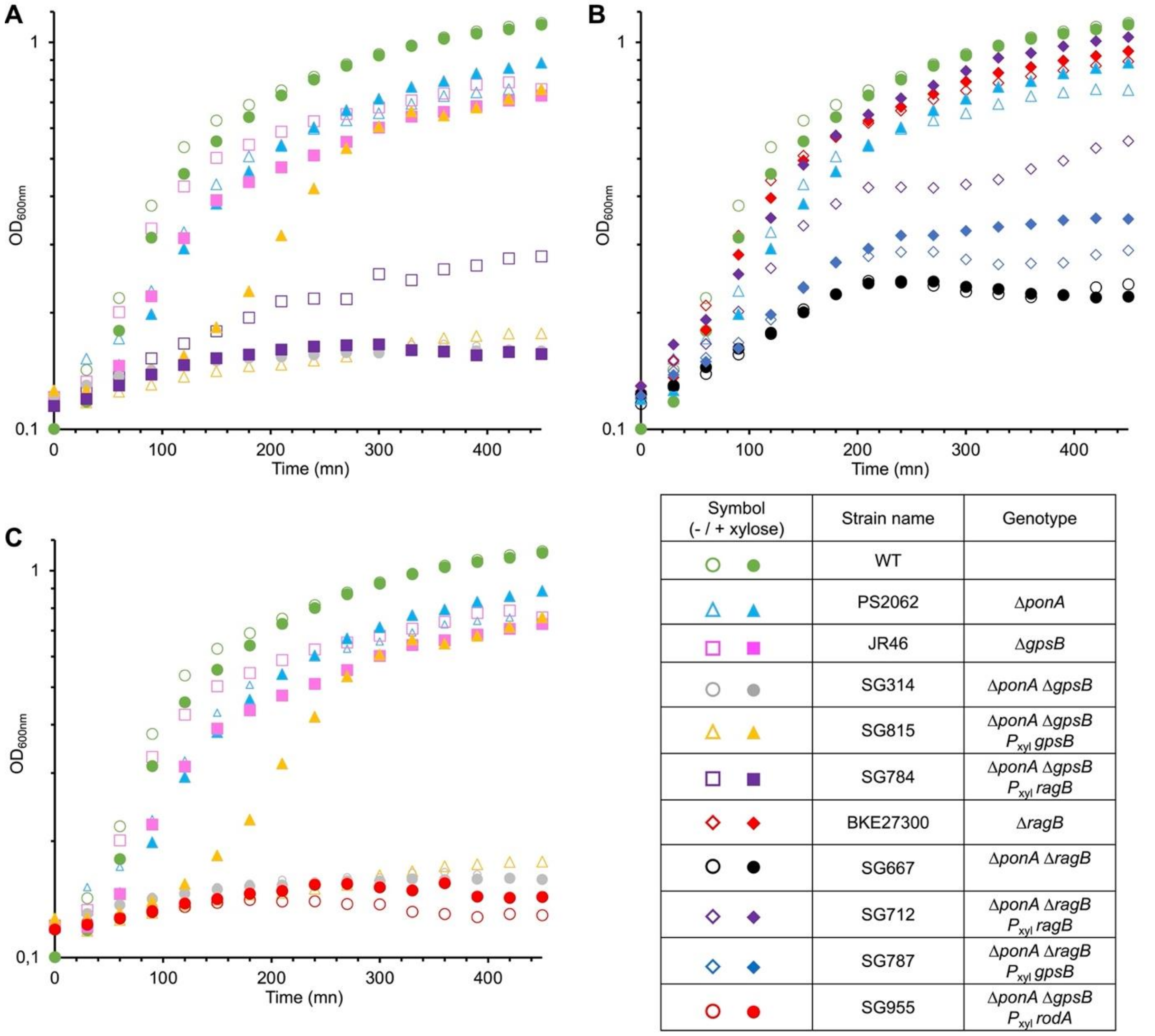

Appendix Figure S2 : Complementation of (*ponA*::*spec gpsB*::*kan*) and (*ponA*::*spec ragB*::*erm*) growths by GpsB, RagB or RodA overproduction.

To monitor growth of the different strains tested, adequate volume of a pre-culture in LB supplemented with 15 mM $MgSO_4$ was used to inoculate 150 µl of LB with (solid symbols) or without (empty symbols) 1% xylose at initial OD_{600nm} = 0.1 in triplicate on a 96-well plate and incubated on a microplate reader at 37°C for 7 h with stirring and OD measurements every 30 minutes. The growth curves shown are an average of 3 independent experiments. **A**. Complementation of (*ponA*::*spec gpsB*::*kan*) (in grey) growth defect by GpsB (in yellow) or RagB (in purple) overproduction. **B**. Complementation of (*ponA*::*spec ragB*::*erm*) (in black) growth defect by GpsB (in blue) or RagB (in purple) overproduction. **C**. Complementation of (*ponA*::*spec gpsB*::*kan*) (in grey) growth defect by GpsB (in yellow) or RodA (in red) overproduction.

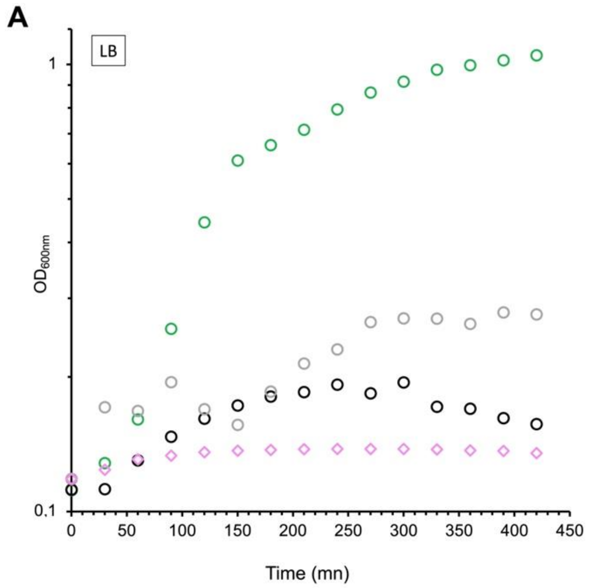

Symbol (- / + xylose)	Strain name	Genotype
○ ●	WT	
○	SG314	Δ*ponA* Δ*gpsB*
○	SG667	Δ*ponA* Δ*ragB*
◇ ◆	SG759	Δ*ponA* Δ*gpsB* Δ*ragB*
○ ●	SG869	Δ*ponA* Δ*gpsB* Δ*ragB* P_{xyl} *rodA*
△ ▲	SG959	Δ*ponA* Δ*gpsB* Δ*ragB* P_{xyl} *ponA*
□ ■	SG960	Δ*ponA* Δ*gpsB* Δ*ragB* P_{xyl} *ragB*
○ ●	SG961	Δ*ponA* Δ*gpsB* Δ*ragB* P_{xyl} *gpsB*

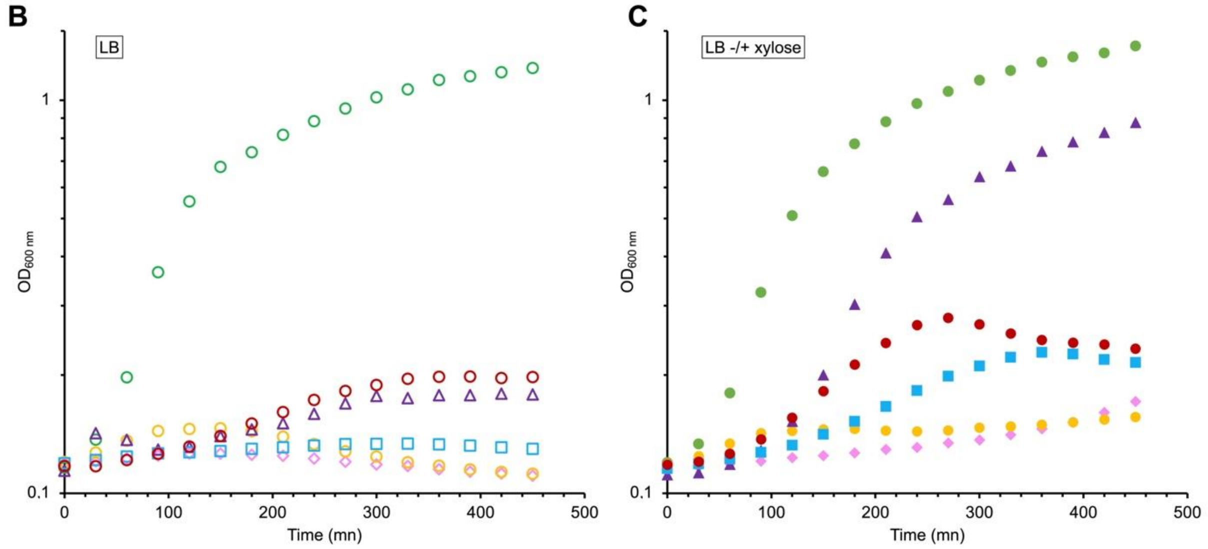

Appendix Figure S3 : Comparison of (*ponA*::*spec gpsB*::*kan*), (*ponA*::*spec ragB*::*erm*) and (*ponA*::*spec gpsB*::*kan ragB*::*erm*) growths and complementation by PBP1, GpsB, RagB or RodA overproduction.

To monitor growth of the different strains tested, adequate volume of a pre-culture in LB supplemented with 15 mM $MgSO_4$ was used to inoculate 150 µl of LB with (solid symbols) or without (empty symbols) 1% xylose at initial OD_{600nm} = 0.1 in triplicate on a 96-well plate and incubated on a microplate reader at 37°C for 8 h with stirring and OD measurements every 30 minutes. The growth curves shown are an average of 3 independent experiments. **A**. Comparison of (*ponA*::*spec gpsB*::*kan*) (in black), (*ponA*::*spec ragB*::*erm*) (in grey) and (*ponA*::*spec ragB*::*erm gpsB*::*kan*) (in pink) growth defect with the wild-type (in green). **B** and **C**. Complementation of (*ponA*::*spec ragB*::*erm gpsB*::*kan*) (in pink) growth defect by GpsB (in red), RagB (in blue), PBP1 (in purple) or RodA (in yellow) overproduction by xylose induction (**C**) compared to LB (**B**).

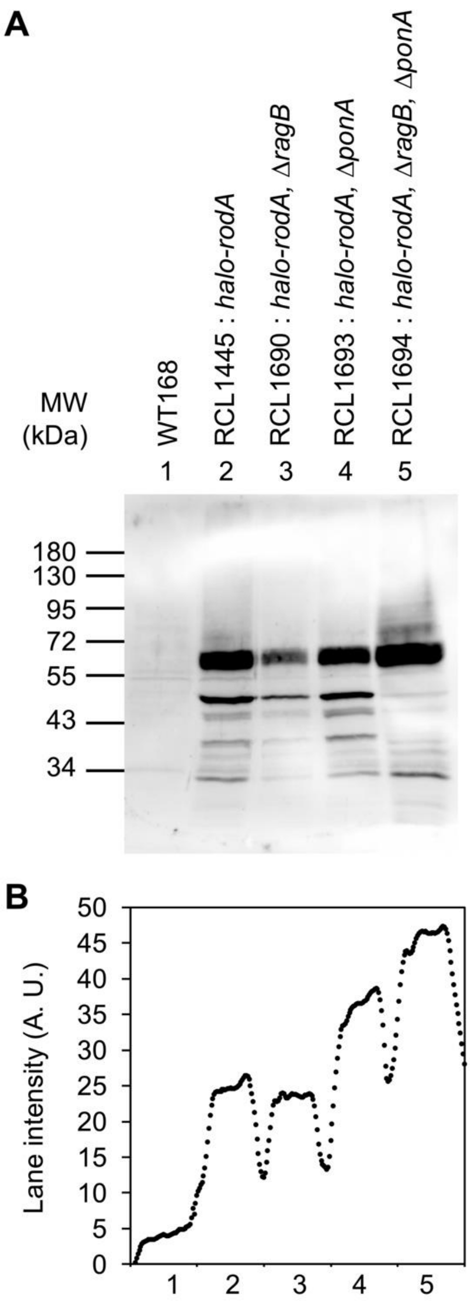

Appendix Figure S4 : Western blot detecting Halo-tagged RodA in several genetic contexts.

A. Comparative western blot showing Halo-RodA in wild-type (RCL1445, lane 2), *ragB*::*kan* (RCL1690, lane 3), *ponA*::*spec* (RCL1693, lane 4) and *ponA*::*spec ragB*::*kan* (RCL1694, lane 5) genetic contexts and in a wild-type strain (WT168, lane 1) as negative control. Strains were grown in LB medium until OD_{600nm} = 0.6 at 37°C, crude extracts were loaded (15 µl) and separated on 10% SDS-PAGE, transferred to a nitrocellulose membrane and RodA was detected using monoclonal anti-Halo-tag antibodies (Promega, 1/1000e). **B**. Average intensity (in A.U.) of chemiluminescence signal quantified for each strain from 4 different western-blots.

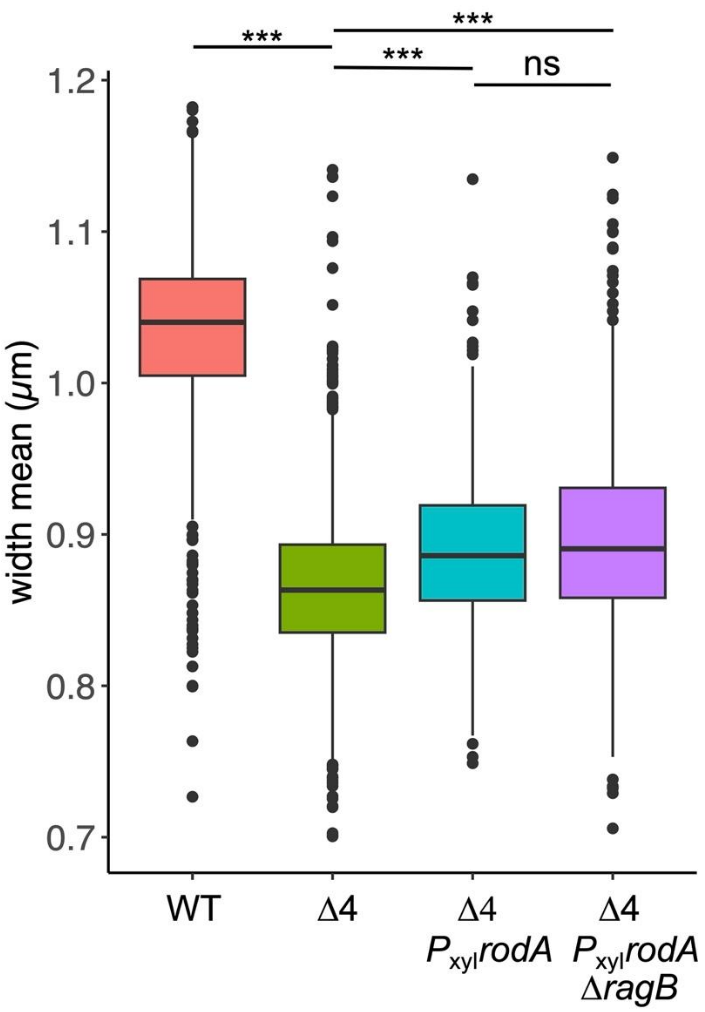

Appendix Figure S5 : Statistical analysis of cell diameters for the Δ4 strain and its derivatives.

A. Visible light microscopy images were taken for the 4 strains wild-type 168 (WT), AG157 (*pbpG::kan ΔpbpD ΔpbpF ΔponA* named Δ4), SG1150 (Δ4 *amyE*::P_{xyl}*rodA*) and SG1158 (Δ4 *ragB::erm amyE*::P_{xyl}*rodA*) grown in LB supplemented with 2.5 mM $MgSO_4$ and 0.5% xylose until OD_{600nm} of 0.4 at 37°C. Ten images per strain were taken in order to be able to statistically process the morphology parameters in a significant number of cells. Data were collected from 3 independent experiments and the width mean of the cells was measured for each strain. Statistically significant differences are indicated (ns = not significant, *** = p<0.001).

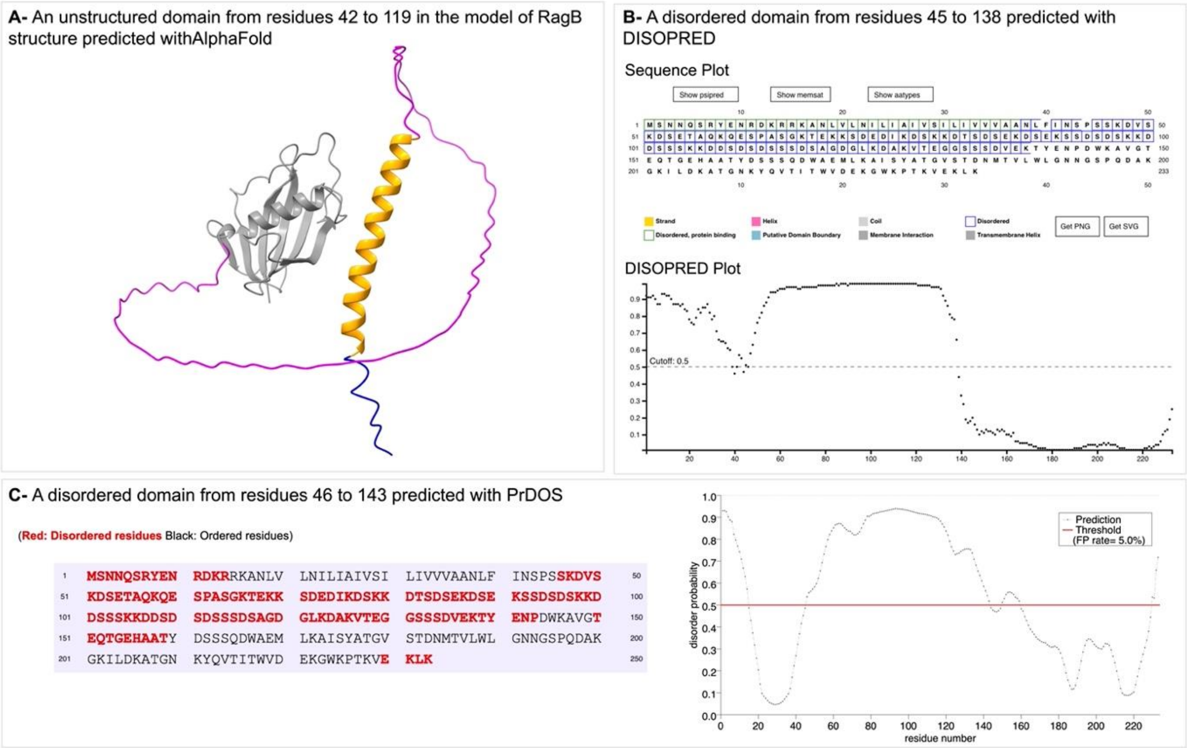

Appendix Figure S6 : RagB is a transmembrane protein with a disordered domain.

Various structure prediction softwares were used to understand how the 3D structure of RagB could be organized. **A.** AlphaFold software predicted a 3D structure with a short cytoplasmic N-terminal sequence (15 amino acids in blue) followed by a transmembrane helix of around 30 amino acids (in yellow) from which a long (77 aa) unstructured domain extends (in pink), and finally a C-terminal domain of unknwown function (DUF1510, in grey) composed of several β-strands and one α-helix. **B.** With DISOPRED software (Jones & Cozzetto, 2015), we identified an intrinsically disordered region (IDR) from residue 45 to 138. **C.** Using PrDOS software (Ishida & Kinoshita, 2007), we confirmed the overall structure organization and highlighted the residues (in red) belonging to disordered regions in the RagB protein. Some are present in the N-terminal region but most of them are located in the domain identified as IDR with DISOPRED. It consists mainly of residues serine (25%), aspartic acid (19%), lysine (17%), Glutamic acid (8%), and Threonine (5%).

Corresponding references:

Ishida T, Kinoshita K (2007) PrDOS: prediction of disordered protein regions from amino acid sequence. *Nucleic Acids Res* 35: W460-464
Jones DT, Cozzetto D (2015) DISOPRED3: precise disordered region predictions with annotated protein-binding activity. *Bioinformatics* 31: 857-863

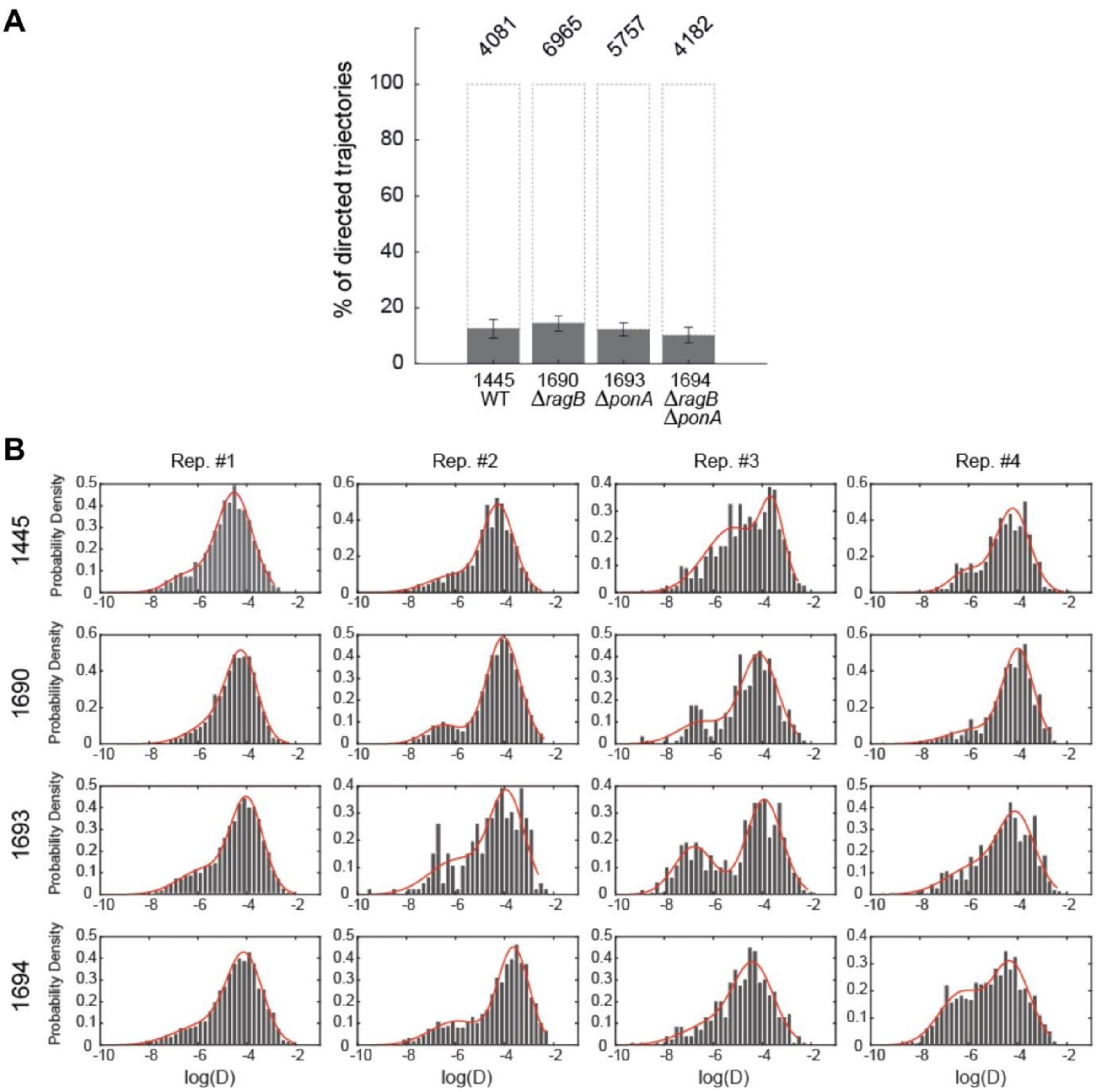

Appendix Figure S7 : MSD and CDF analysis of RodA-Halo trajectories observed by SPT

Trajectories of RodA-Halo (SPT) over 4 replicas observed in the wild-type strain (1445) and in the Δ*ragB* (1690), Δ*ponA* (1693) and Δ*ragB* Δ*ponA* (1694) mutants grown on s-EZRDM. **A**. Average fraction of single particles per cell displaying directional motion in each strain, determined by mean square displacement (MSD) analysis. Error bars correspond to the standard deviation. Data are a compilation of four independent experiments. Numbers on top of the bars indicate the total number of trajectories in the pooled replicas. **B**. Bimodal distribution of diffusion coefficients (DC) determined by CDF analysis. DC were generated on single tracks of non-directional single particles using a one-component model. Each plot correspond to one of the four replicas in one of the four strains. Red lines are fits to the

distributions using maximum likelihood estimates with a model that employs a mixture of two distinct log-normal distributions.

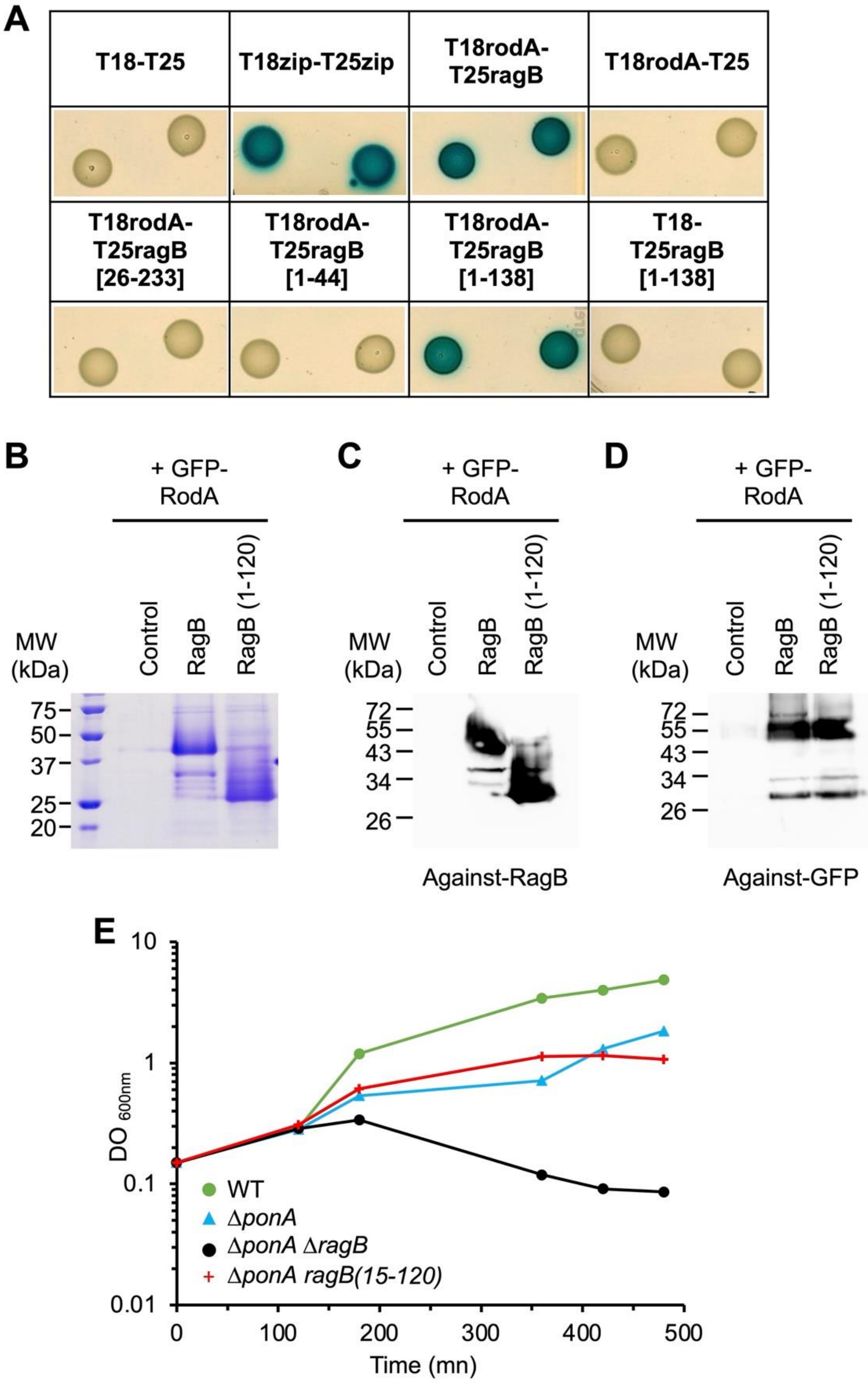

Appendix Figure S8 : The TM and IDR domains of RagB are necessary for the interaction with RodA *in vitro* and its function *in vivo*.

A. Detection of protein interactions by bacterial two-hybrid assay. The T18 and T25 fragments of the adenyl cyclase protein were fused to the N-termini of the full length or truncated versions of RagB and RodA. Co-transformed strains of *E. coli BTH101* were spotted onto LB medium supplemented with Xgal

and IPTG and incubated at 30°C overnight. Blue colonies indicate a positive interaction between RodA and RagB[1-138] containing its TM and IDR domains but no interaction was detected between RodA and RagB[1-44] missing its full extracytoplasmic domain (IDR + DUF1515) or RagB[26-233] missing its TM domain. **B, C, D.** Detection of protein interactions by pull-down assay. An extract of membrane proteins from the SG825 (*ragB::erm amyE::*P_{xyl}*rodA-gfp*) strain containing RodA-GFP was incubated for 1 h at 4°C with either buffer (control lanes) or 100 µg of 6His-RagB or 6His-RagB[1-120] protein and then purified on a Ni-NTA column. The purification fractions were separated by SDS-PAGE and stained with Coomassie blue (**B**) or transferred to a nitrocellulose membrane for western blot detection with anti-RagB (**C**) or anti-GFP (**D**) antibodies. The elution of RagB and RagB[1-120] was confirmed using specific antibodies in (**C**). RodA-GFP does not bind to the Ni-NTA resin by itself as shown in the control lane in (**D**) but is co-eluted with either 6His-RagB or 6His-RagB[1-120] showing that the TM and IDR domains are sufficient for the interaction with RodA. **E.** RagB[15-120] is able to replace full length protein during bacterial growth. To monitor growth, few µl of a 1 ml pre-culture in LB supplemented with 15 mM $MgSO_4$ was used to inoculate 150 µl of LB at initial OD_{600nm} = 0.1 in triplicate on a 96-well plate and incubated on a microplate reader at 37°C for 8 h with stirring and OD measurements every 30 minutes. The representative growth curves of 3 independent experiments are shown. Growth of strains WT168 (wild-type) in green, PS2062 (*ponA::spec*) in blue, SG667 (*ponA::spec ragB::erm*) in black and SG865 (*ponA::spec ragB(1-120) kan*) in red.
