# Supplementary figures and images for: RagB stimulates the activity of the peptidoglycan polymerase RodA in Bacillus subtilis
Source: EMBO Rep. 2025 Aug 15;26(18):4587–606. doi: 10.1038/s44319-025-00547-w (PMC12457691; doi:10.1038/s44319-025-00547-w)

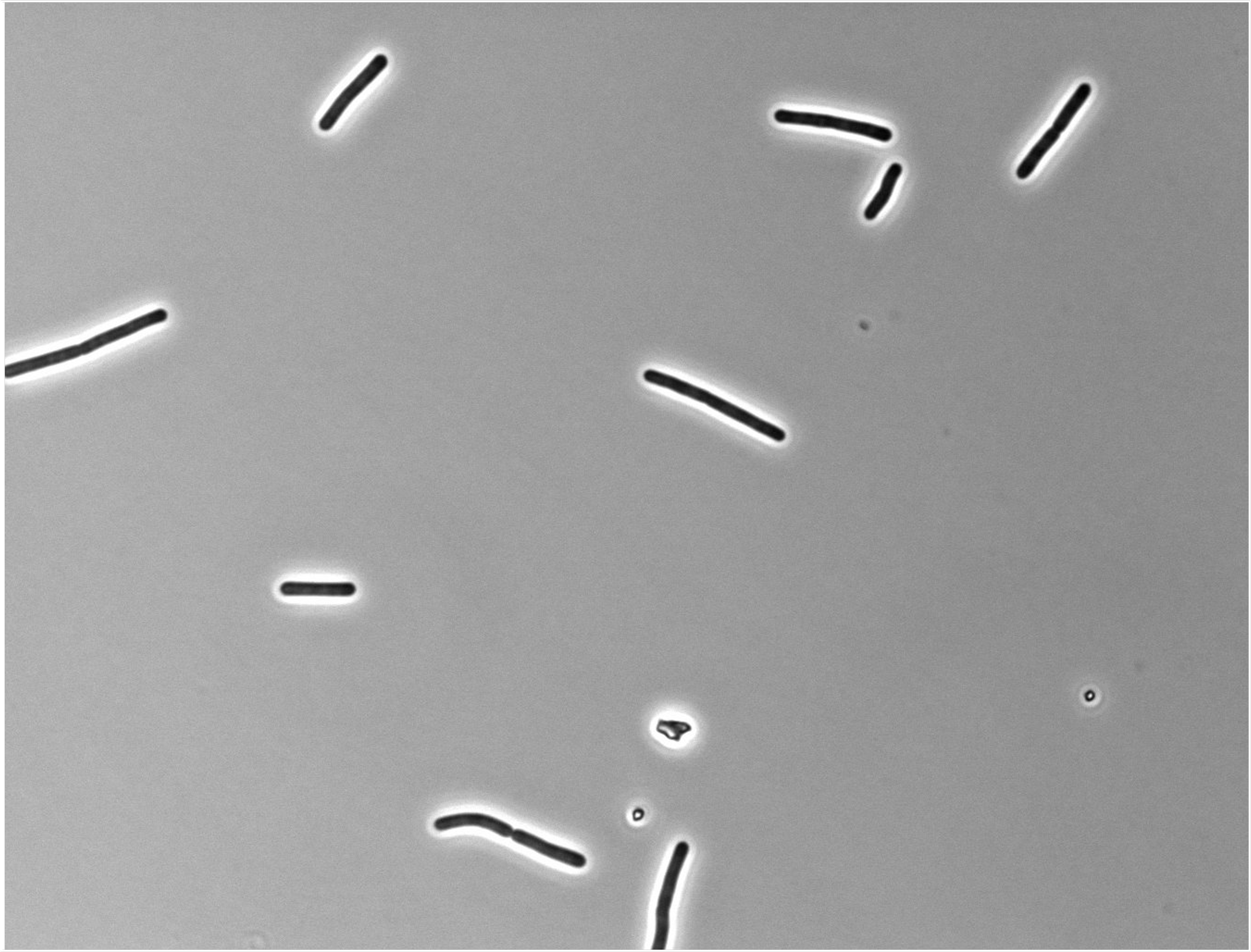

Supplement: Supplementary file 6 — Source data Fig. 1 [file 44319_2025_547_MOESM6_ESM.zip › Figure 1 /Fig1B/delta gpsB.tiff]

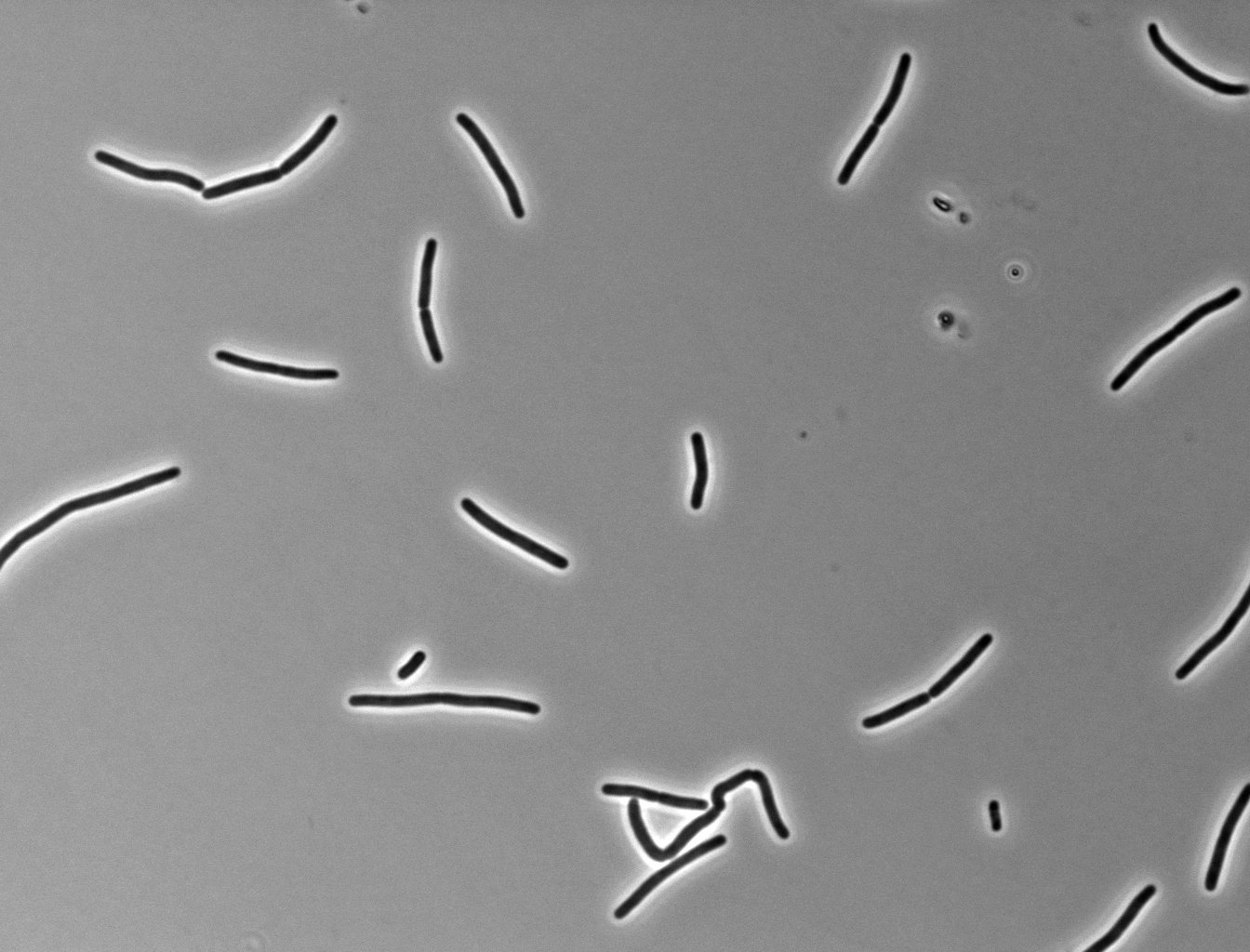

Supplement: Supplementary file 6 — Source data Fig. 1 [file 44319_2025_547_MOESM6_ESM.zip › Figure 1 /Fig1B/delta ponA.tiff]

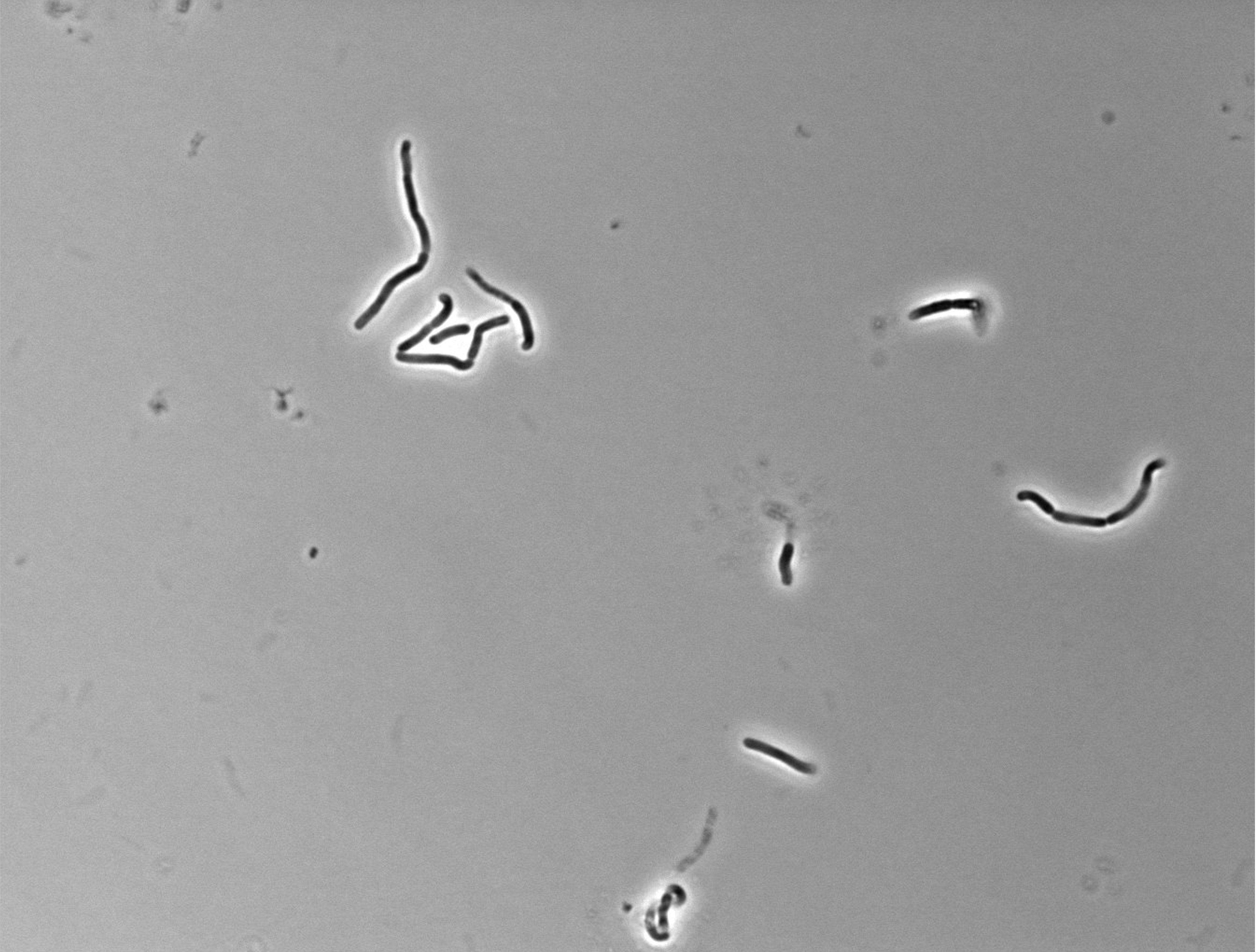

Supplement: Supplementary file 6 — Source data Fig. 1 [file 44319_2025_547_MOESM6_ESM.zip › Figure 1 /Fig1B/delta ponAdelta gpsB.tiff]

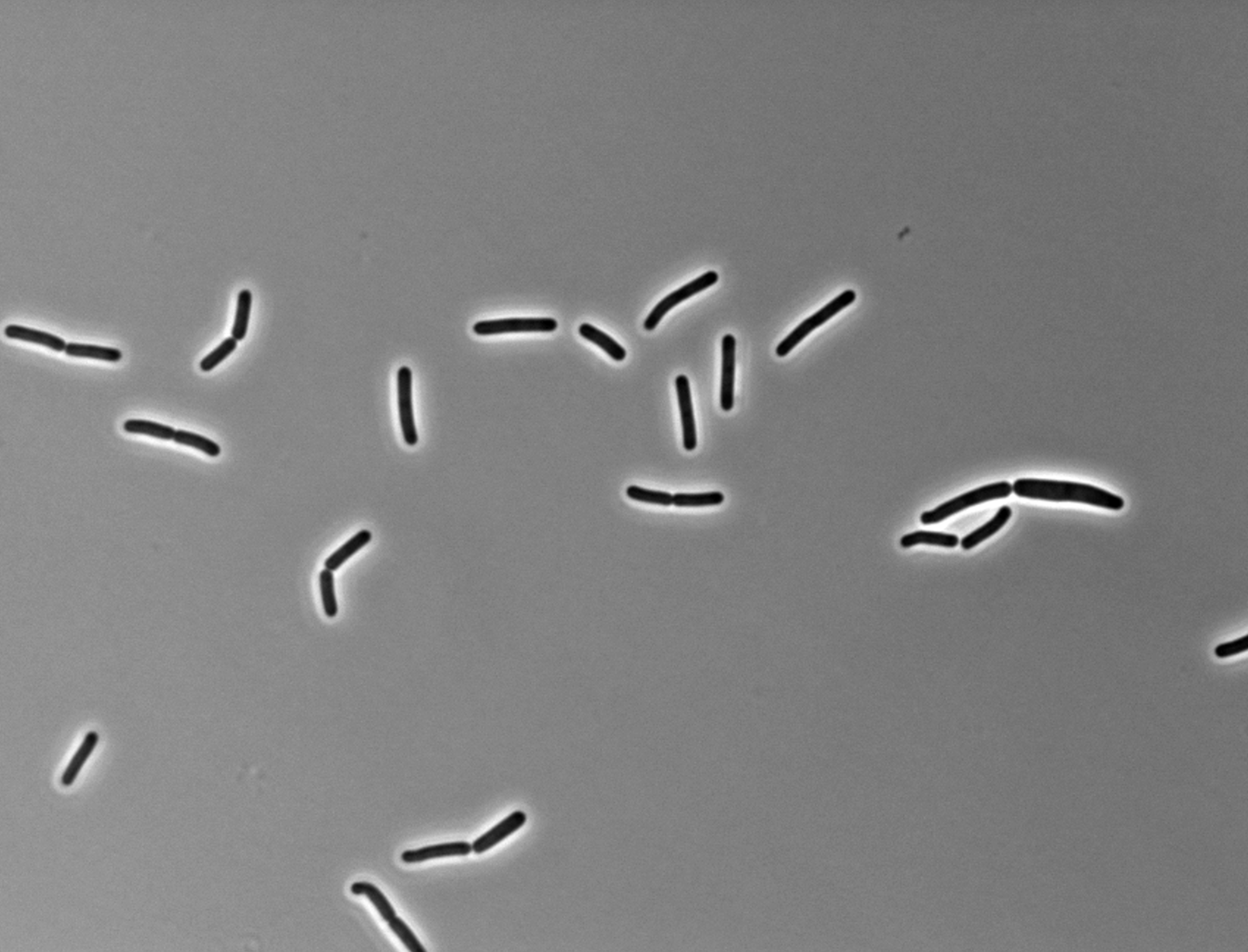

Supplement: Supplementary file 6 — Source data Fig. 1 [file 44319_2025_547_MOESM6_ESM.zip › Figure 1 /Fig1B/delta ragB.tiff]

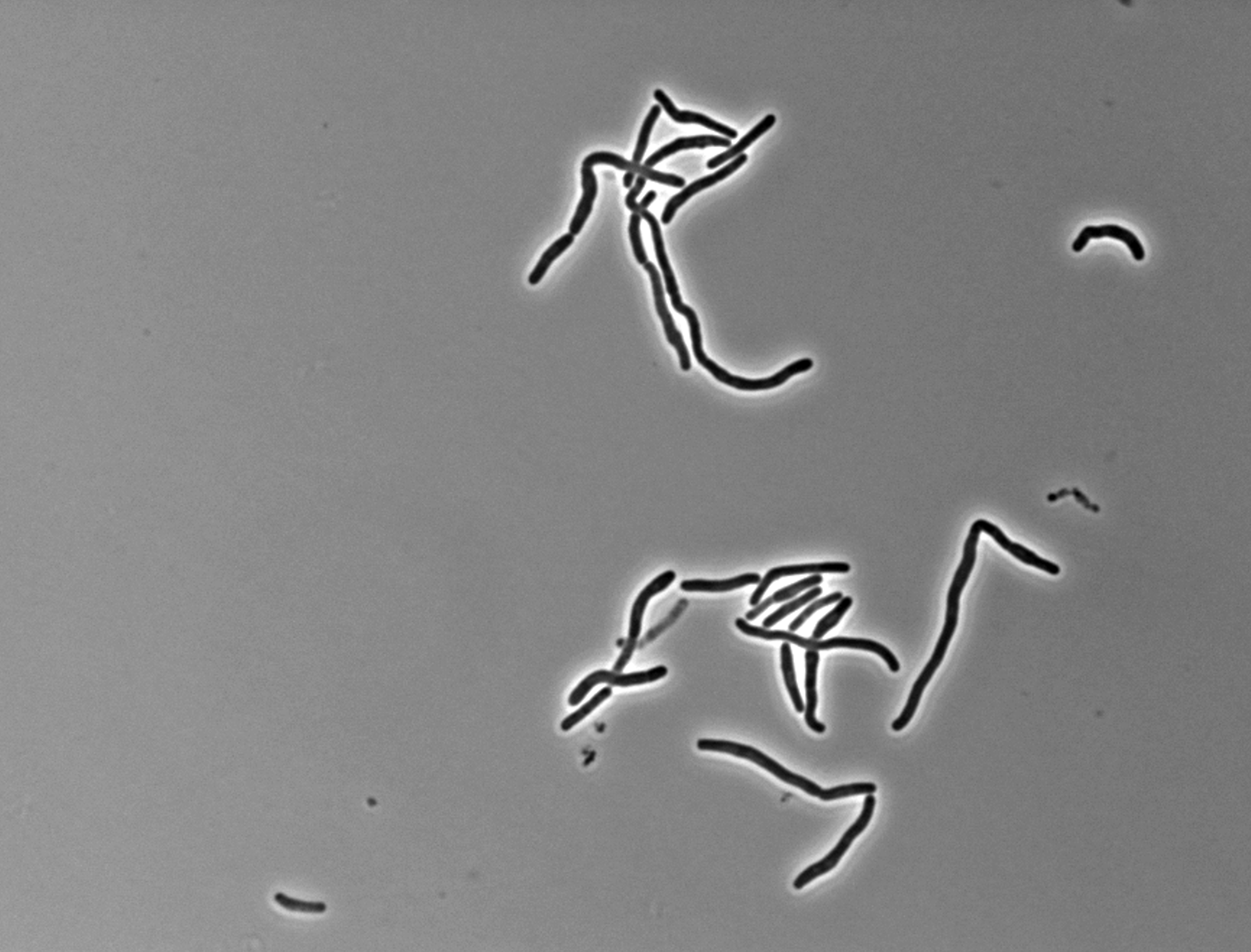

Supplement: Supplementary file 6 — Source data Fig. 1 [file 44319_2025_547_MOESM6_ESM.zip › Figure 1 /Fig1B/Deltapona deltaragB.tiff]

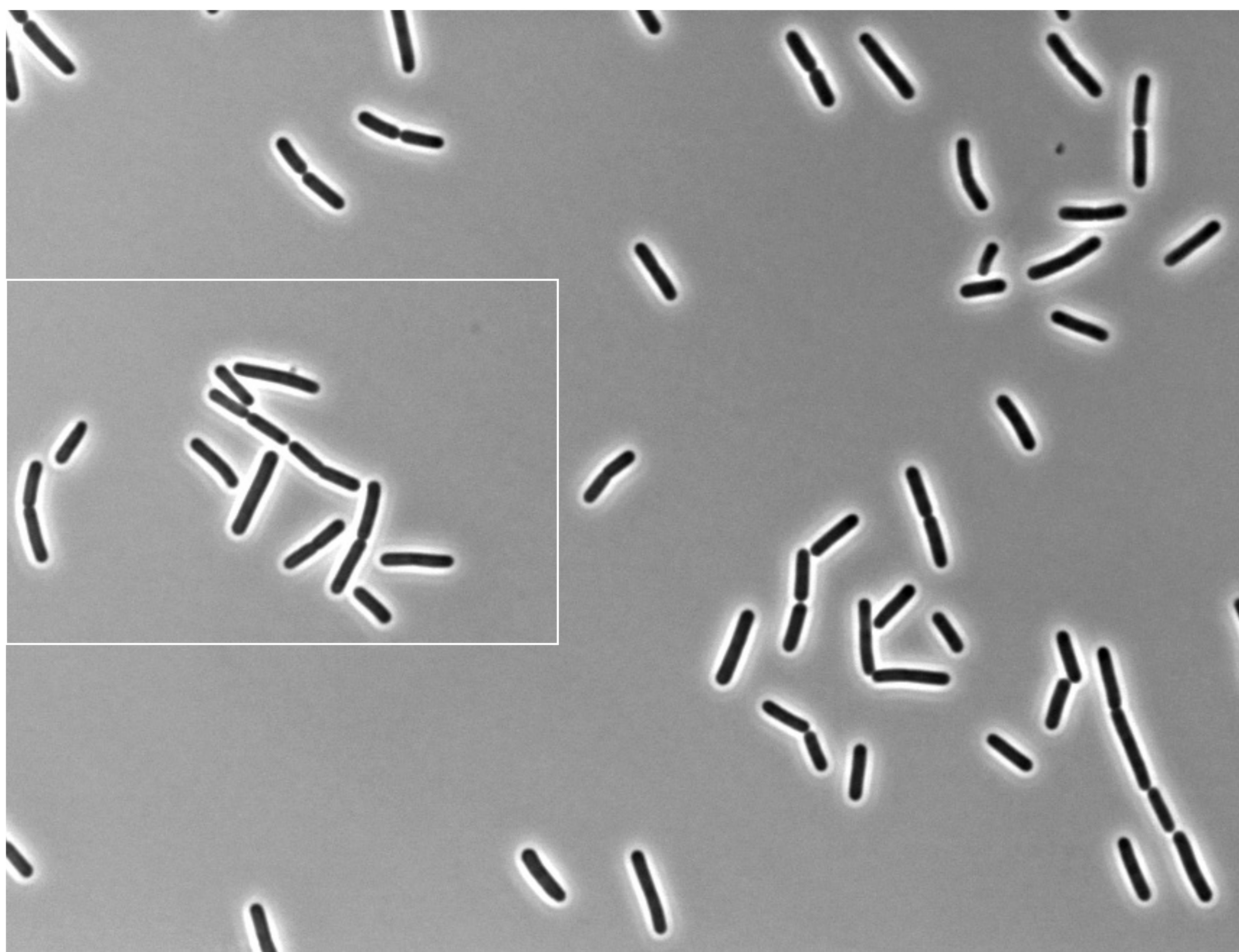

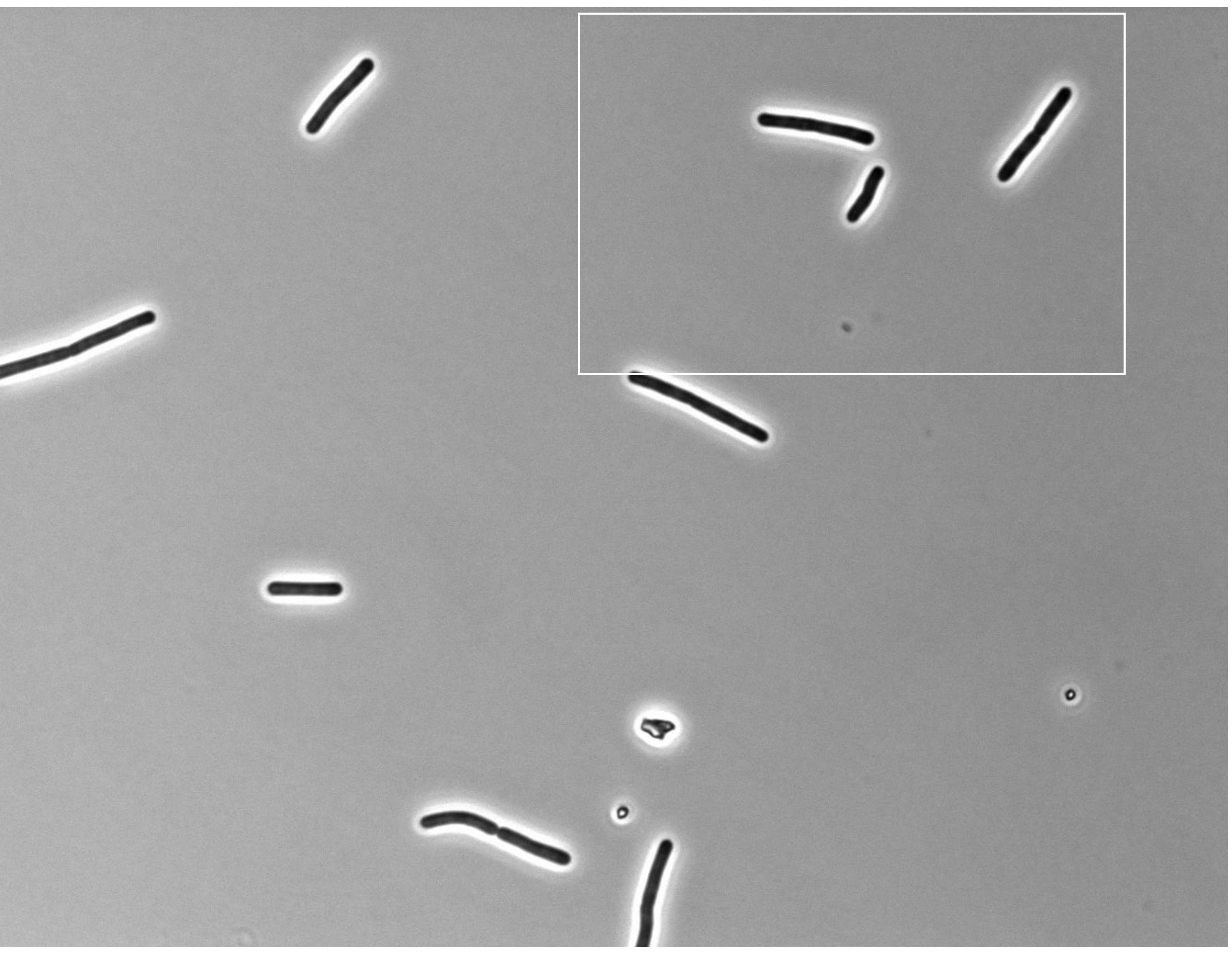

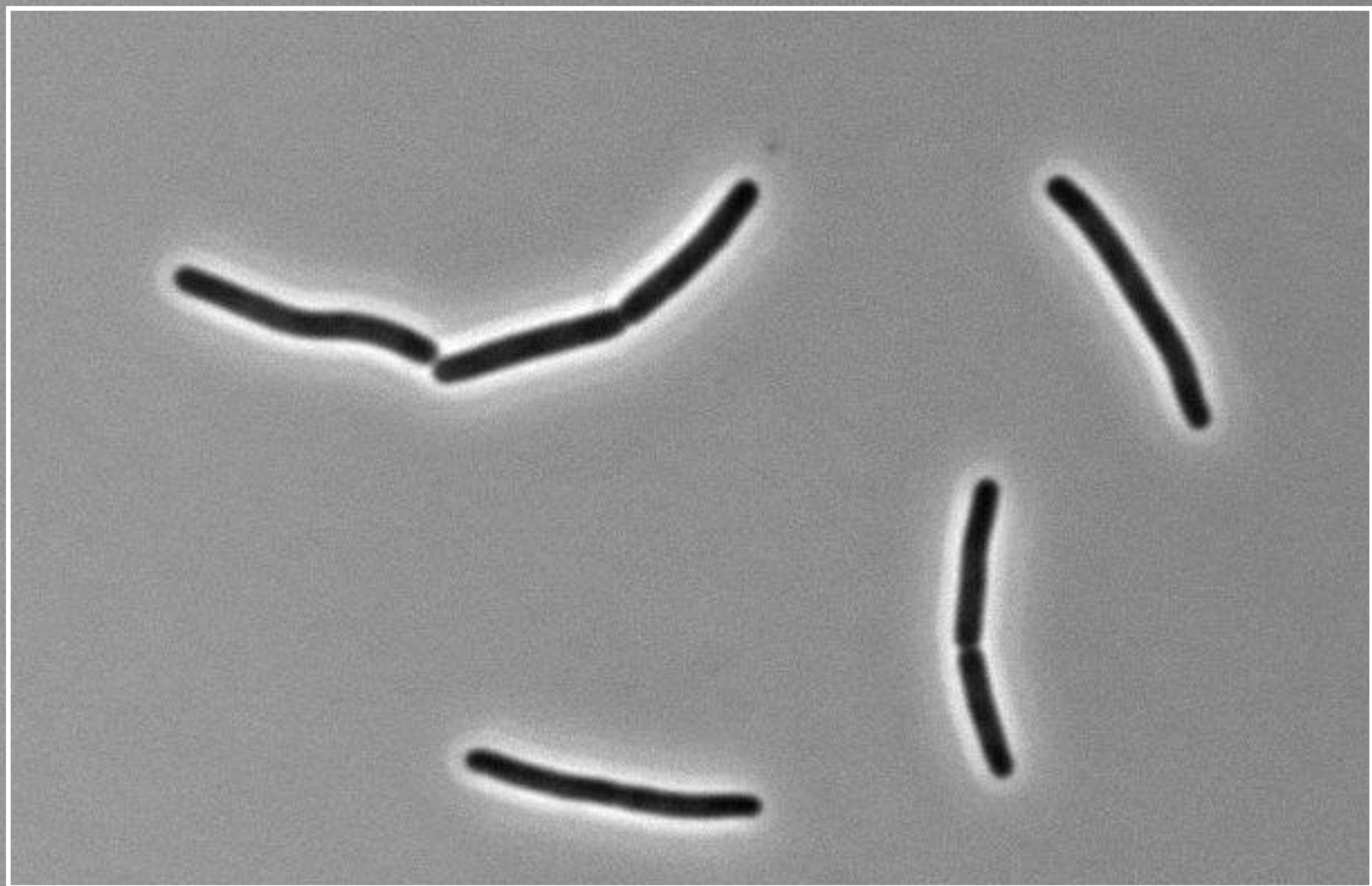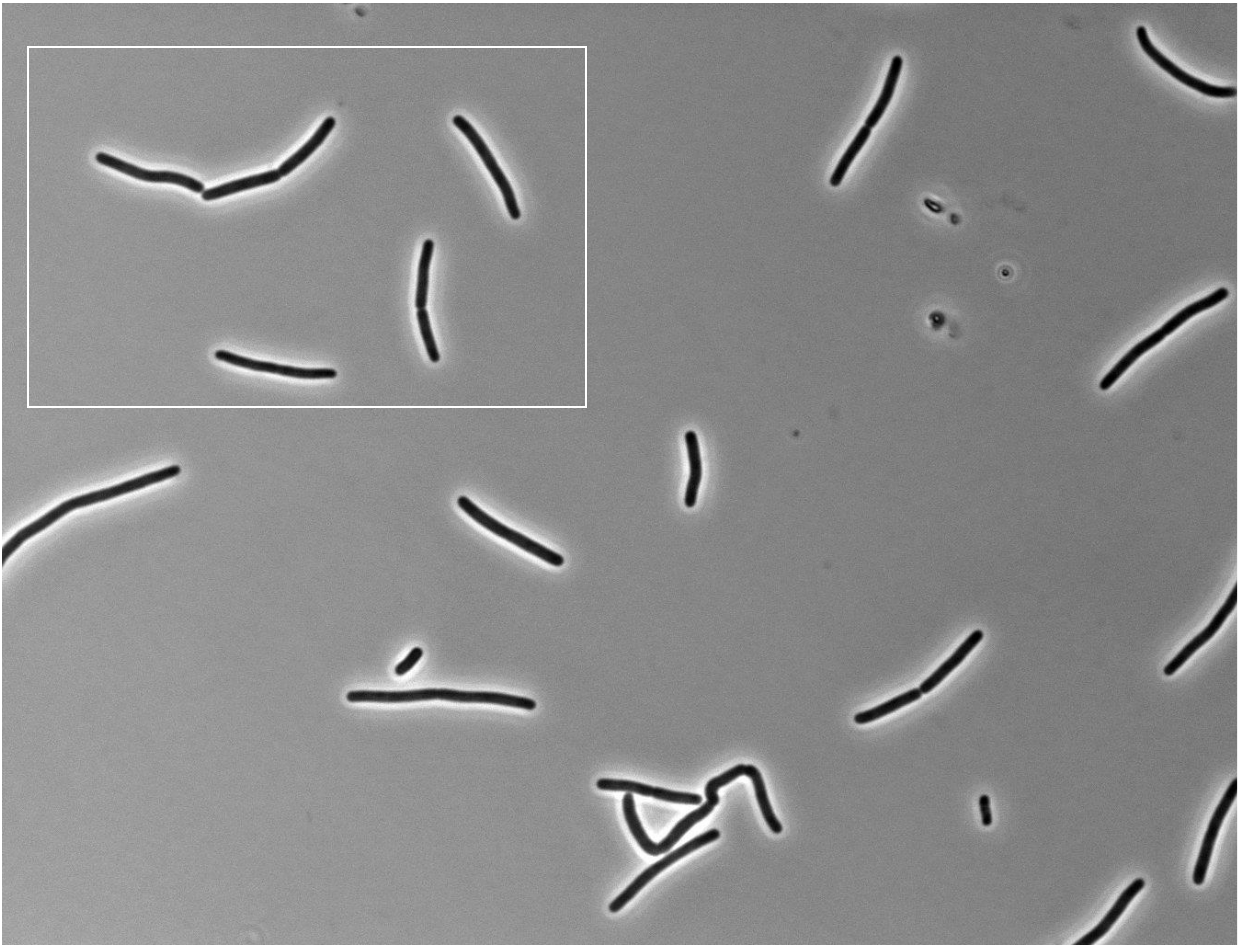

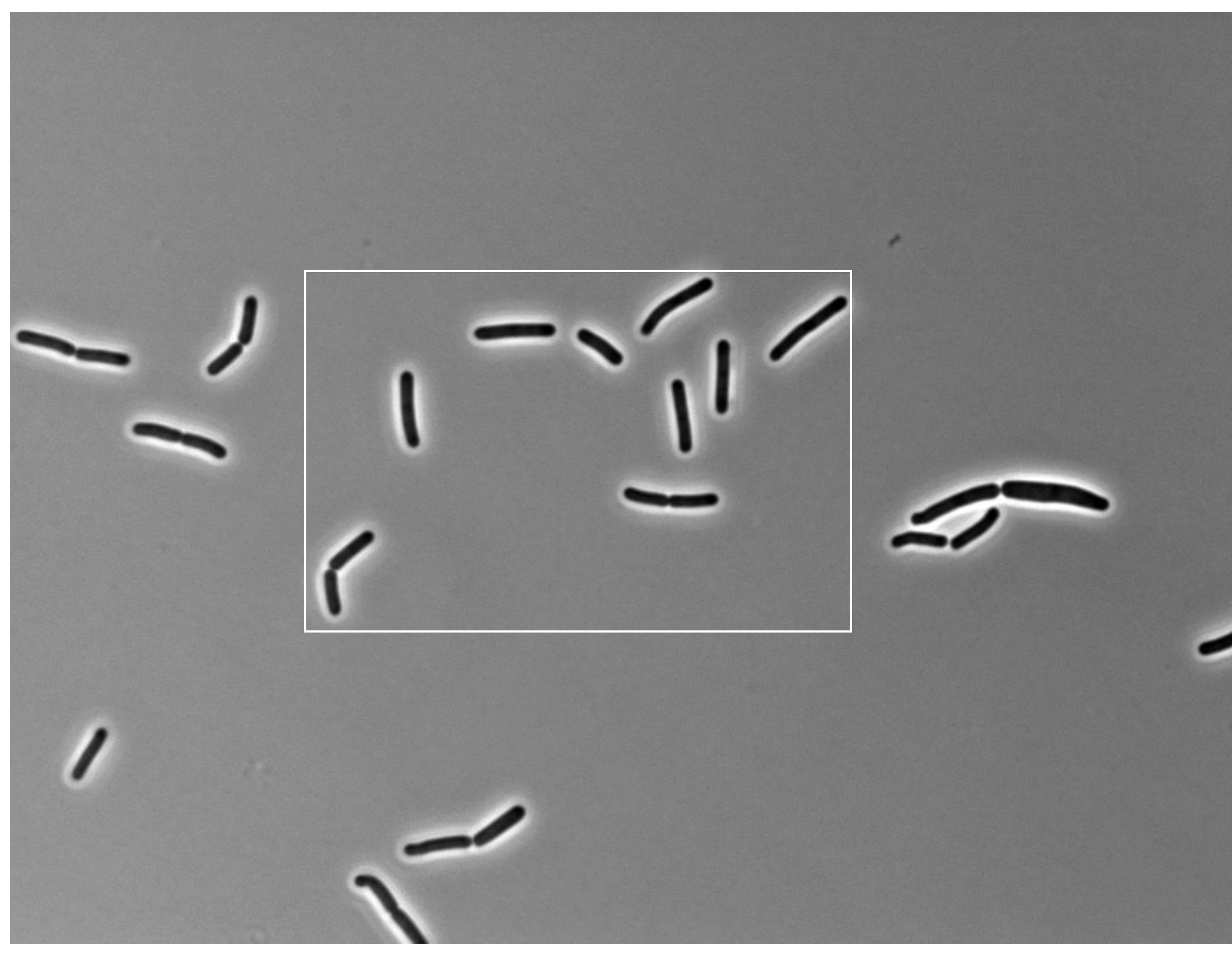

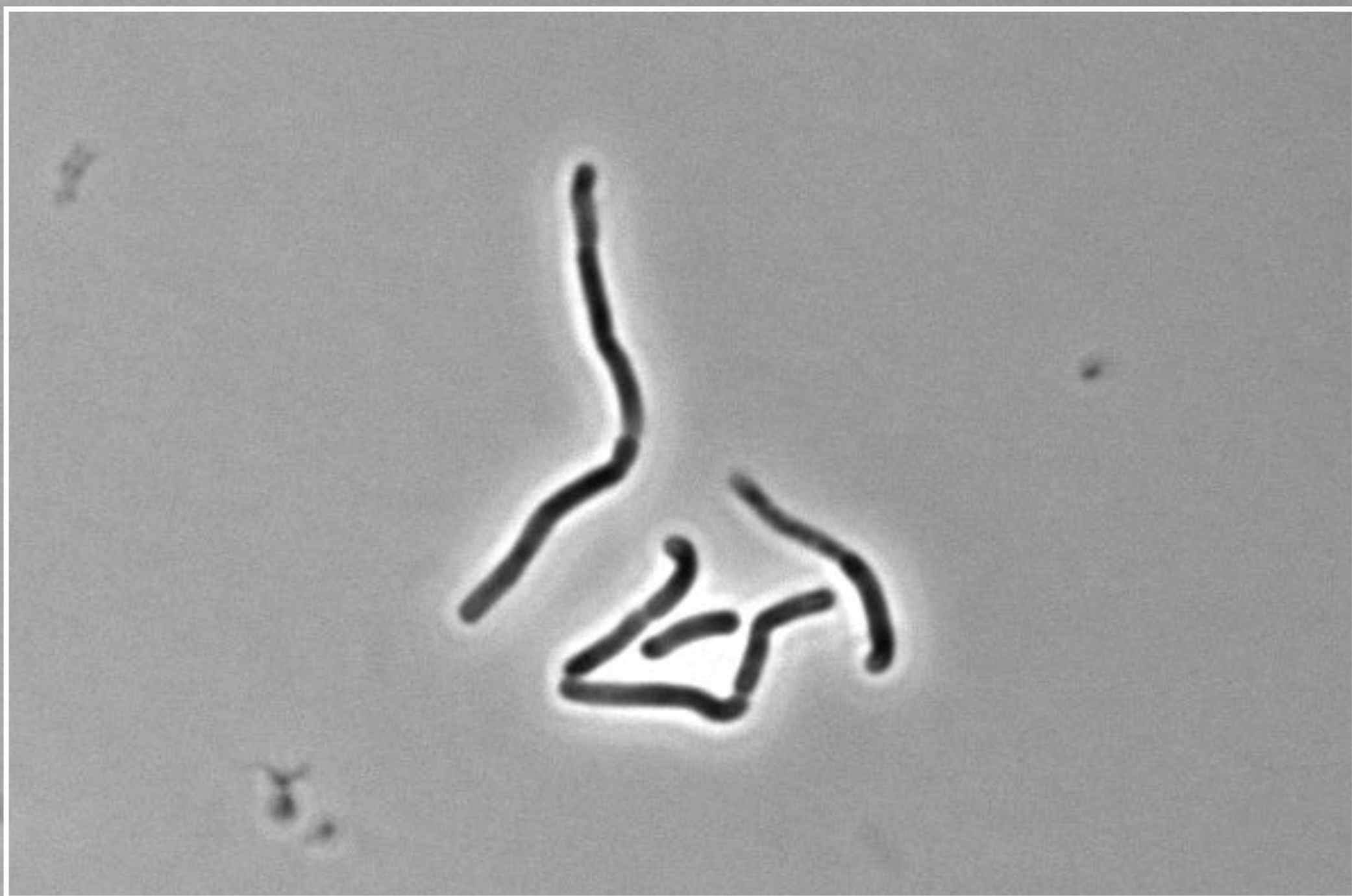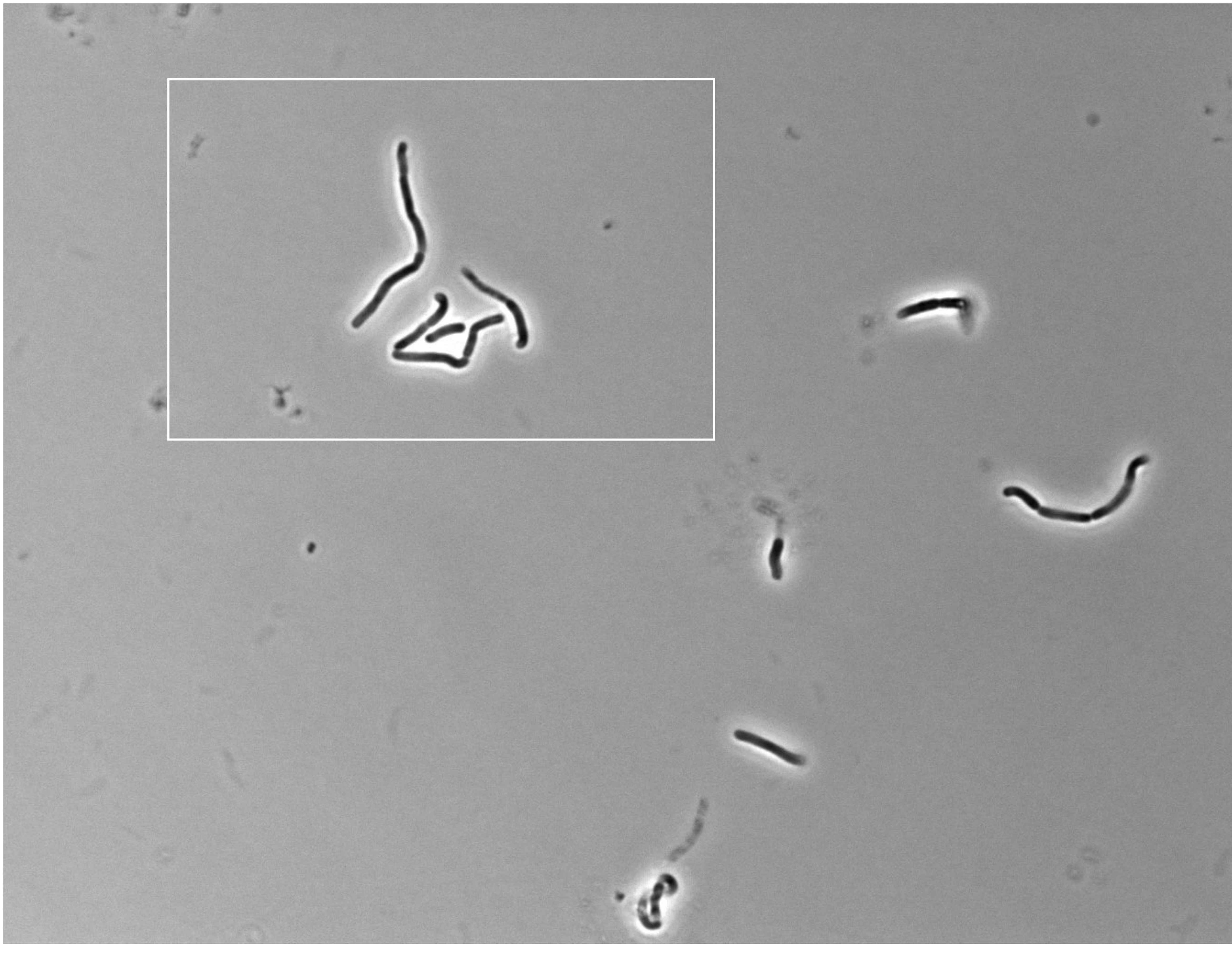

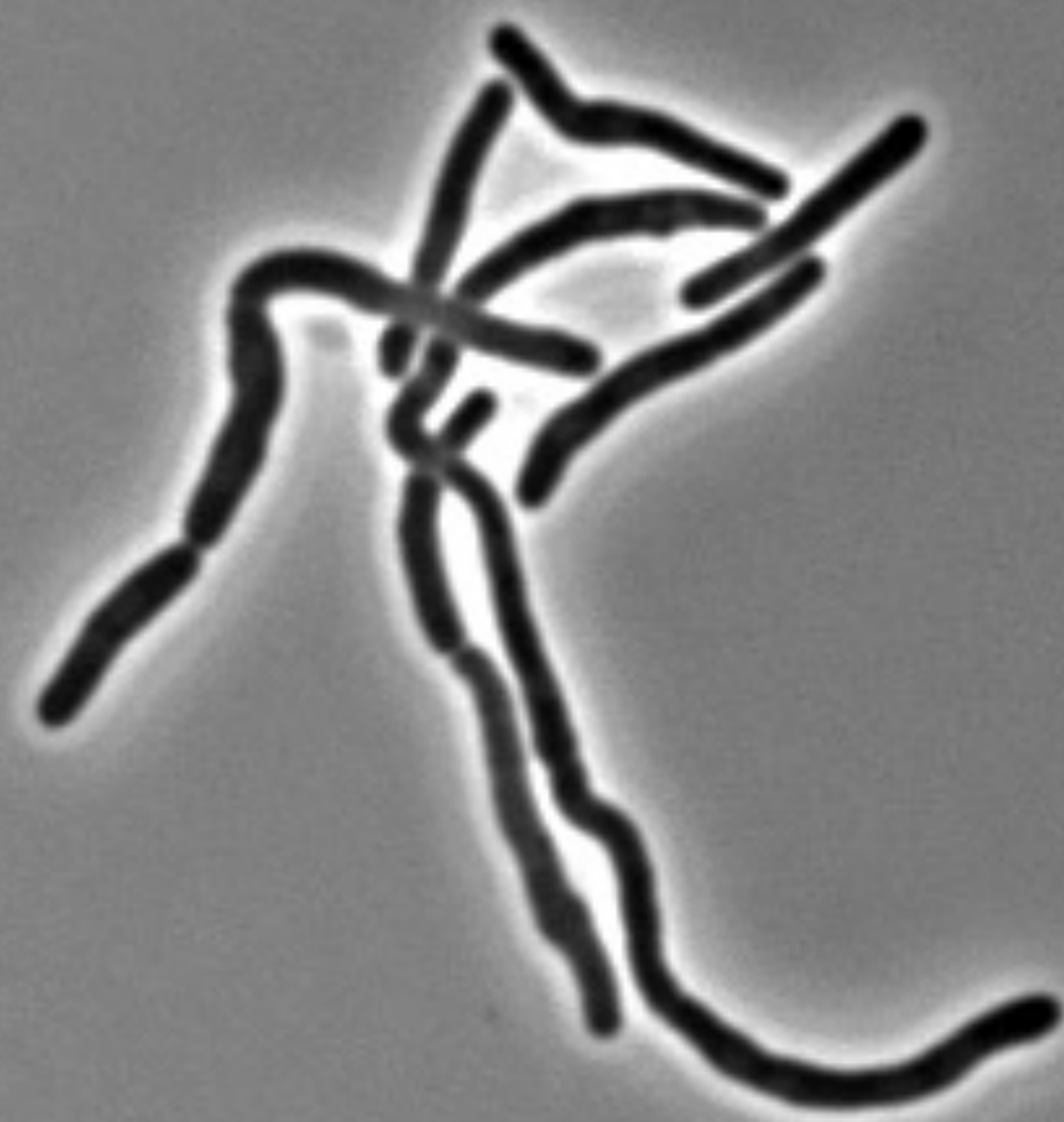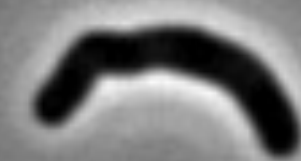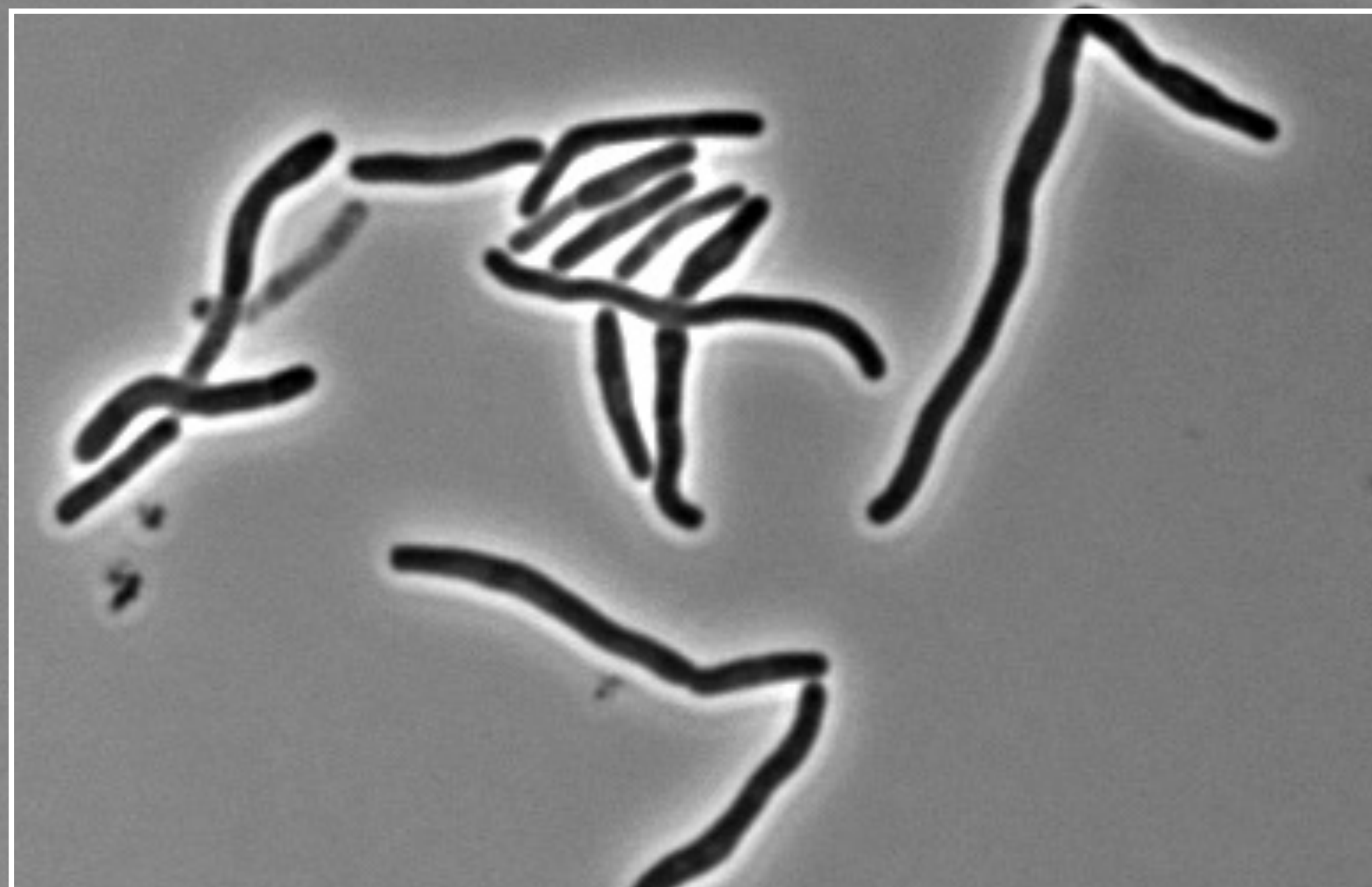

Supplement: Supplementary file 6 — Source data Fig. 1 [file 44319_2025_547_MOESM6_ESM.zip › Figure 1 /Fig1B/Fig.1B.pdf]

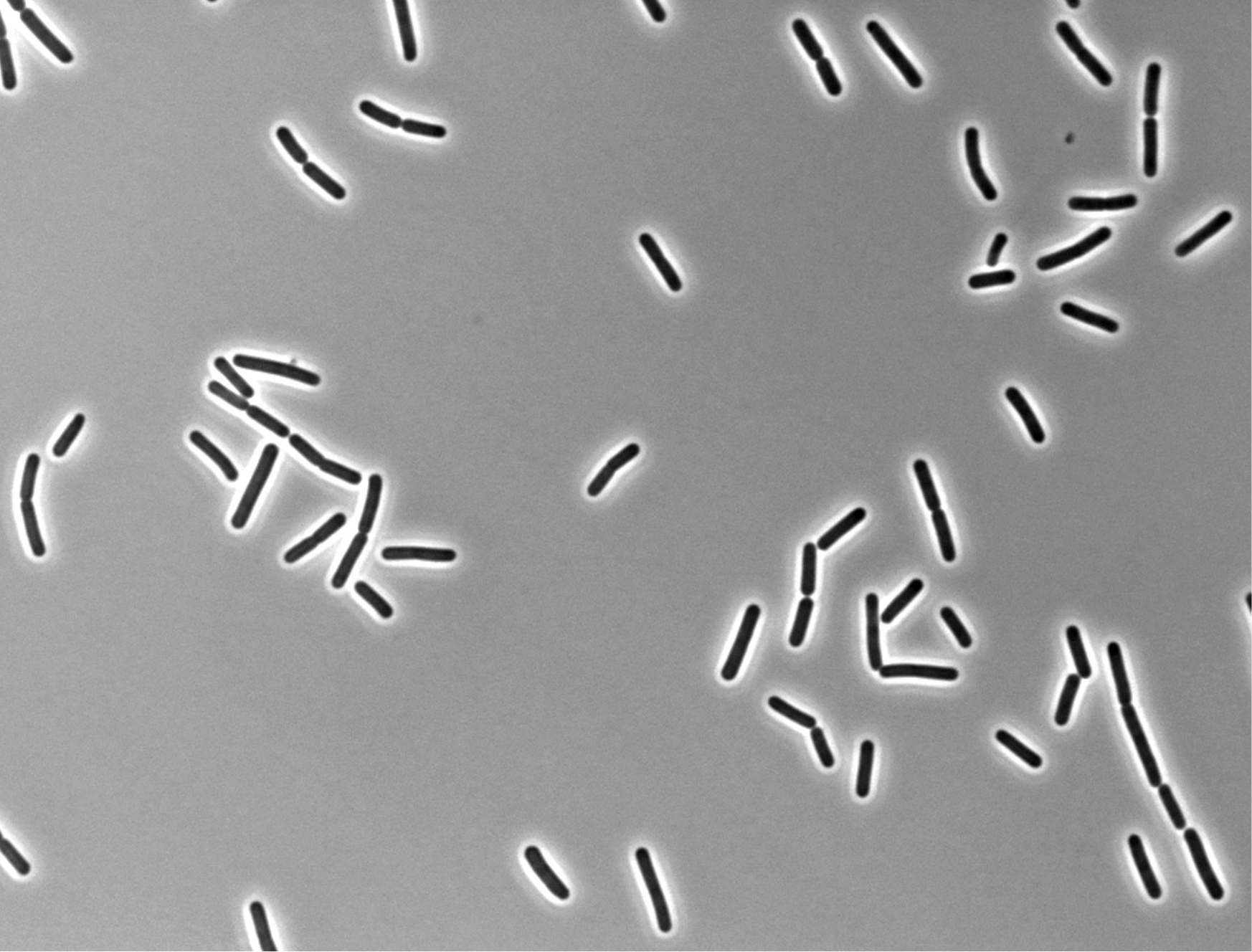

Supplement: Supplementary file 6 — Source data Fig. 1 [file 44319_2025_547_MOESM6_ESM.zip › Figure 1 /Fig1B/WT.tiff]

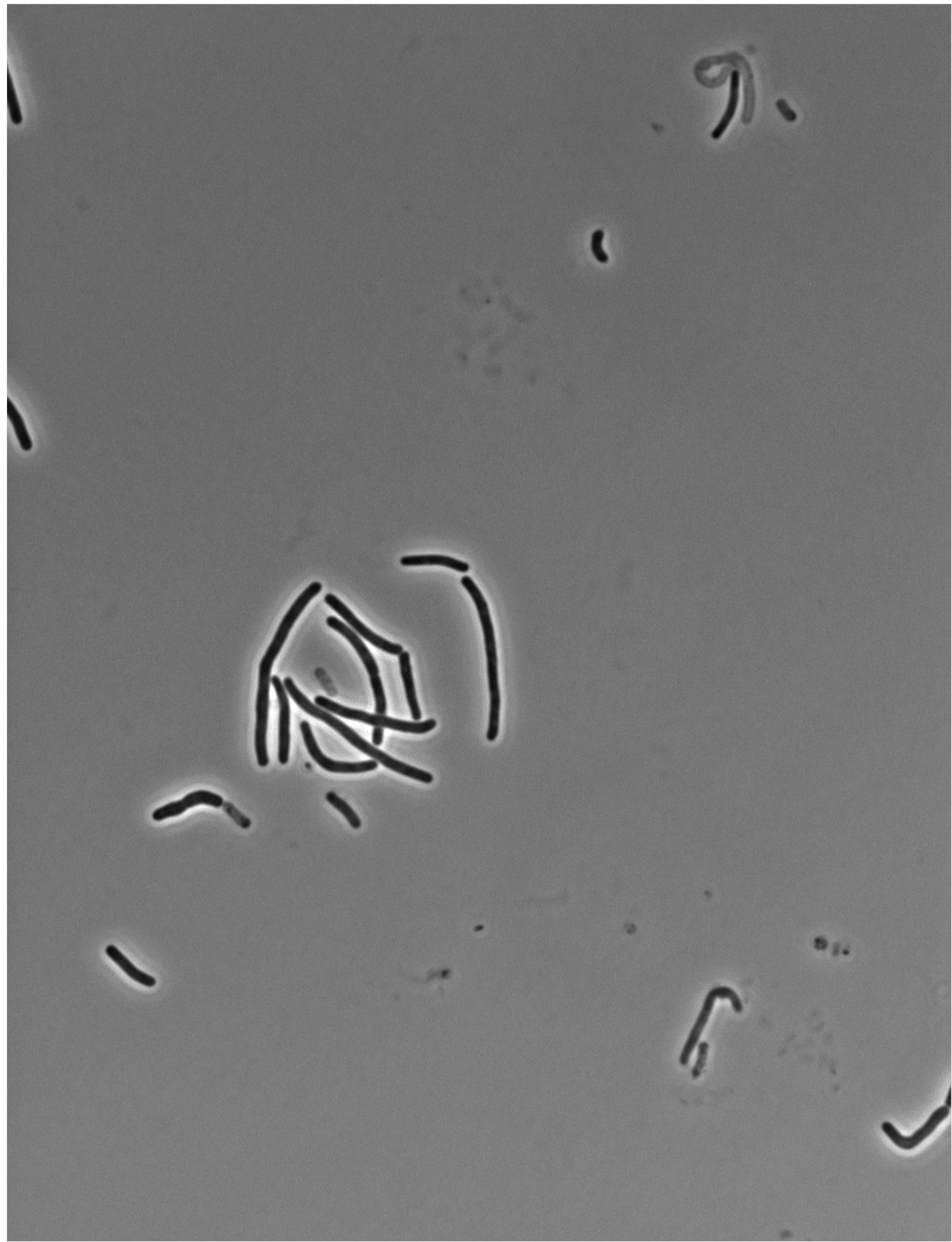

Supplement: Supplementary file 7 — Source data Fig. 2 [file 44319_2025_547_MOESM7_ESM.zip › Figure 2/Fig. 2B/delta pona delta ragb and Roda.tiff]

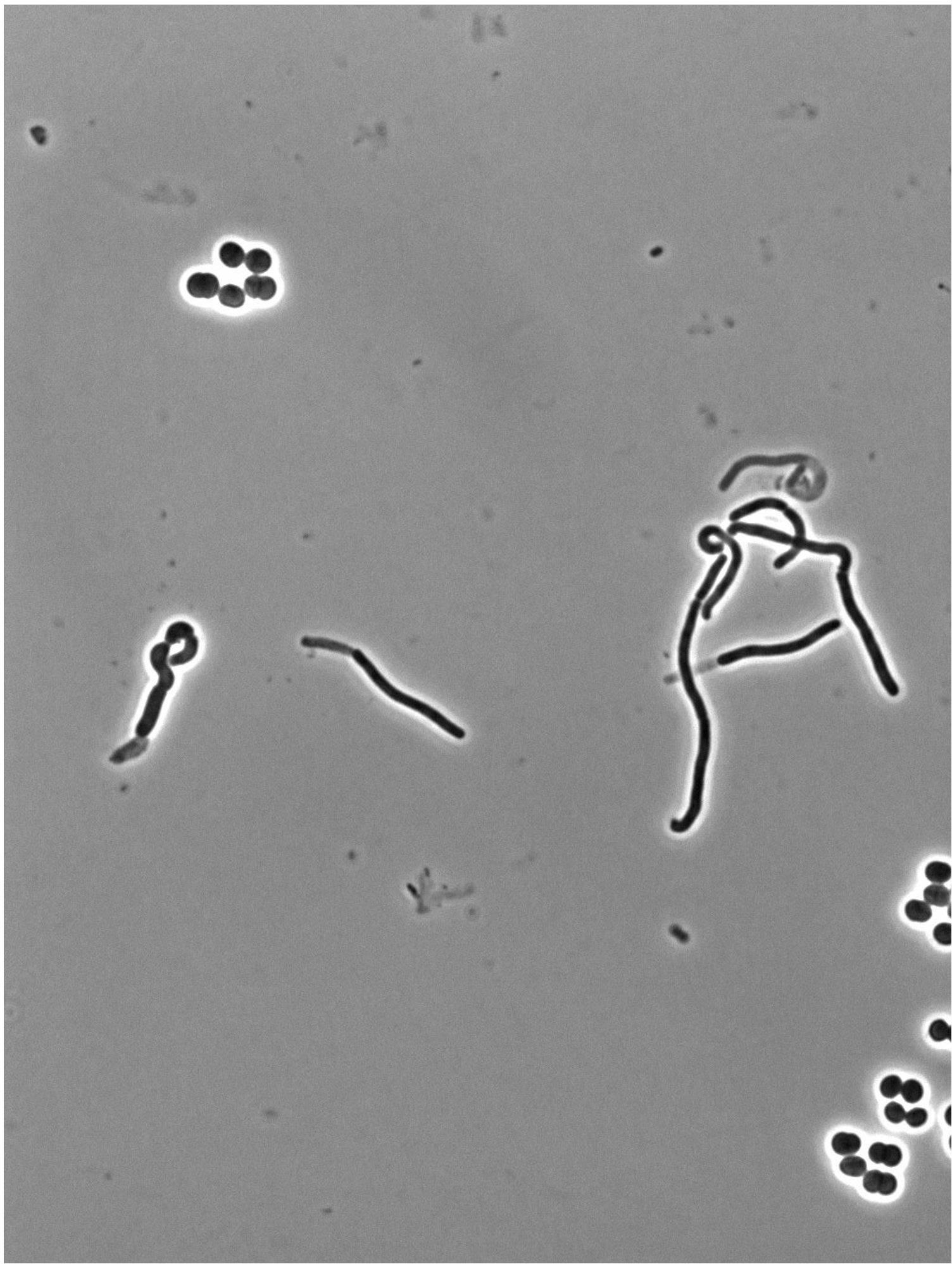

Supplement: Supplementary file 7 — Source data Fig. 2 [file 44319_2025_547_MOESM7_ESM.zip › Figure 2/Fig. 2B/delta pona delta ragB rodA D280A.tiff]

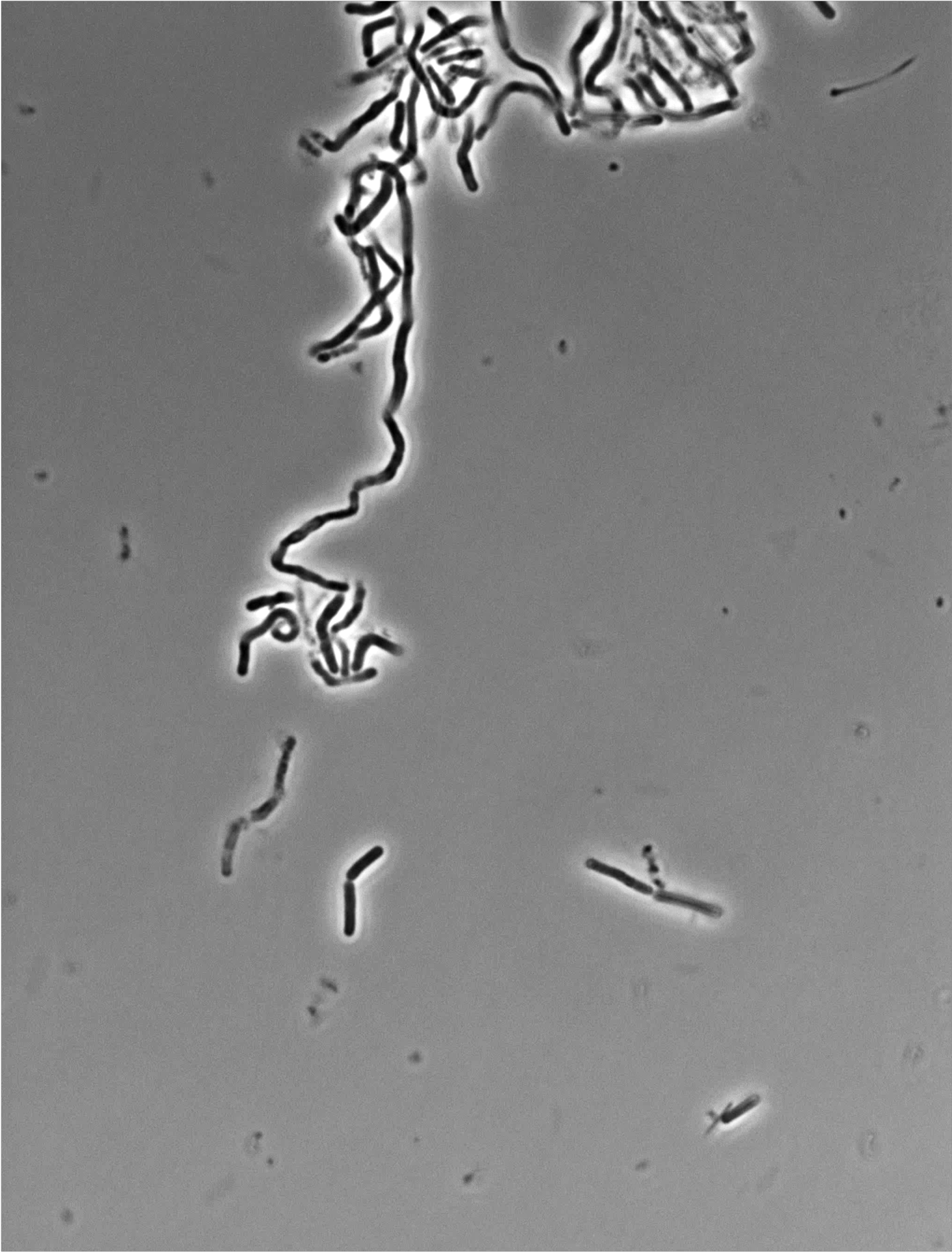

Supplement: Supplementary file 7 — Source data Fig. 2 [file 44319_2025_547_MOESM7_ESM.zip › Figure 2/Fig. 2B/delta pona delta ragB.tiff]

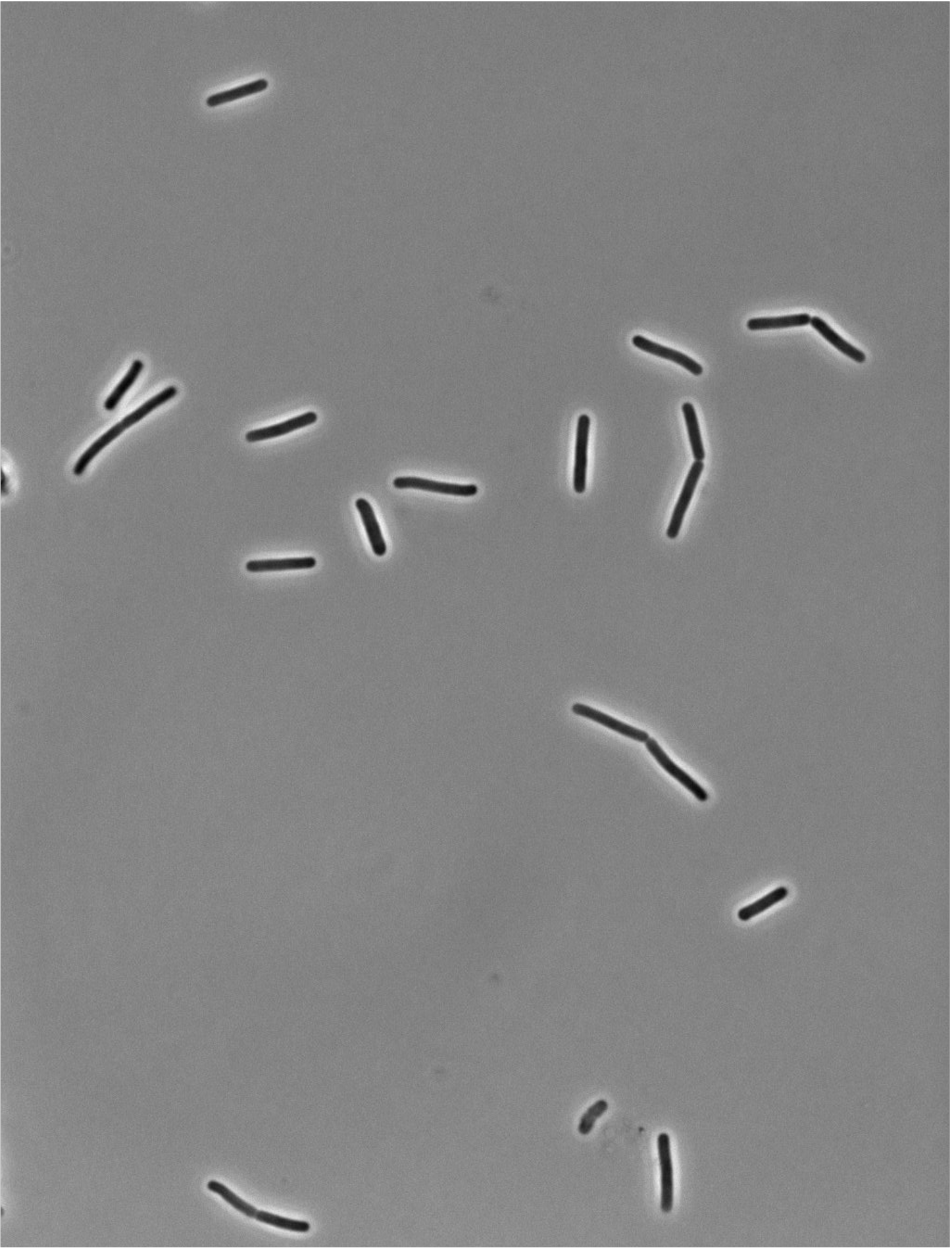

Supplement: Supplementary file 7 — Source data Fig. 2 [file 44319_2025_547_MOESM7_ESM.zip › Figure 2/Fig. 2B/deltaponA delta ragB and RagB.tiff]

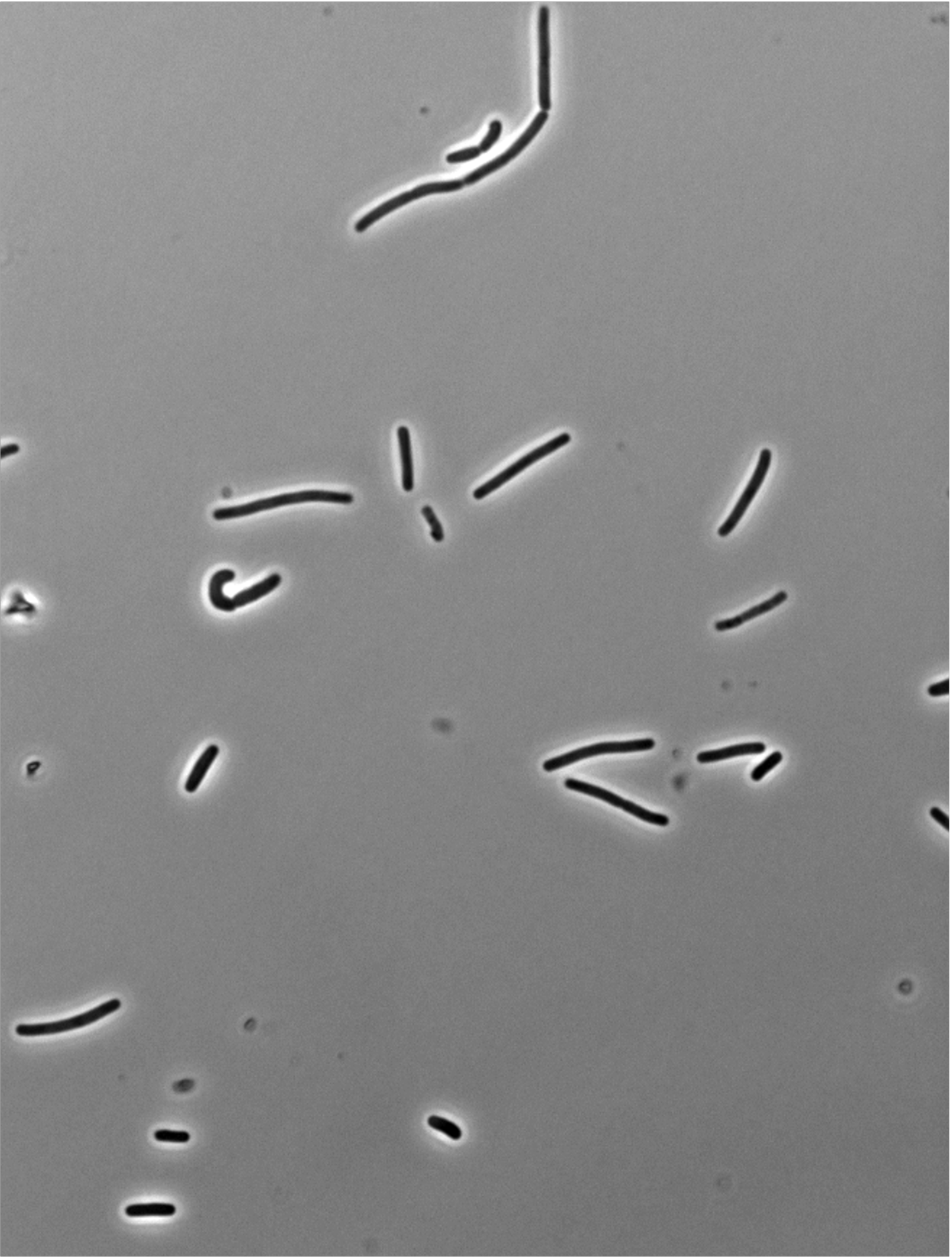

Supplement: Supplementary file 7 — Source data Fig. 2 [file 44319_2025_547_MOESM7_ESM.zip › Figure 2/Fig. 2B/dlta pona.tiff]

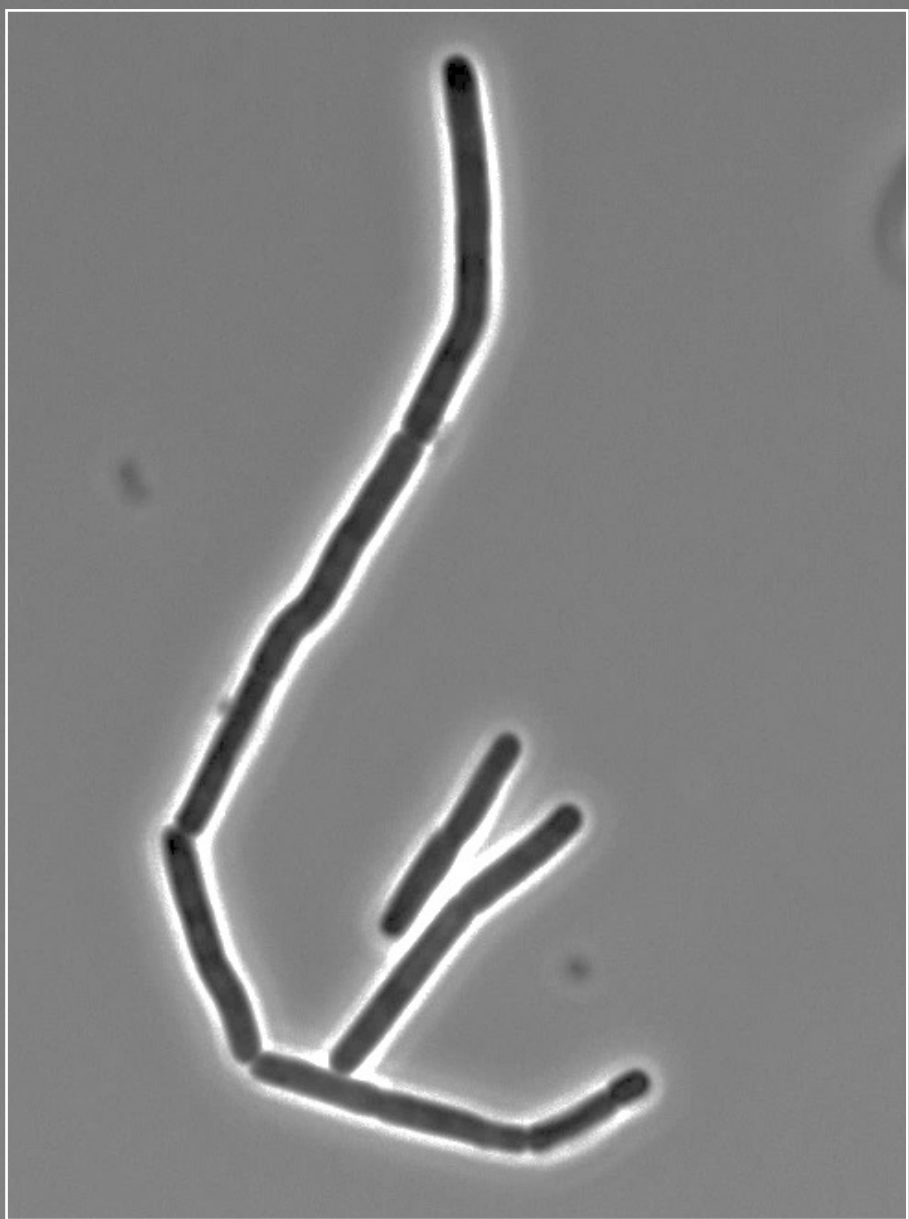

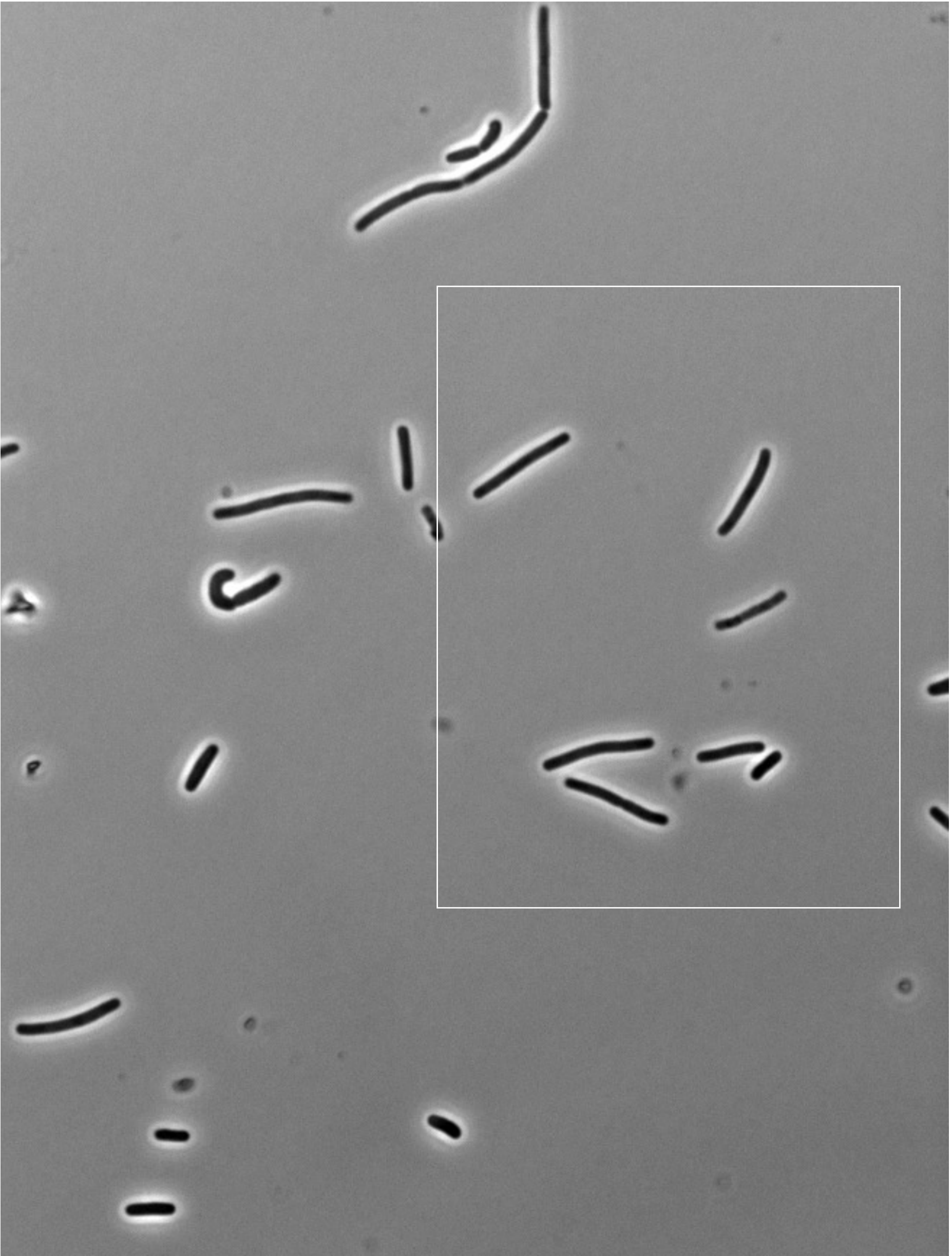

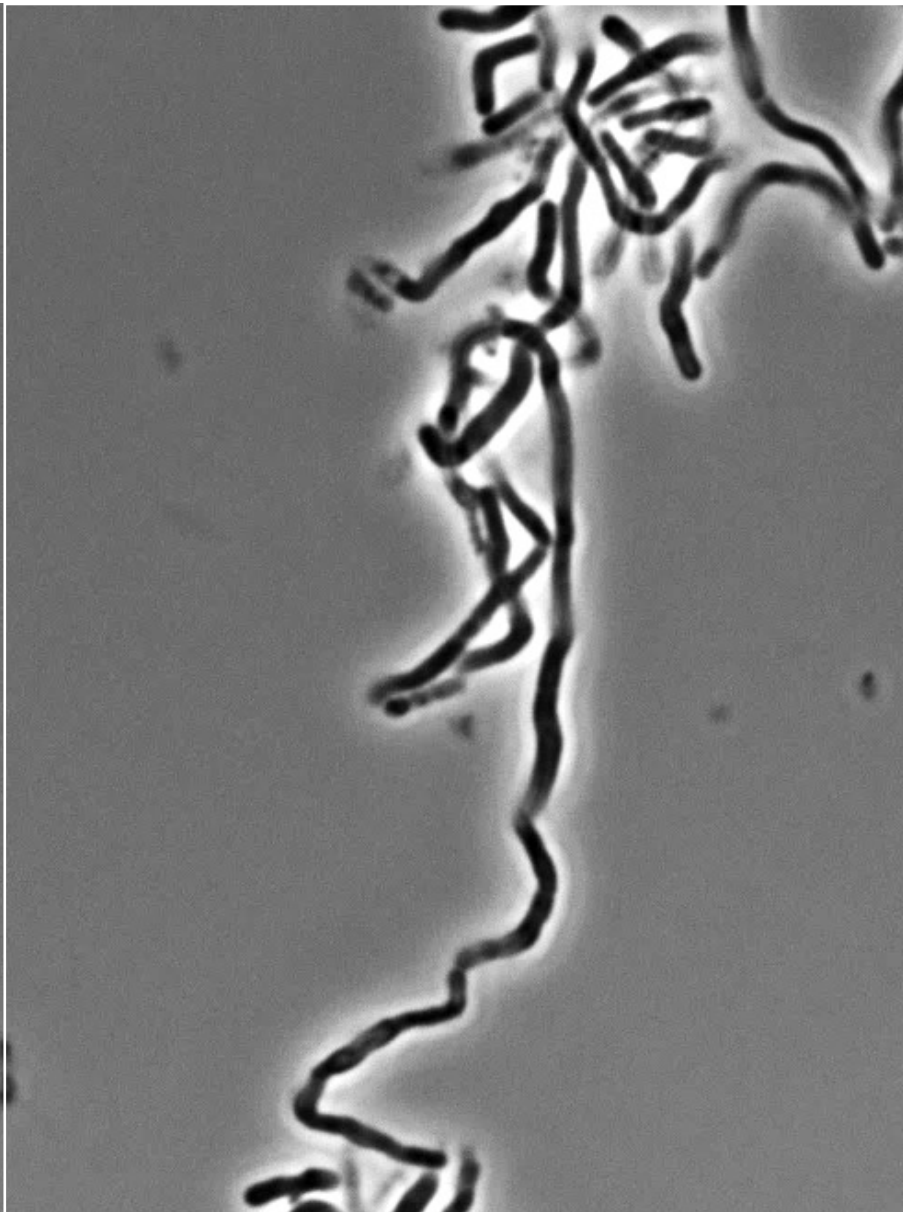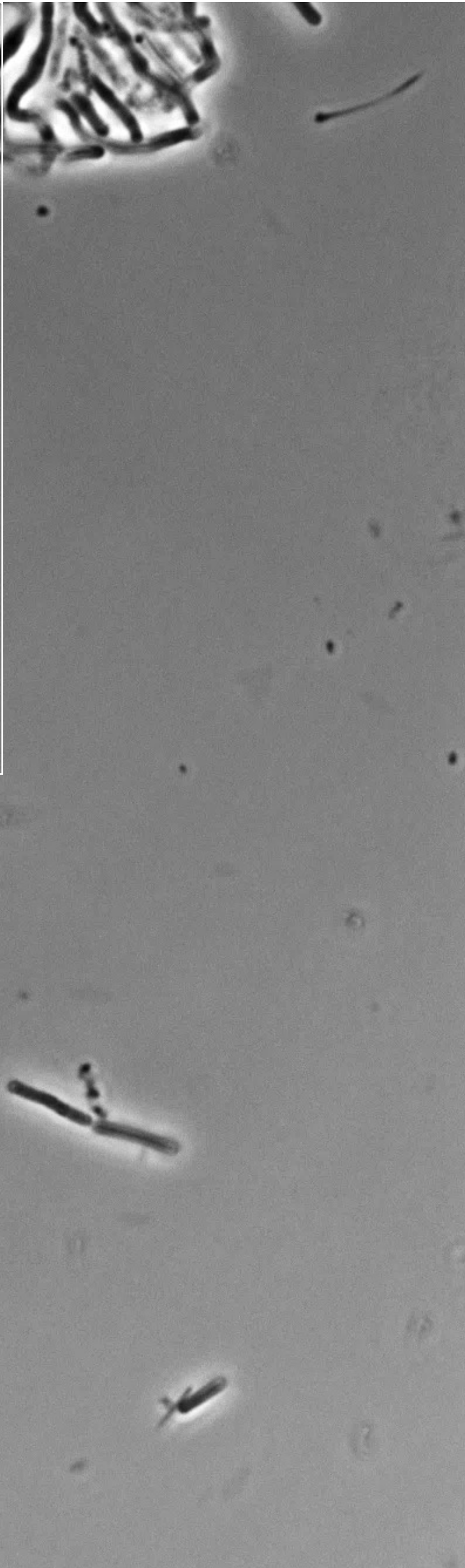

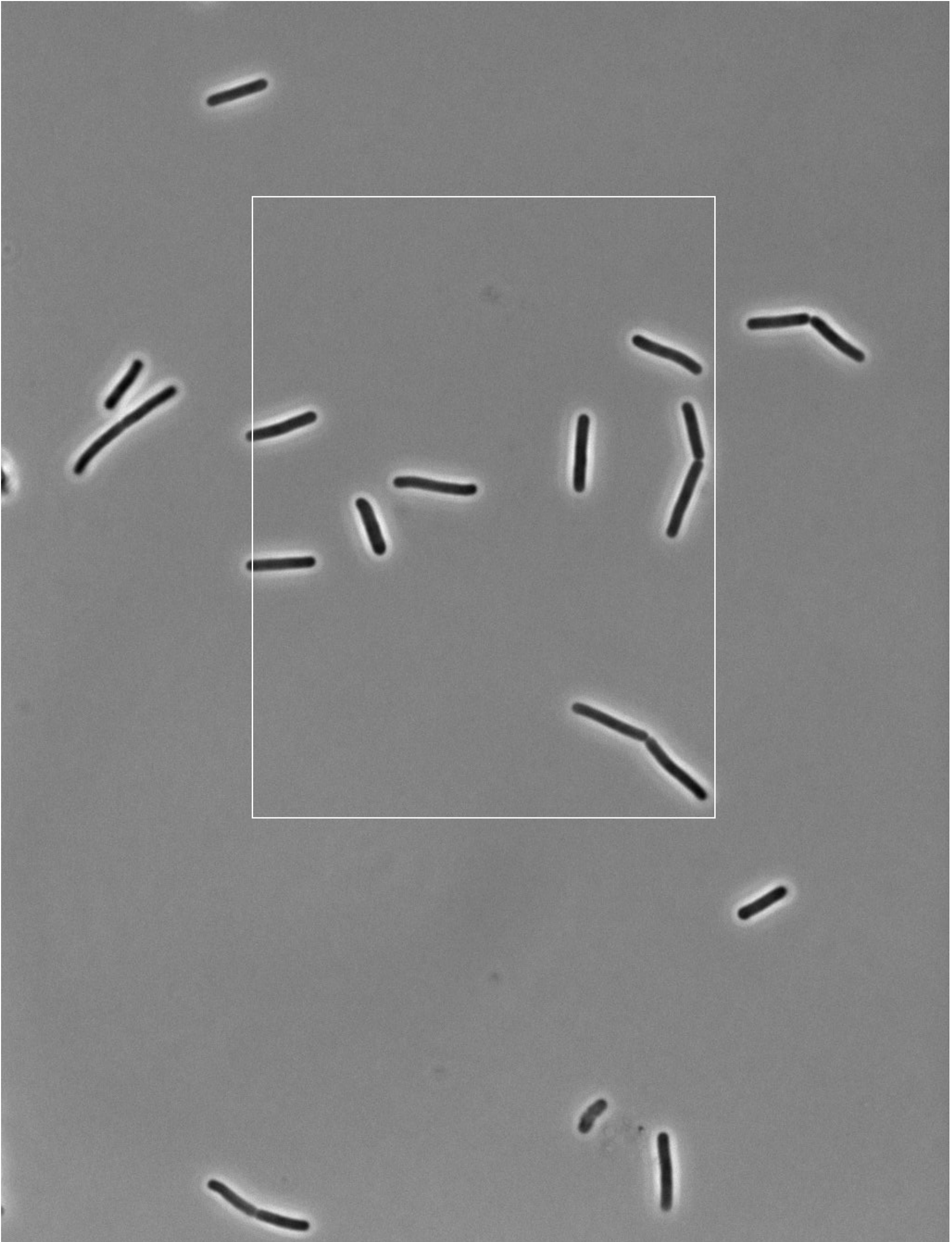

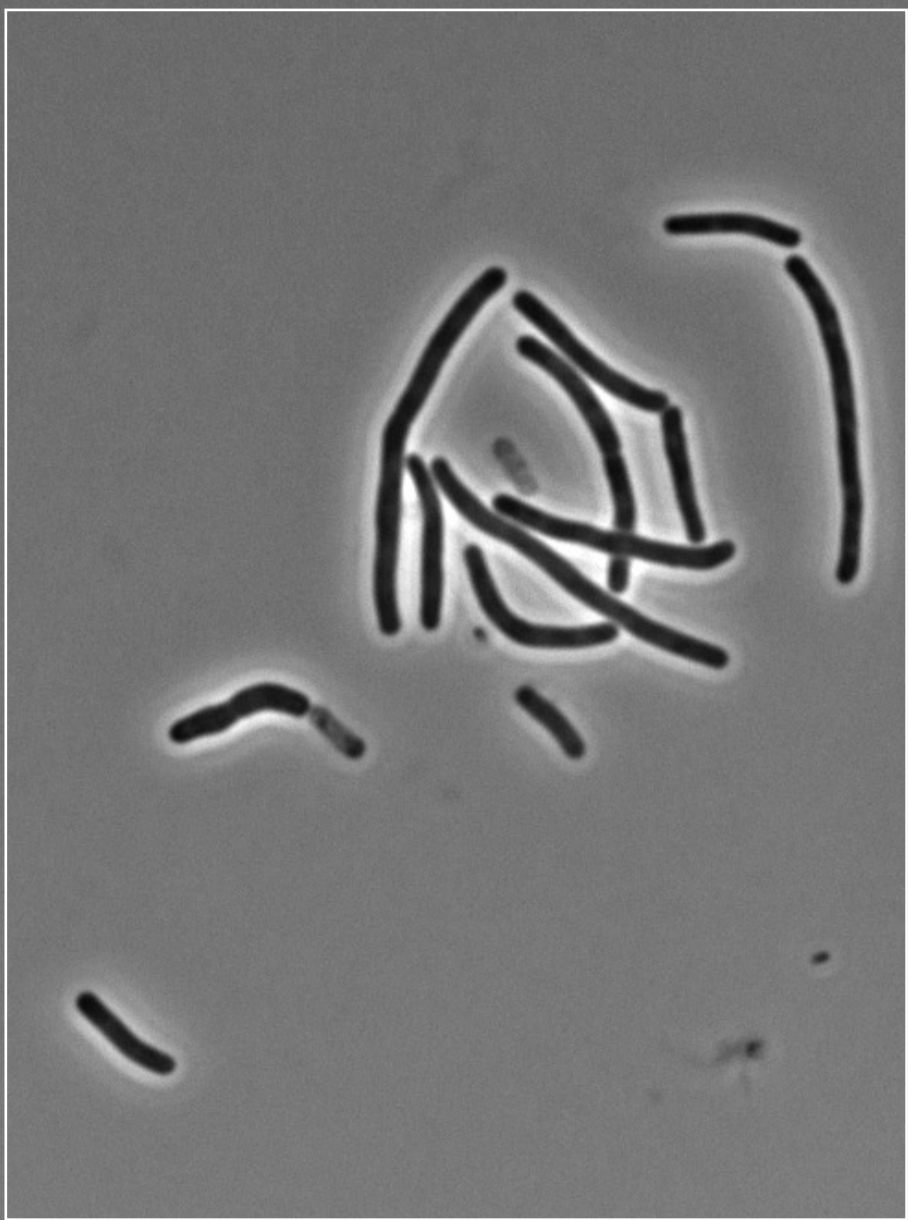

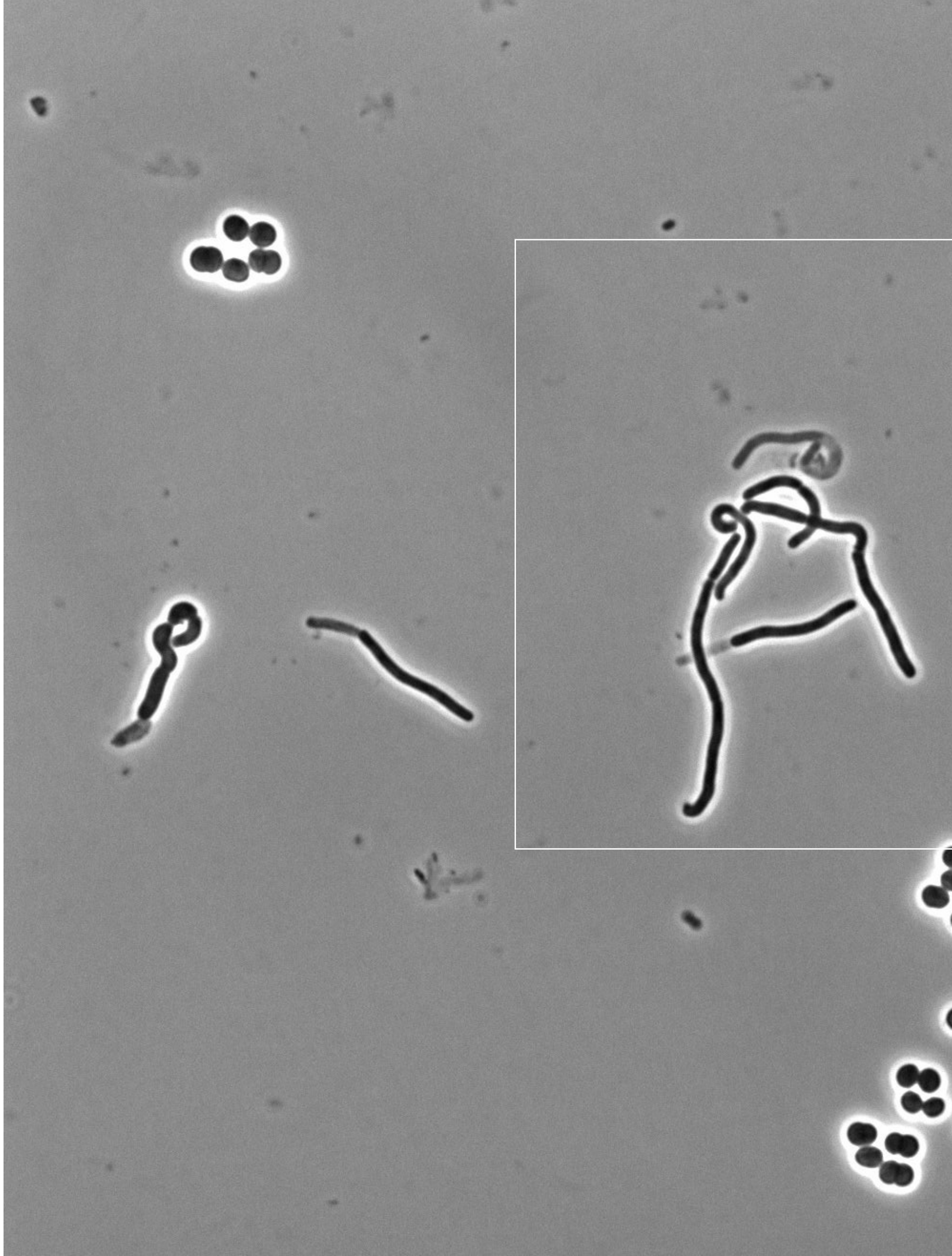

Supplement: Supplementary file 7 — Source data Fig. 2 [file 44319_2025_547_MOESM7_ESM.zip › Figure 2/Fig. 2B/Fig.2B.pdf]

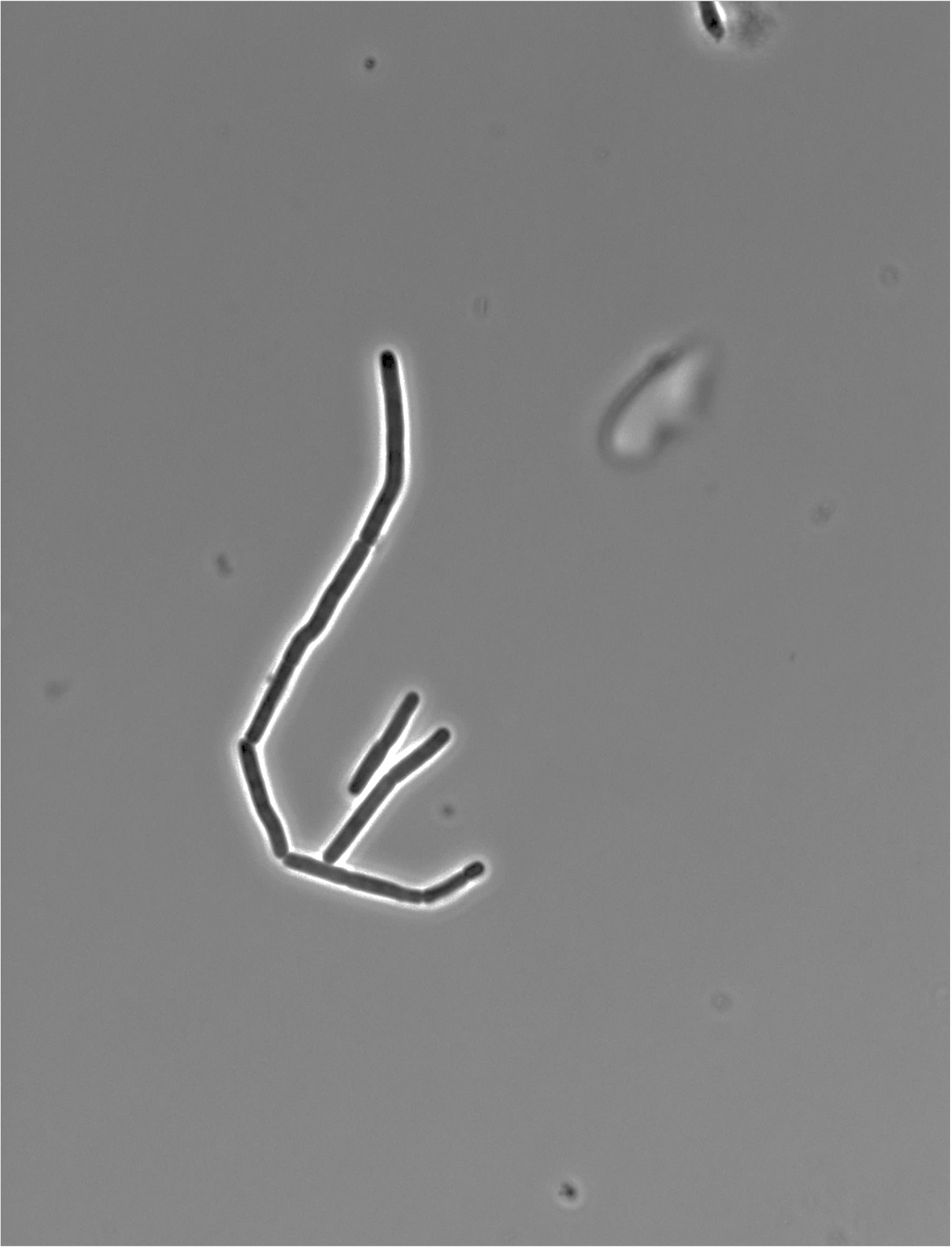

Supplement: Supplementary file 7 — Source data Fig. 2 [file 44319_2025_547_MOESM7_ESM.zip › Figure 2/Fig. 2B/WT.tiff]

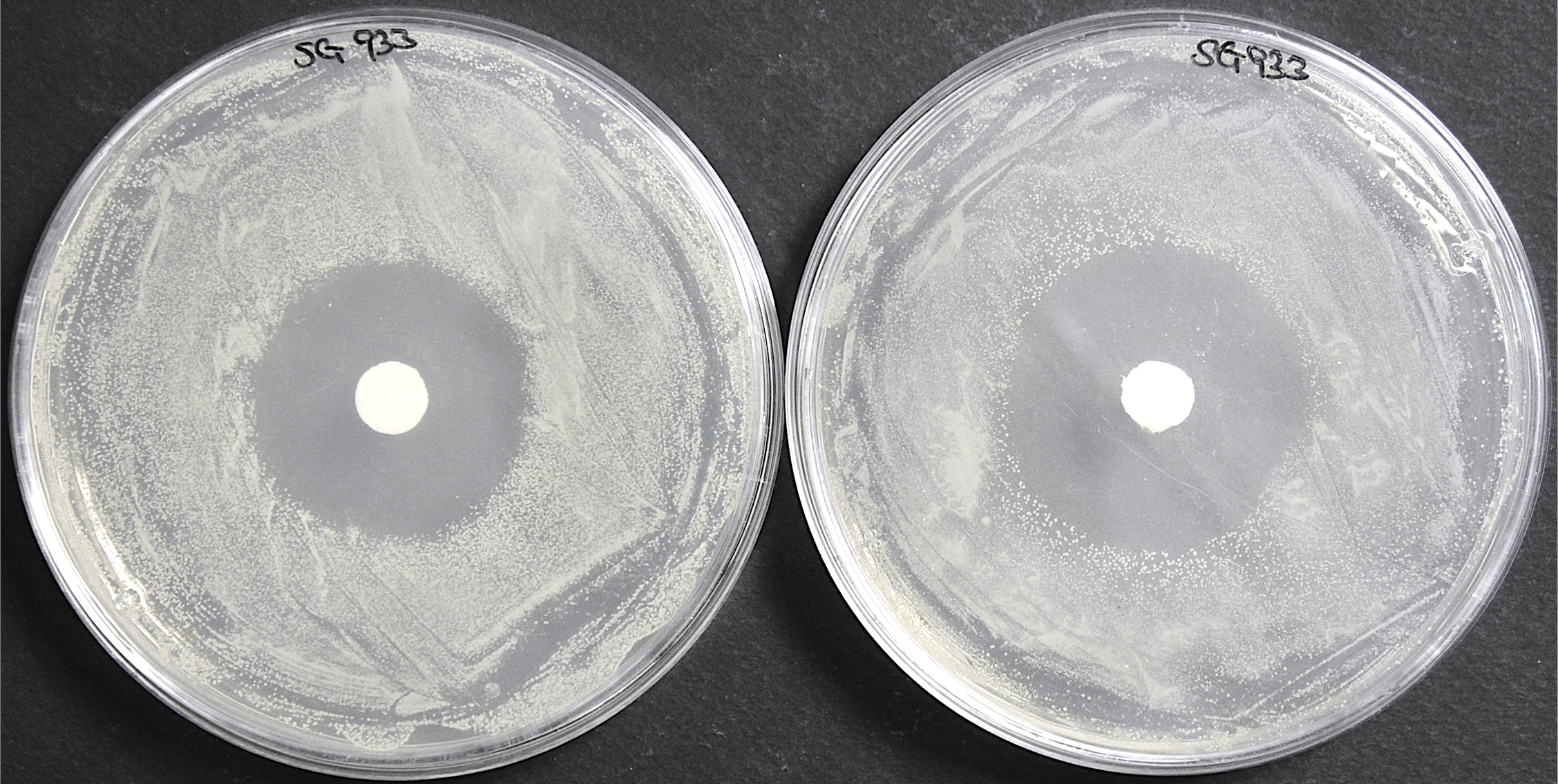

Supplement: Supplementary file 7 — Source data Fig. 2 [file 44319_2025_547_MOESM7_ESM.zip › Figure 2/Fig. 2C/2 plates.tiff]

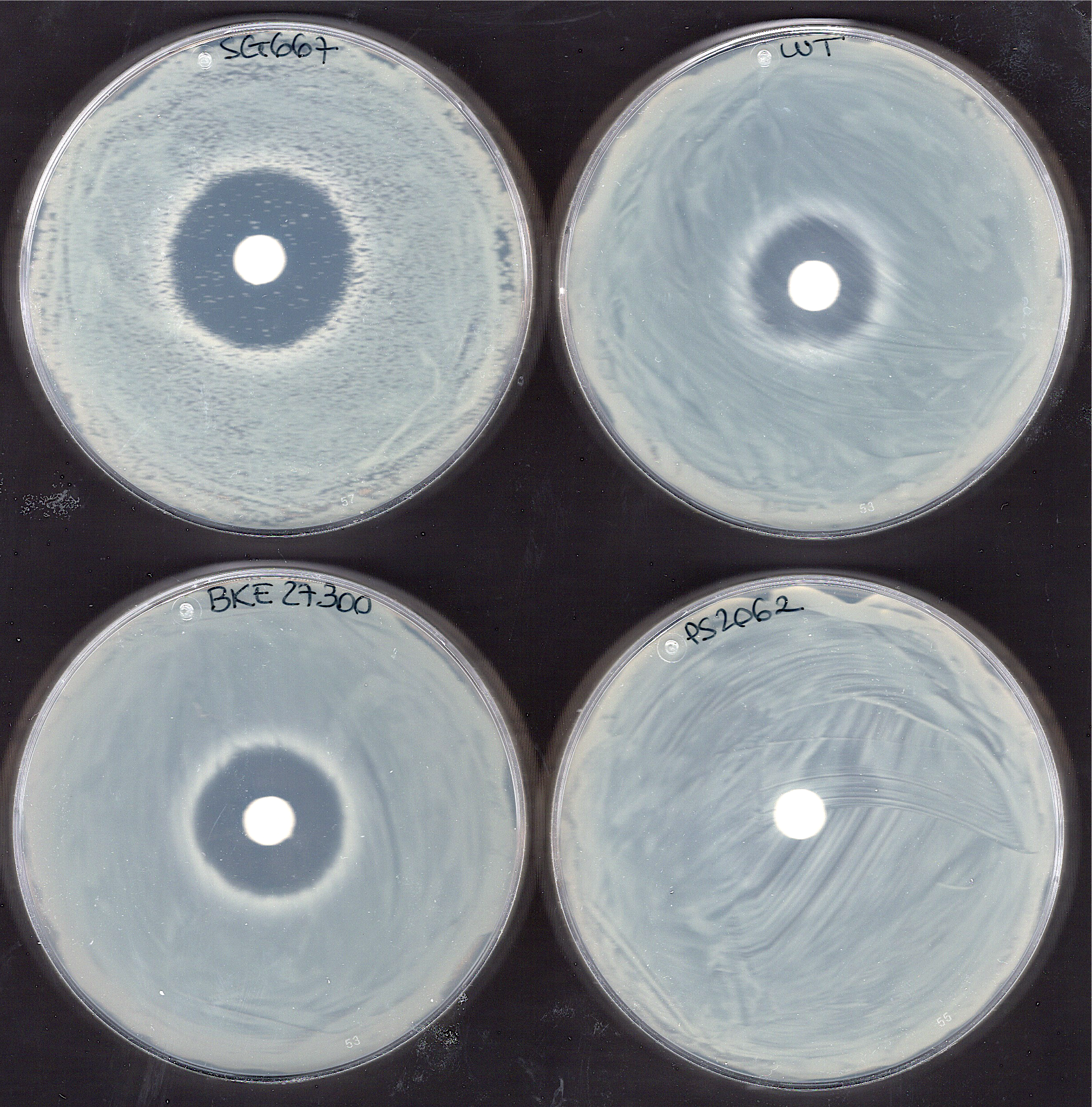

Supplement: Supplementary file 7 — Source data Fig. 2 [file 44319_2025_547_MOESM7_ESM.zip › Figure 2/Fig. 2C/4 plates.tiff]

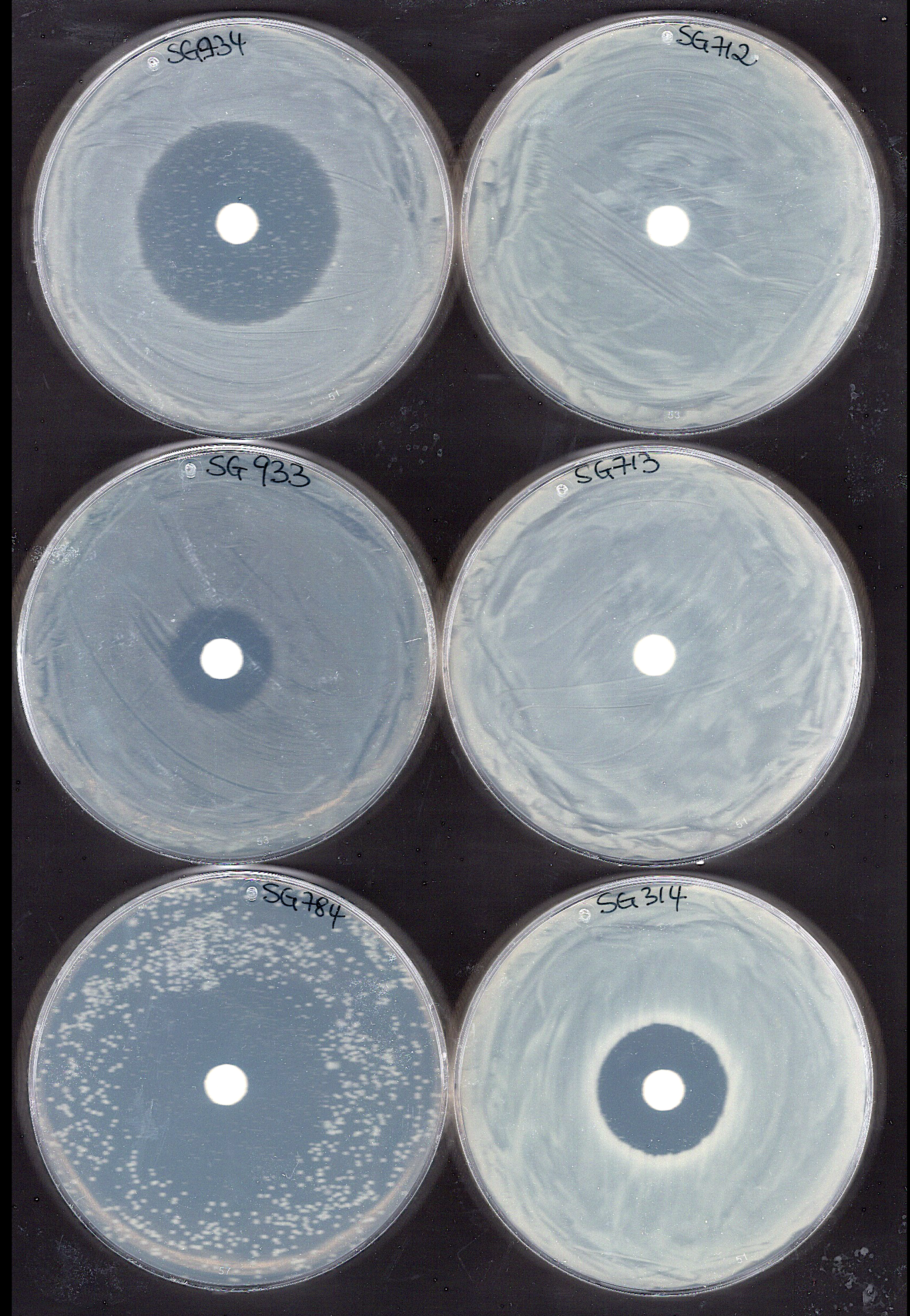

Supplement: Supplementary file 7 — Source data Fig. 2 [file 44319_2025_547_MOESM7_ESM.zip › Figure 2/Fig. 2C/6 plates.tiff]

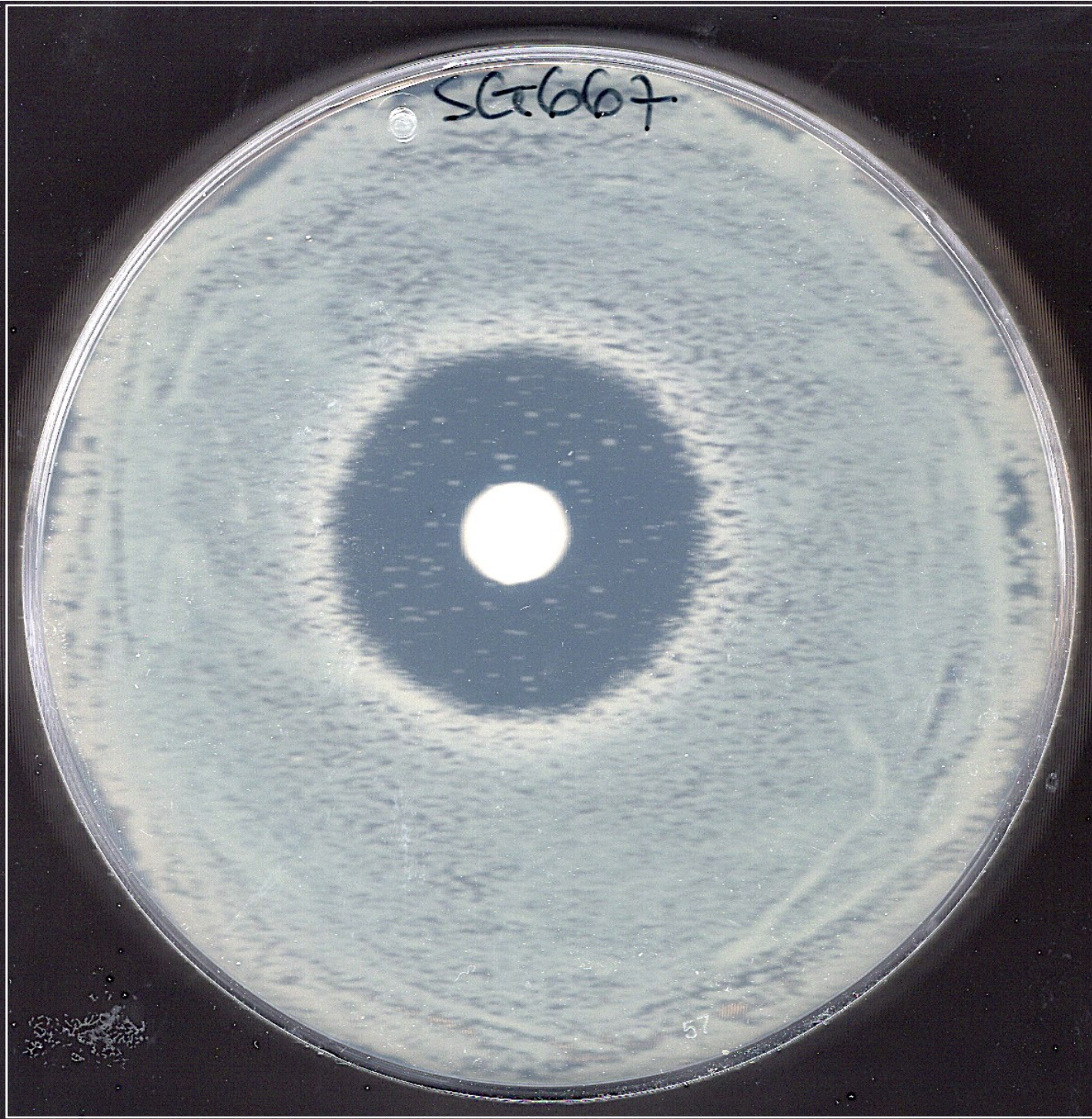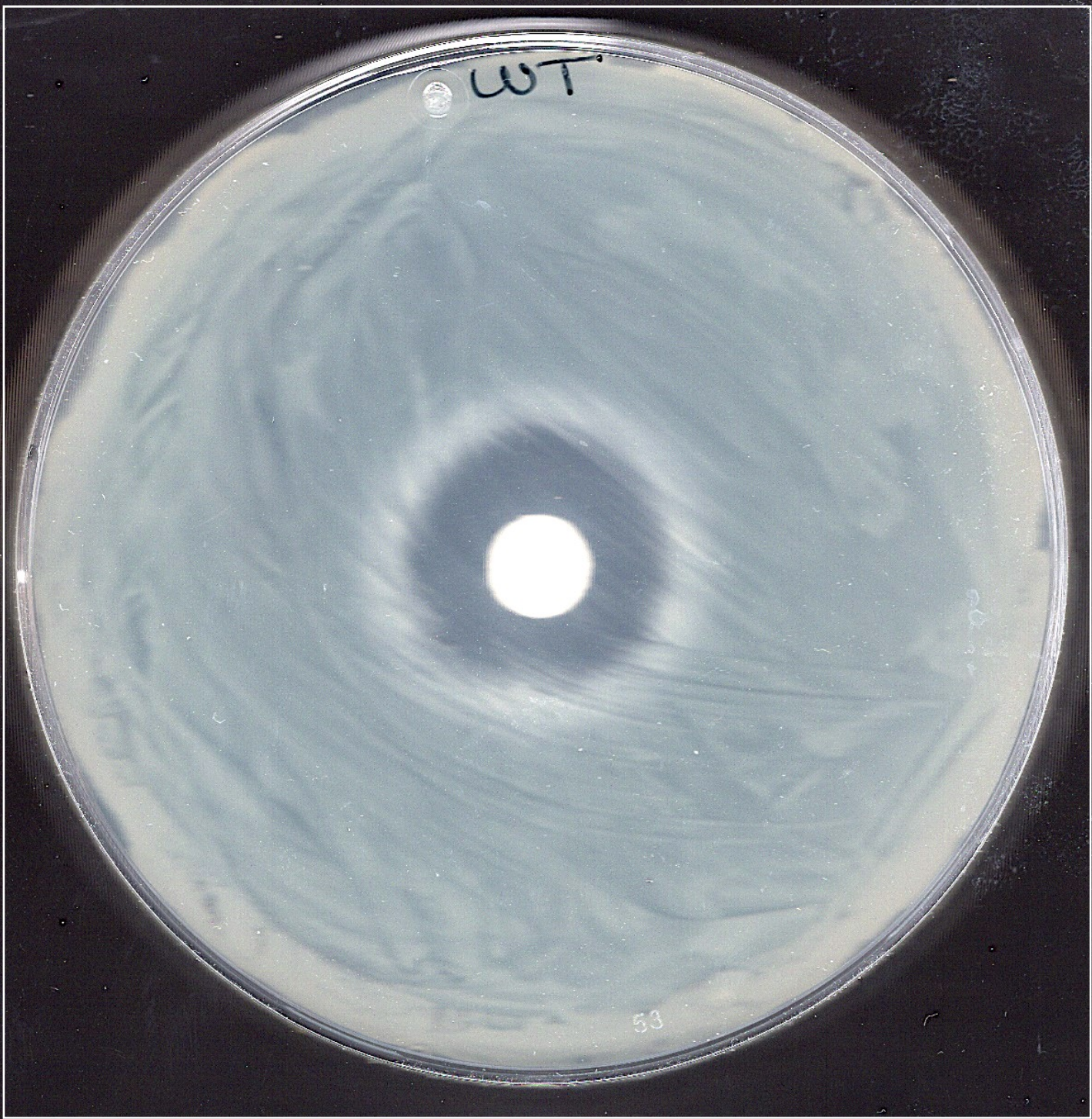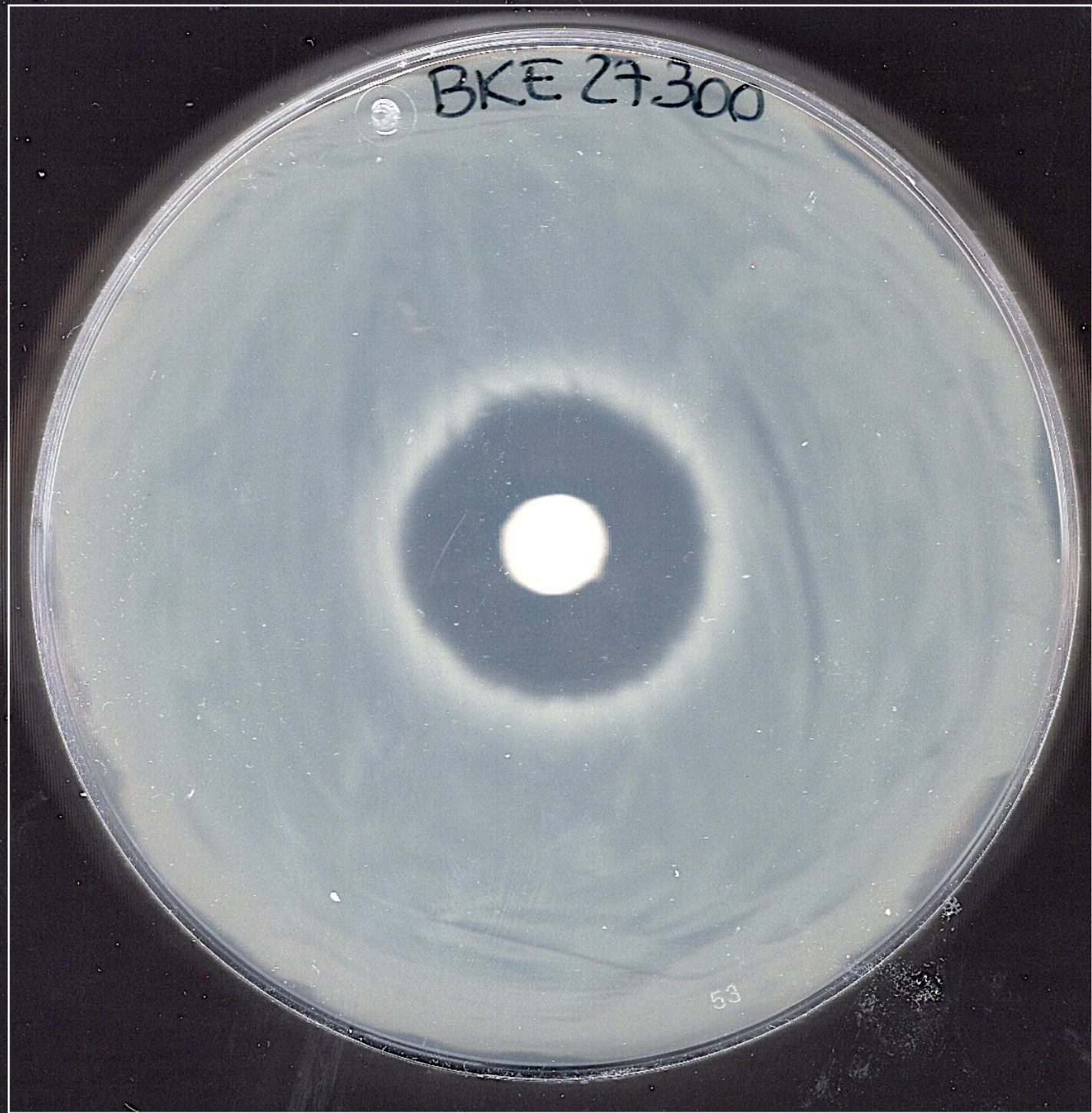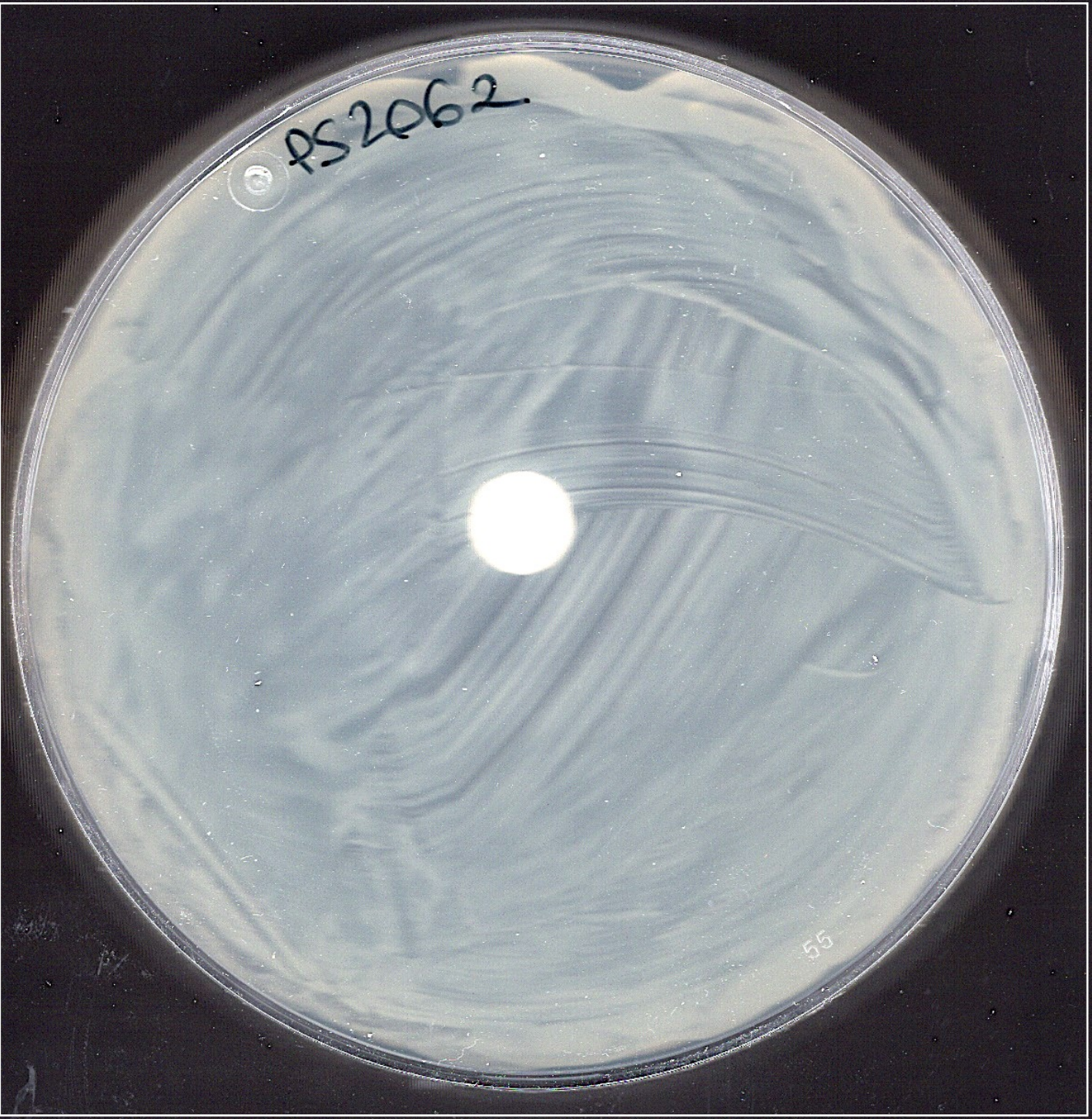

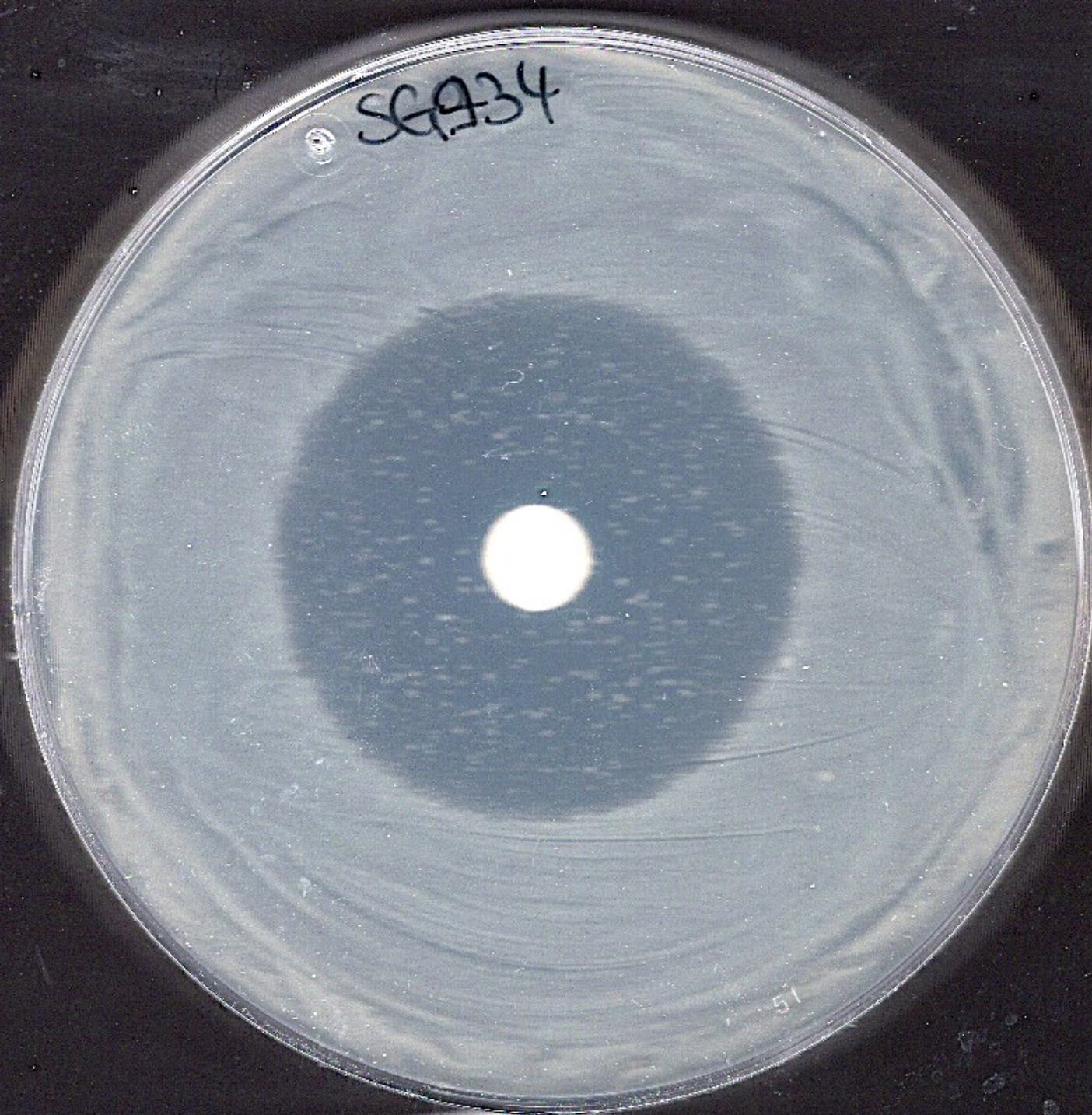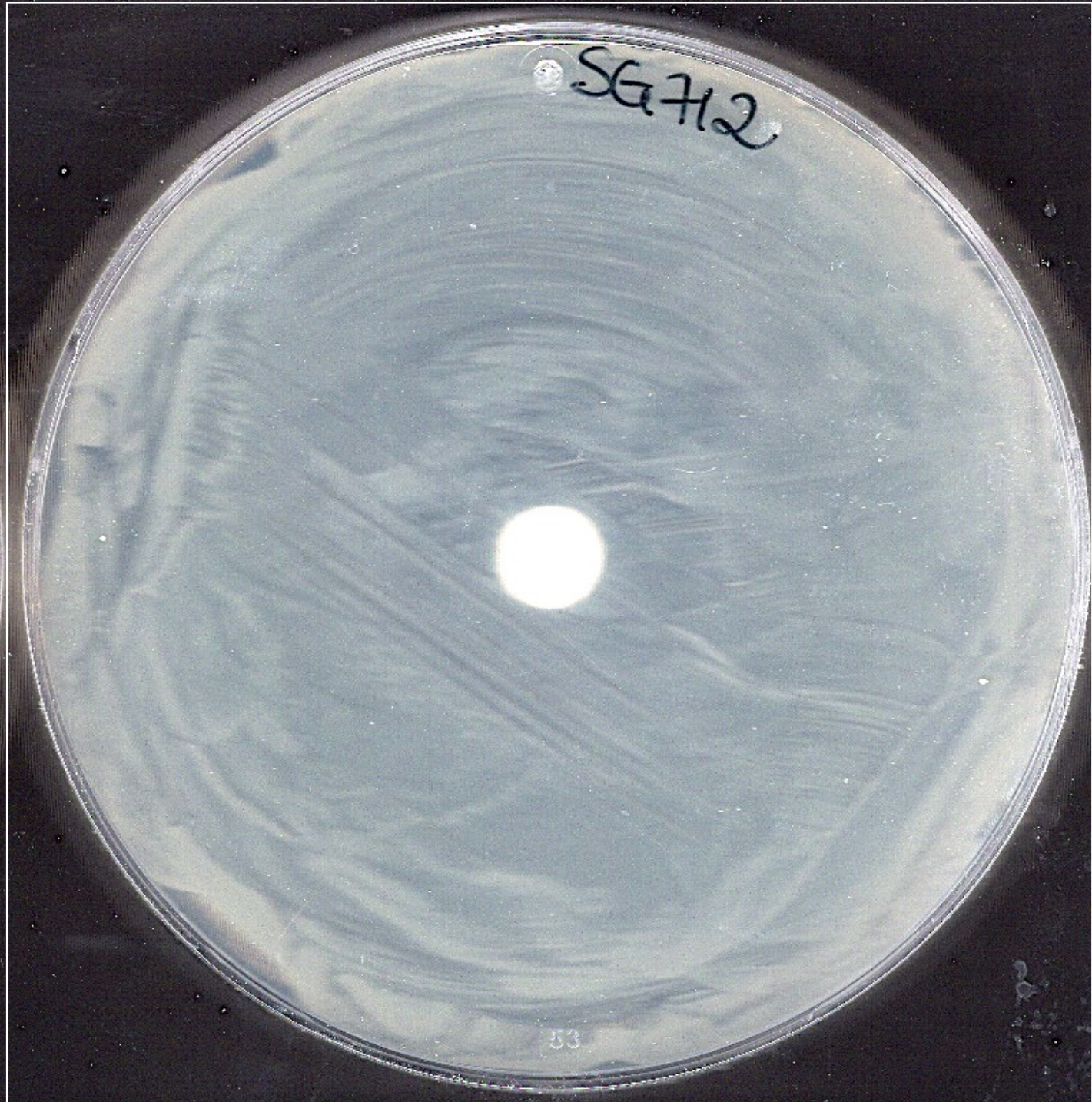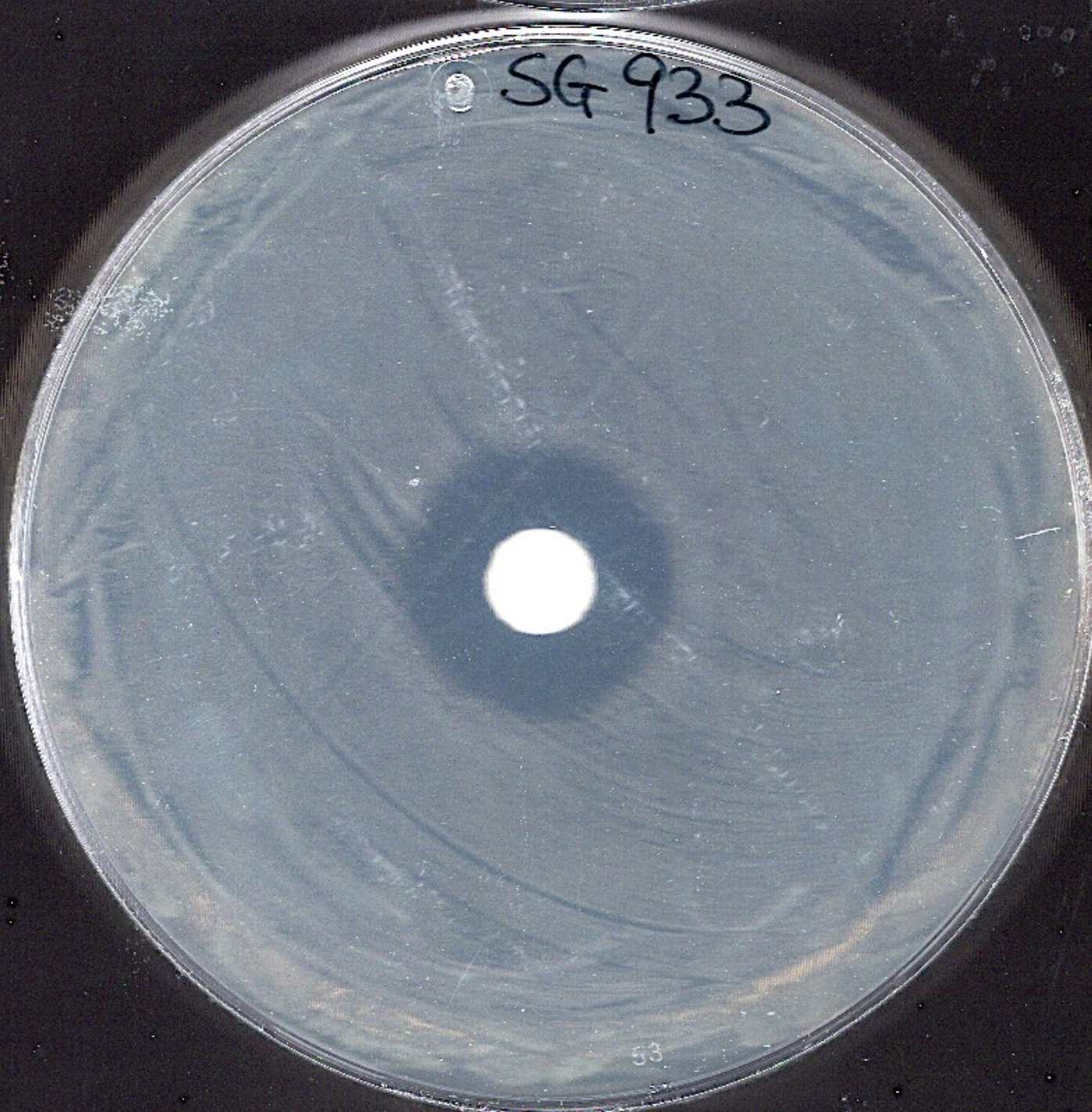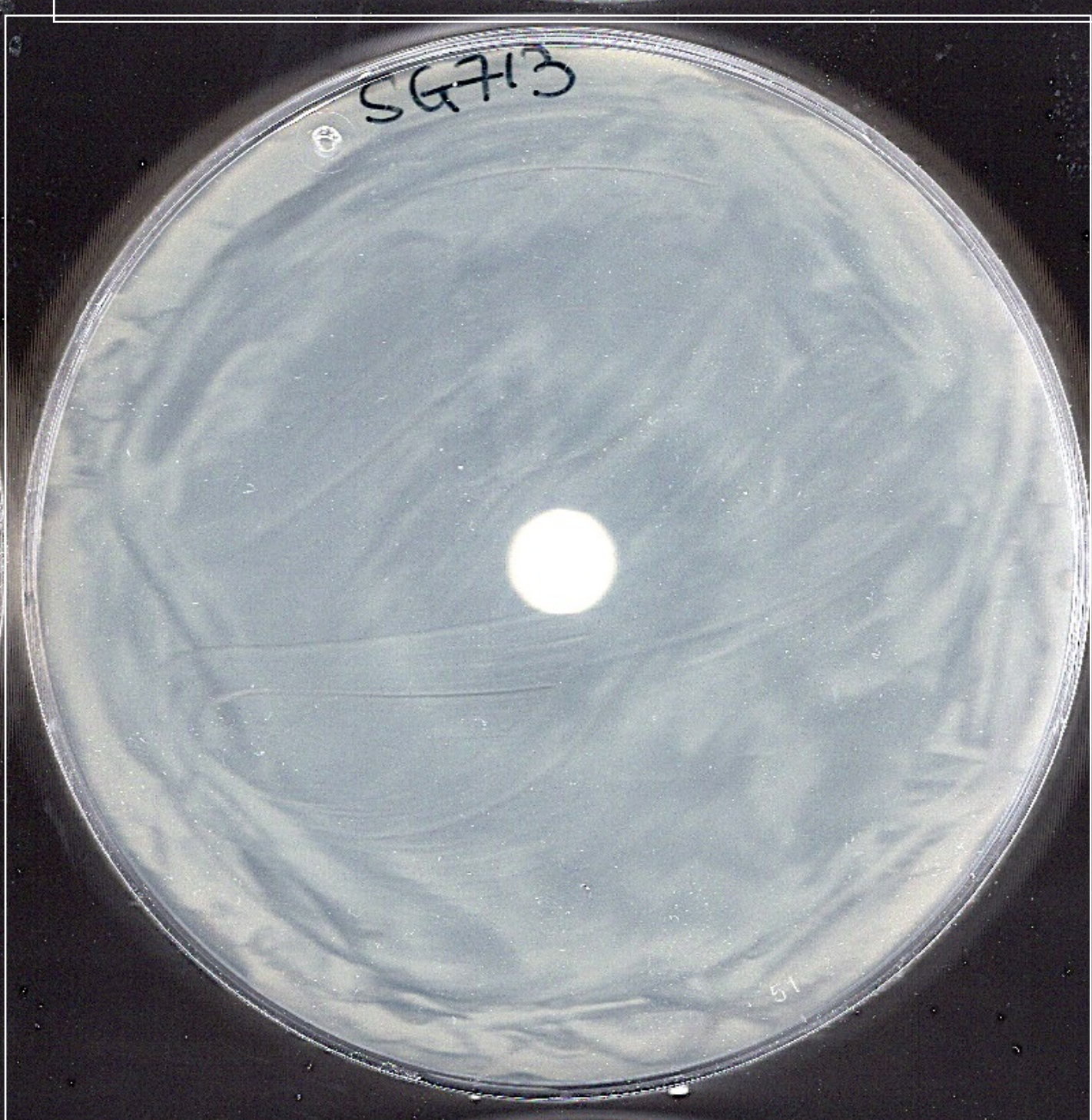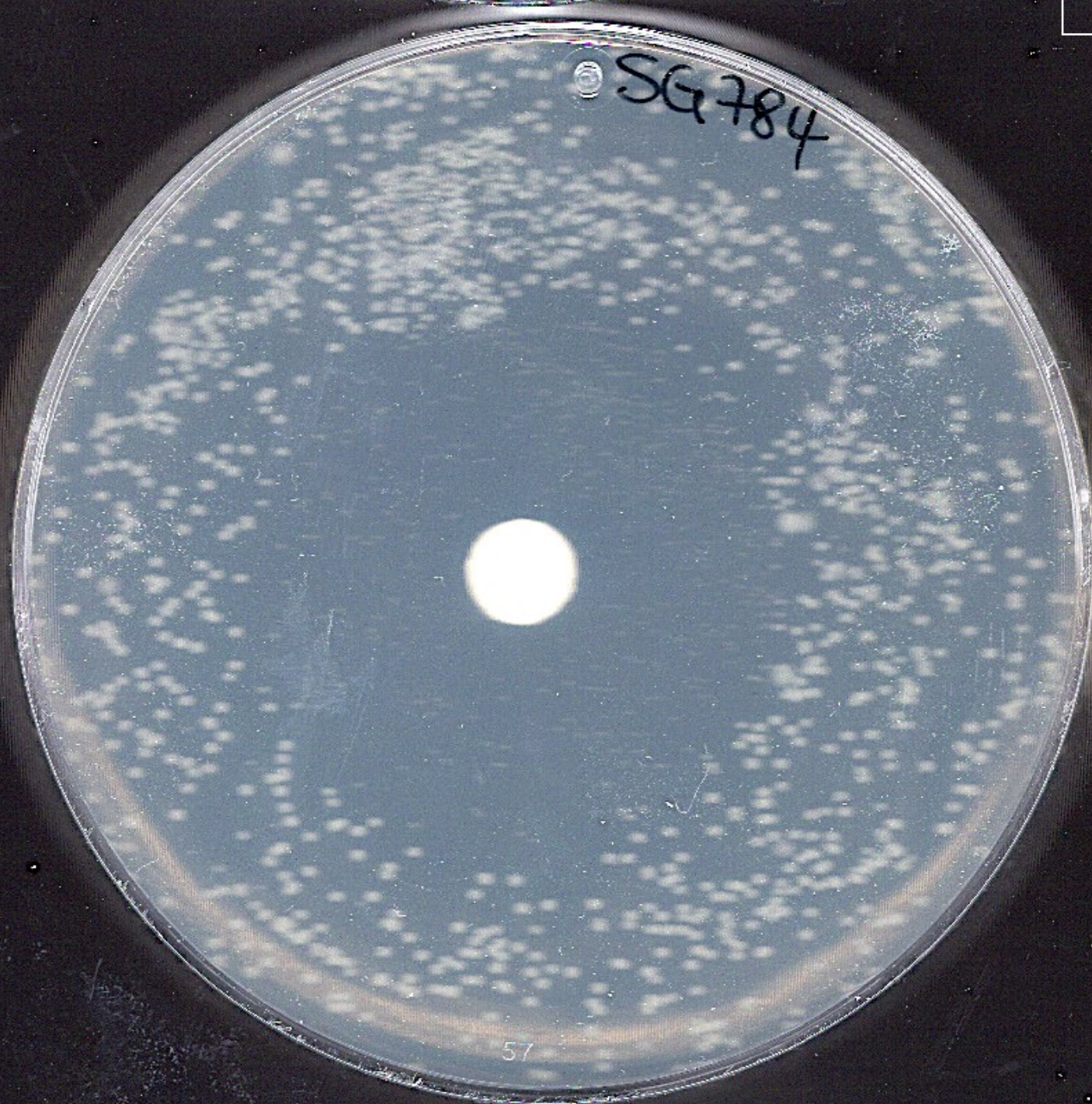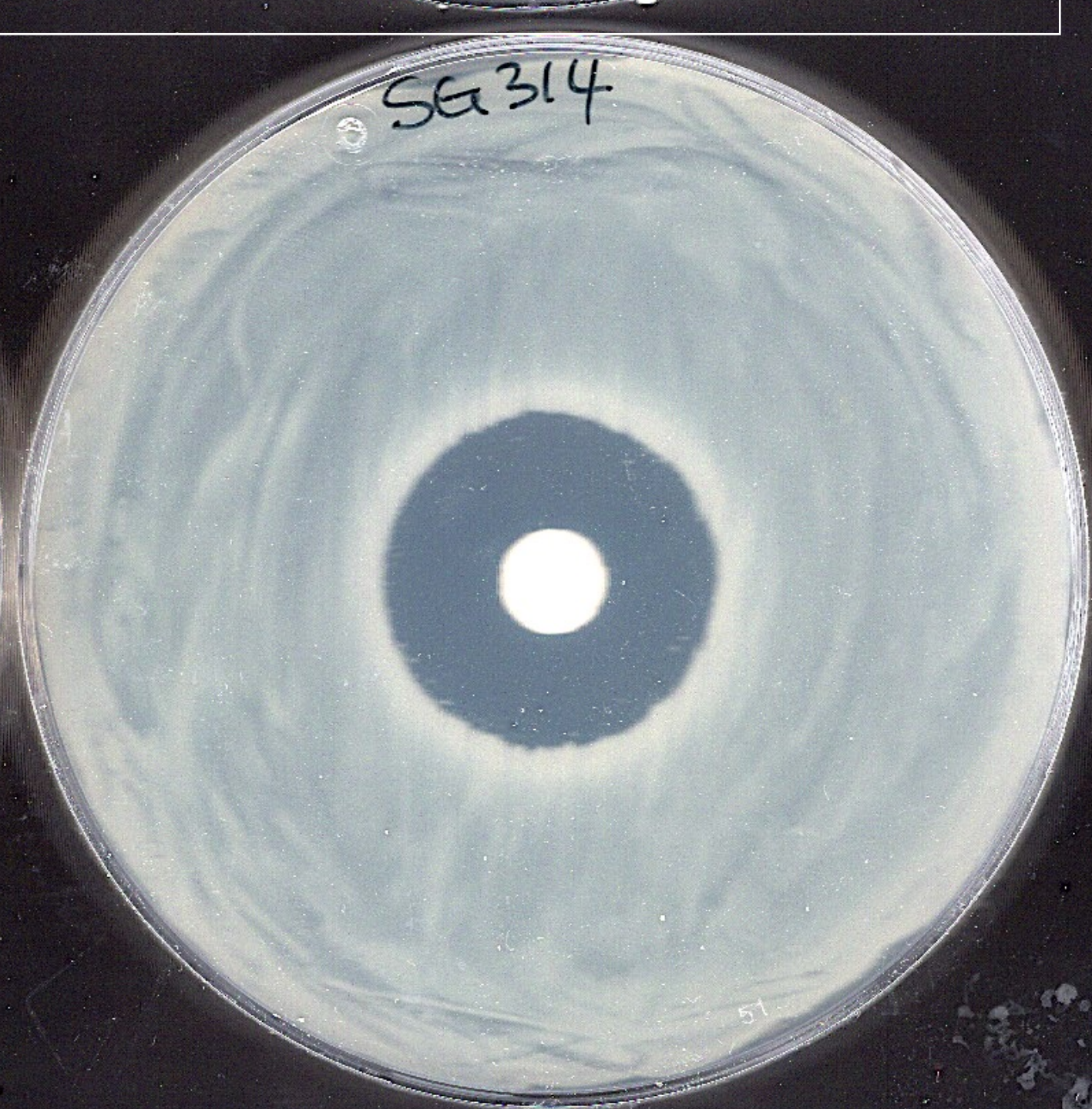

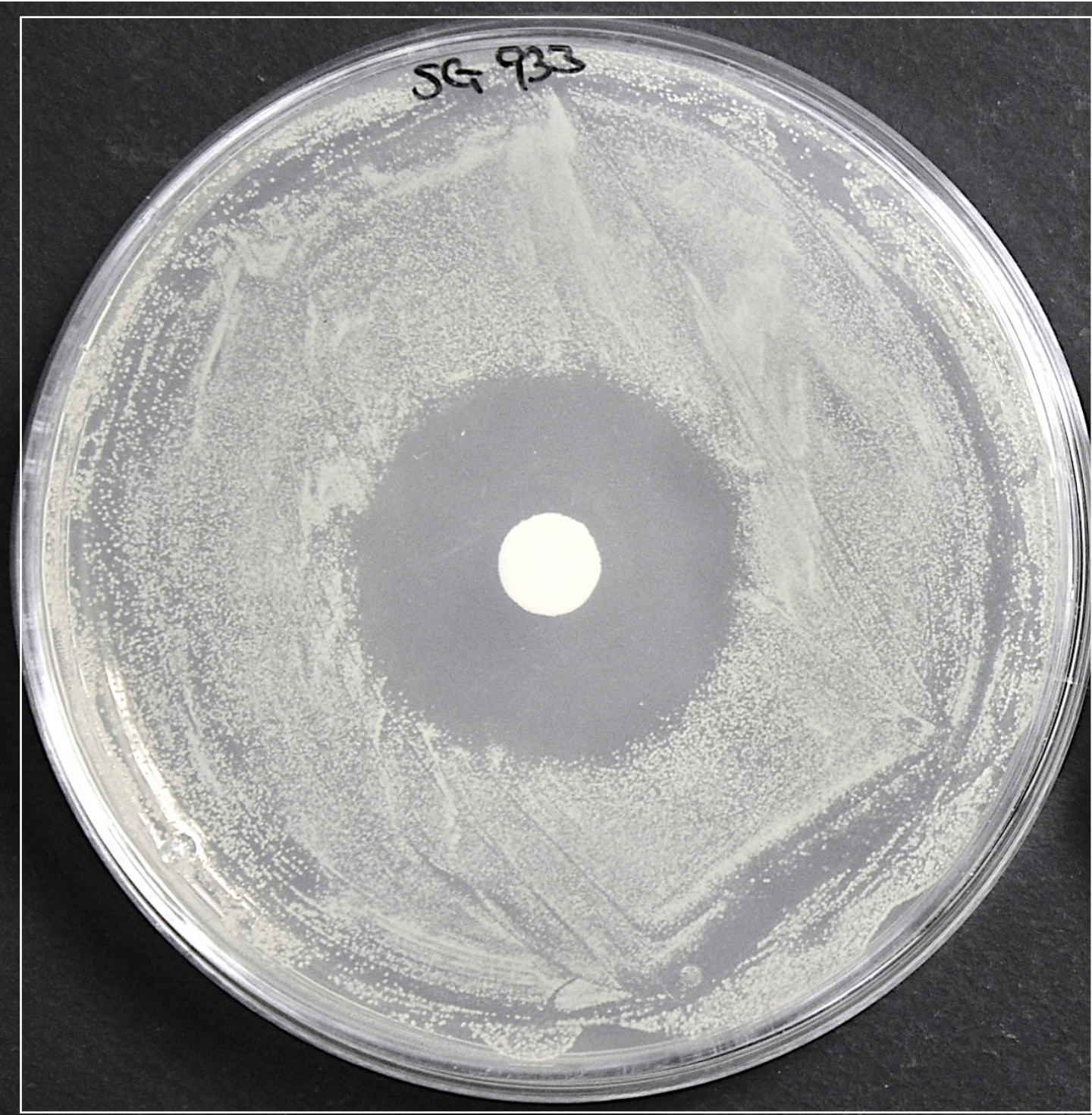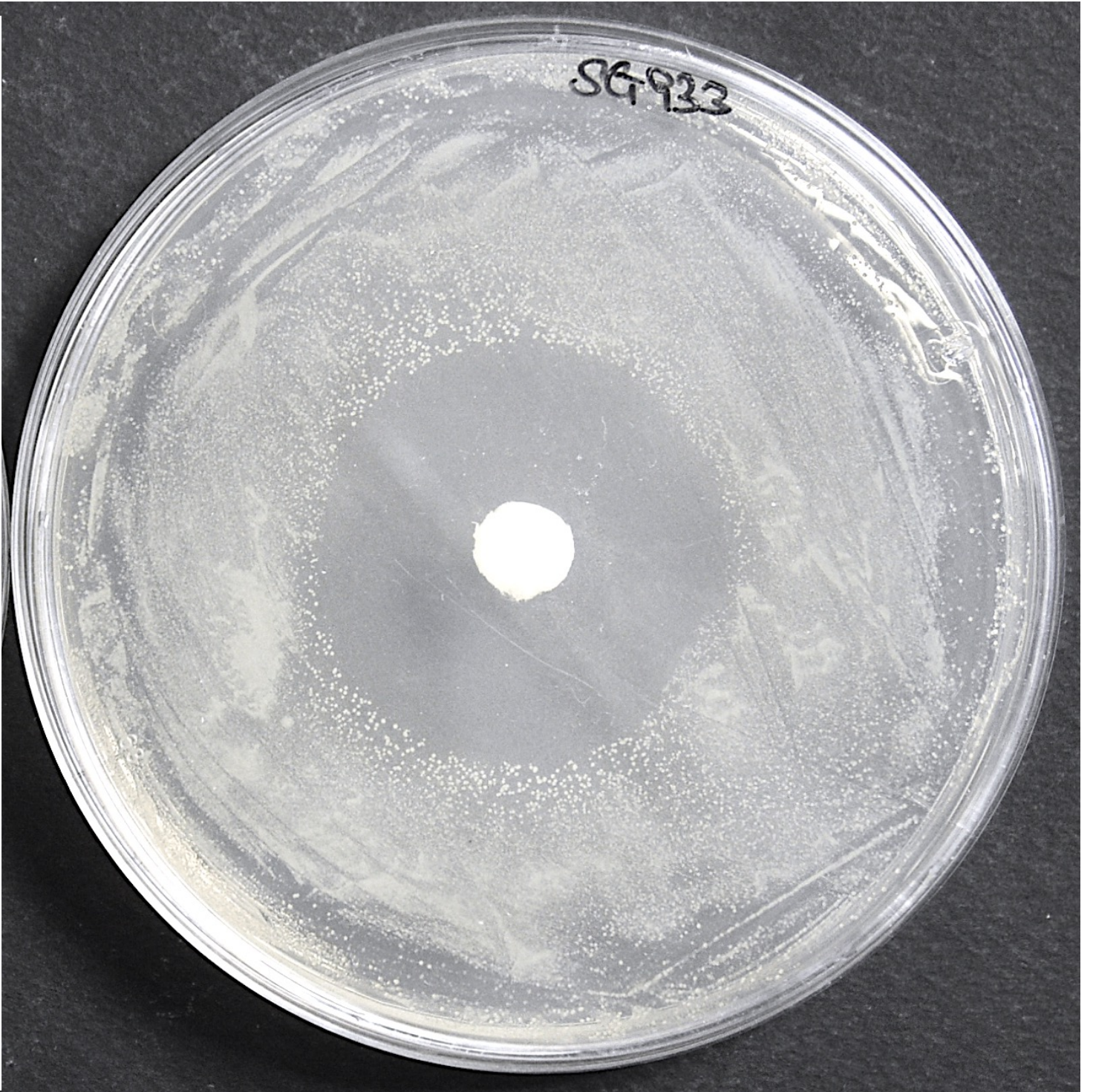

Supplement: Supplementary file 7 — Source data Fig. 2 [file 44319_2025_547_MOESM7_ESM.zip › Figure 2/Fig. 2C/Fig.2C.pdf]

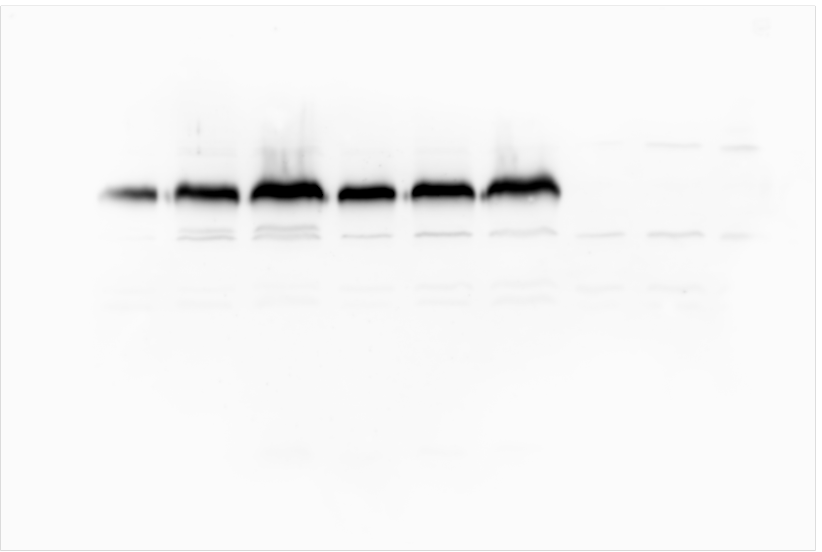

Supplement: Supplementary file 8 — Source data Fig. 3 [file 44319_2025_547_MOESM8_ESM.zip › Figure 3/Fig. 3C.tiff]

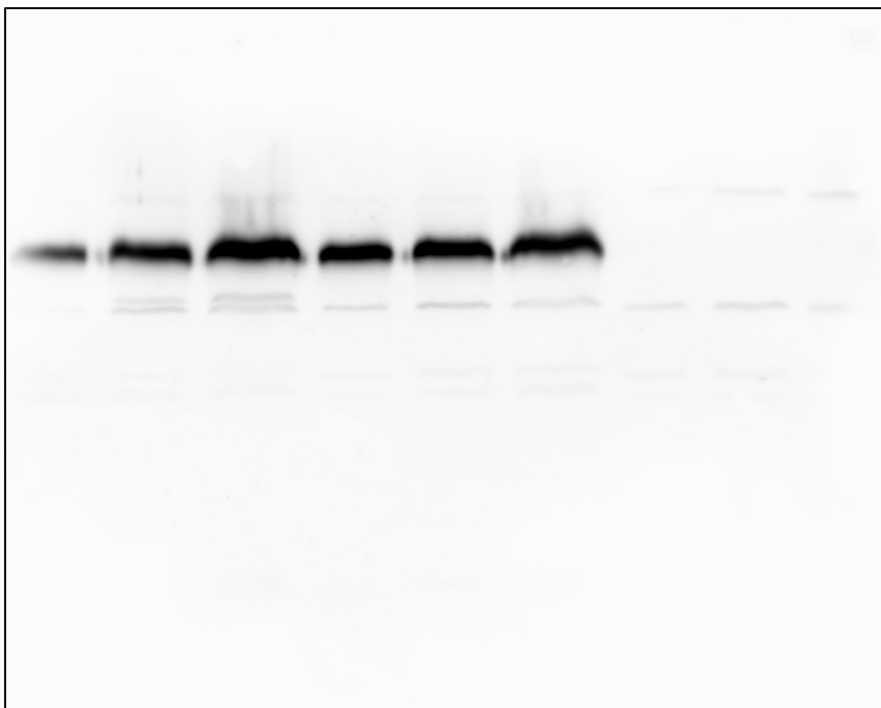

Supplement: Supplementary file 8 — Source data Fig. 3 [file 44319_2025_547_MOESM8_ESM.zip › Figure 3/Figure 3C.pdf]

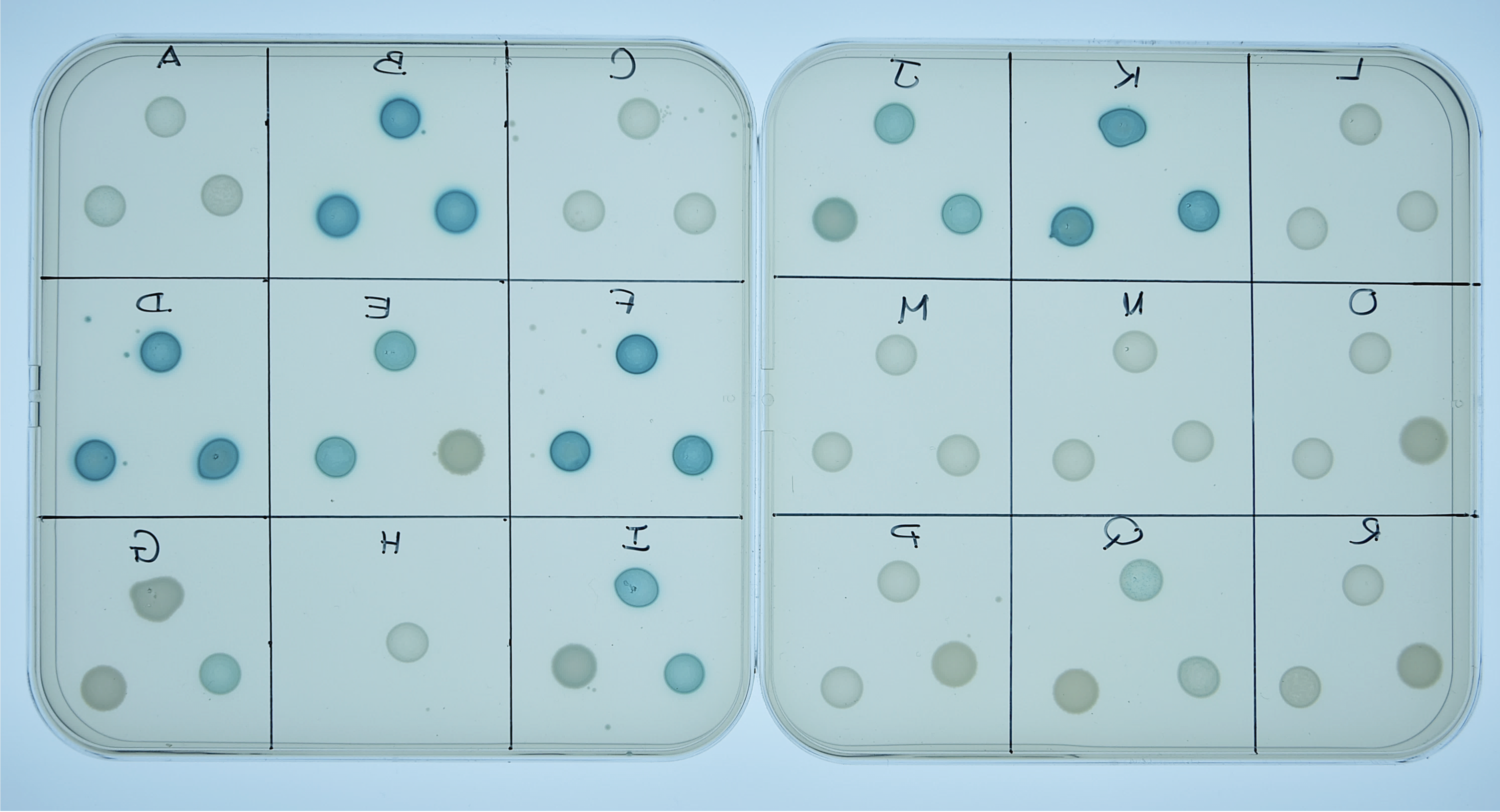

Supplement: Supplementary file 9 — Source data Fig. 4 [file 44319_2025_547_MOESM9_ESM.zip › Figure 4/Fig. 4A/B2H.tiff]

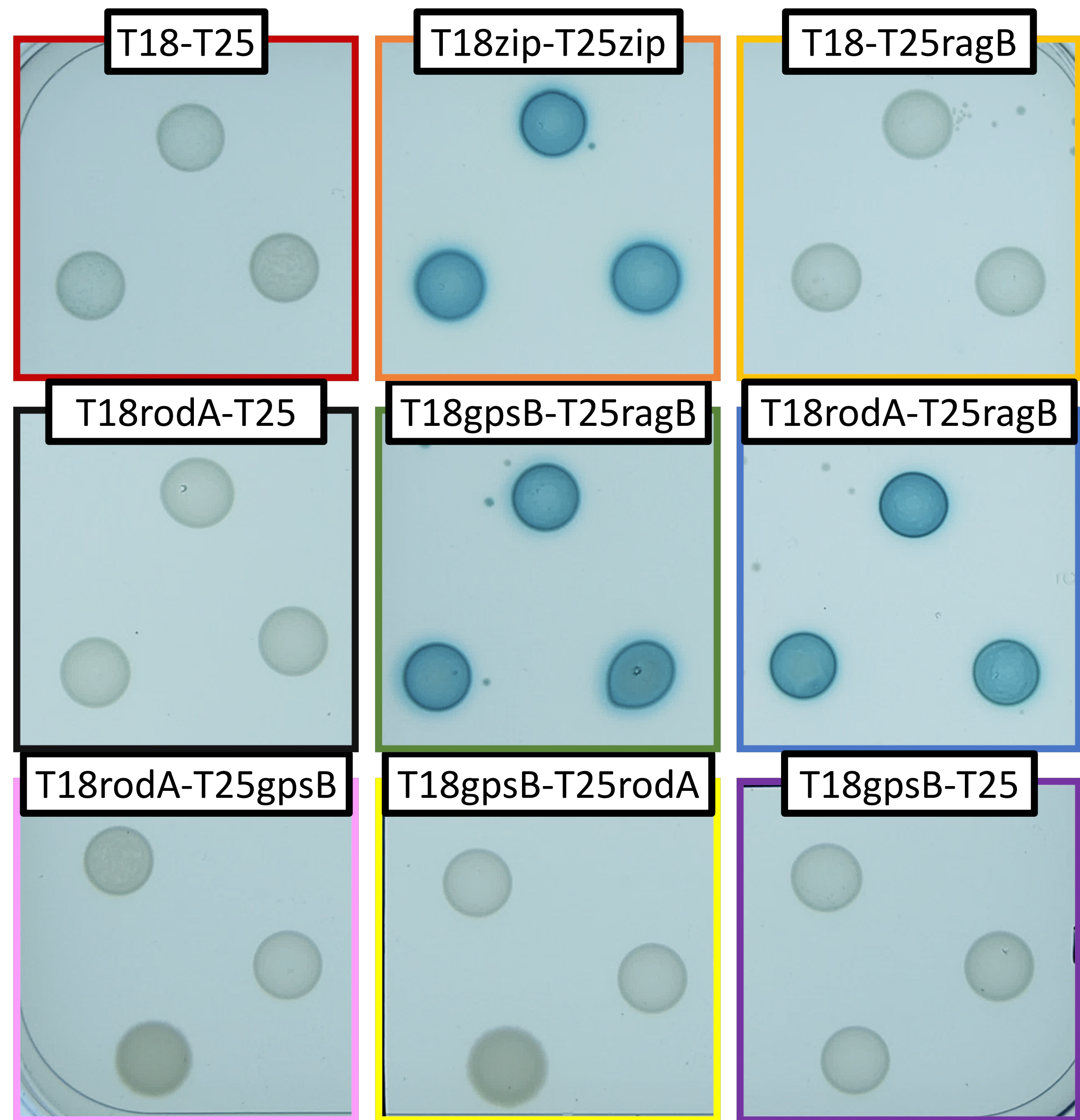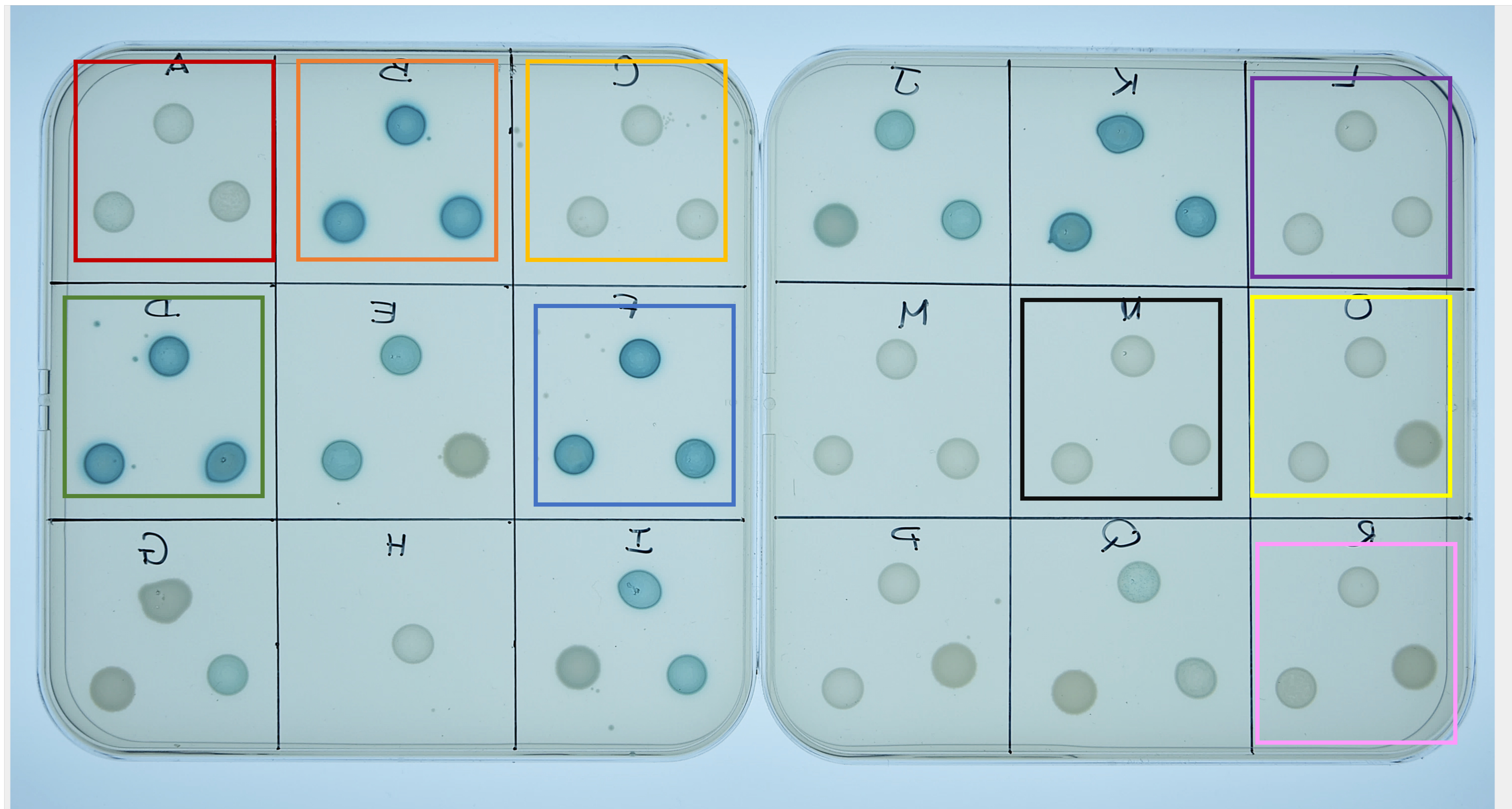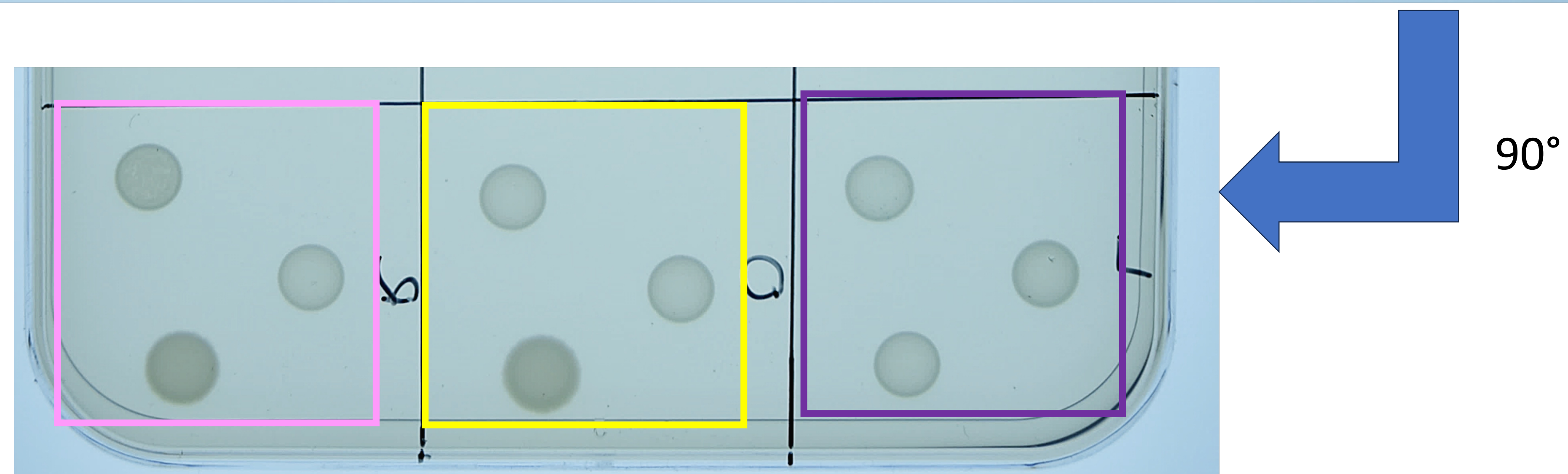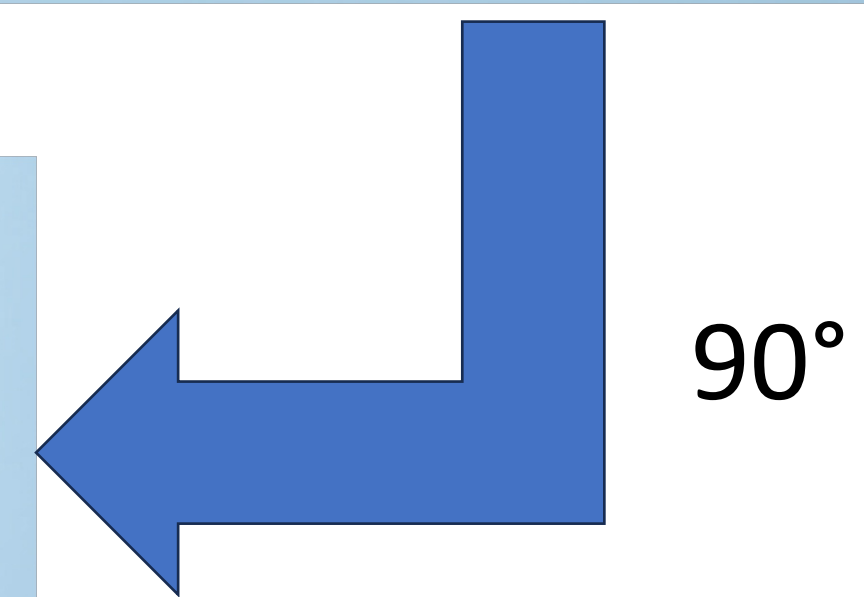

Supplement: Supplementary file 9 — Source data Fig. 4 [file 44319_2025_547_MOESM9_ESM.zip › Figure 4/Fig. 4A/Fig. 4A.pdf]

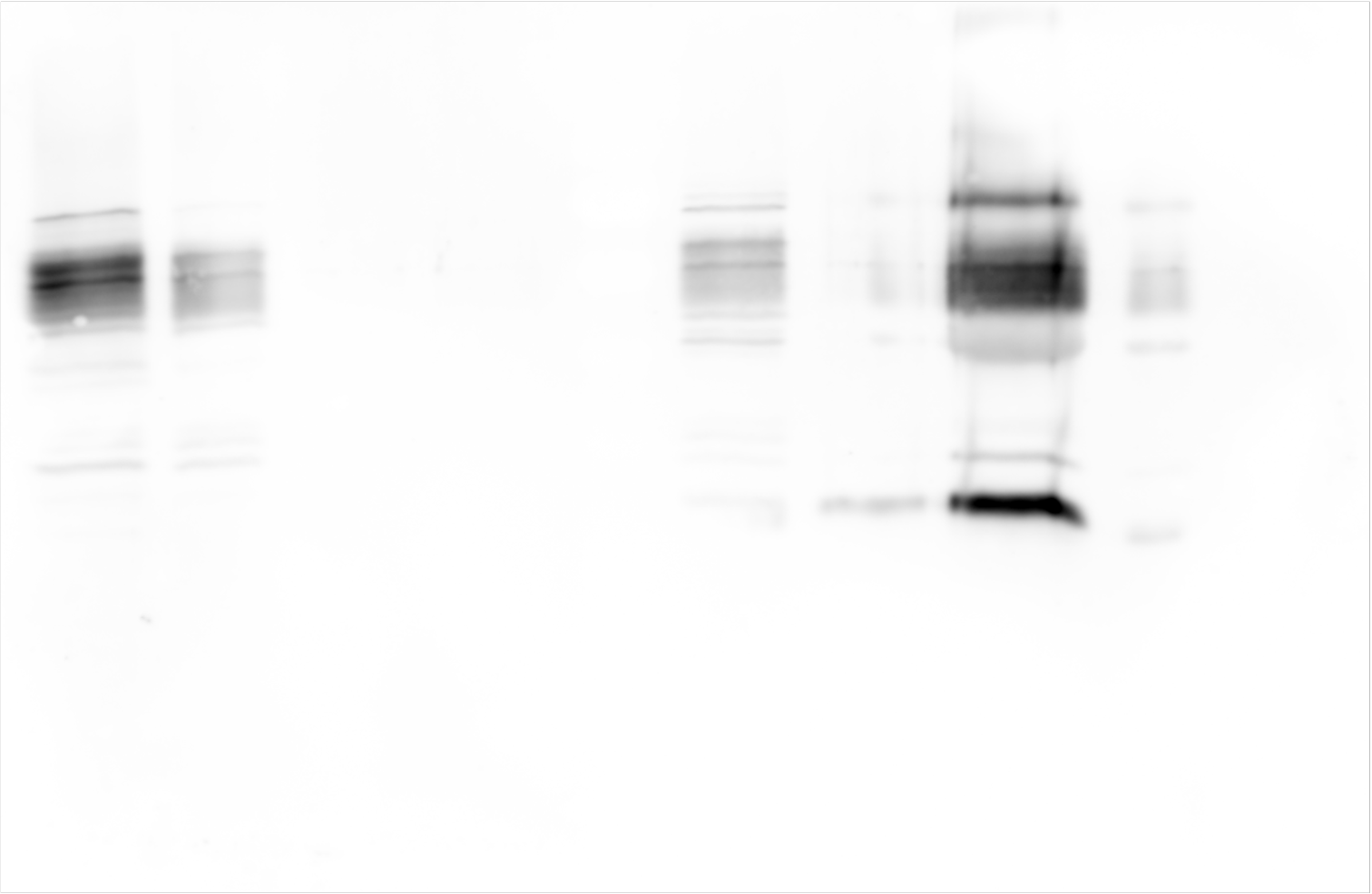

Supplement: Supplementary file 9 — Source data Fig. 4 [file 44319_2025_547_MOESM9_ESM.zip › Figure 4/Fig. 4B /1.tiff]

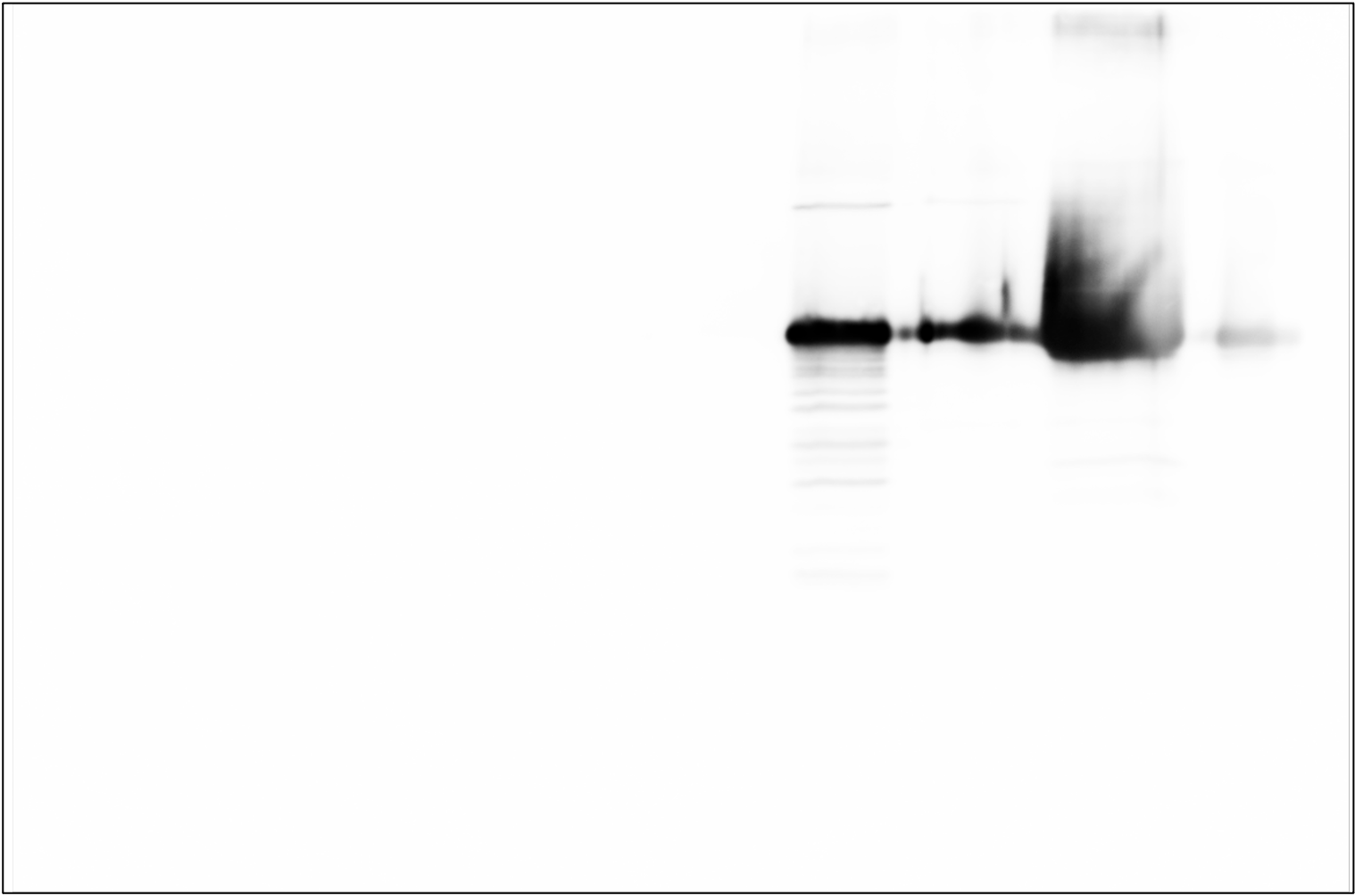

Supplement: Supplementary file 9 — Source data Fig. 4 [file 44319_2025_547_MOESM9_ESM.zip › Figure 4/Fig. 4B /2.tiff]

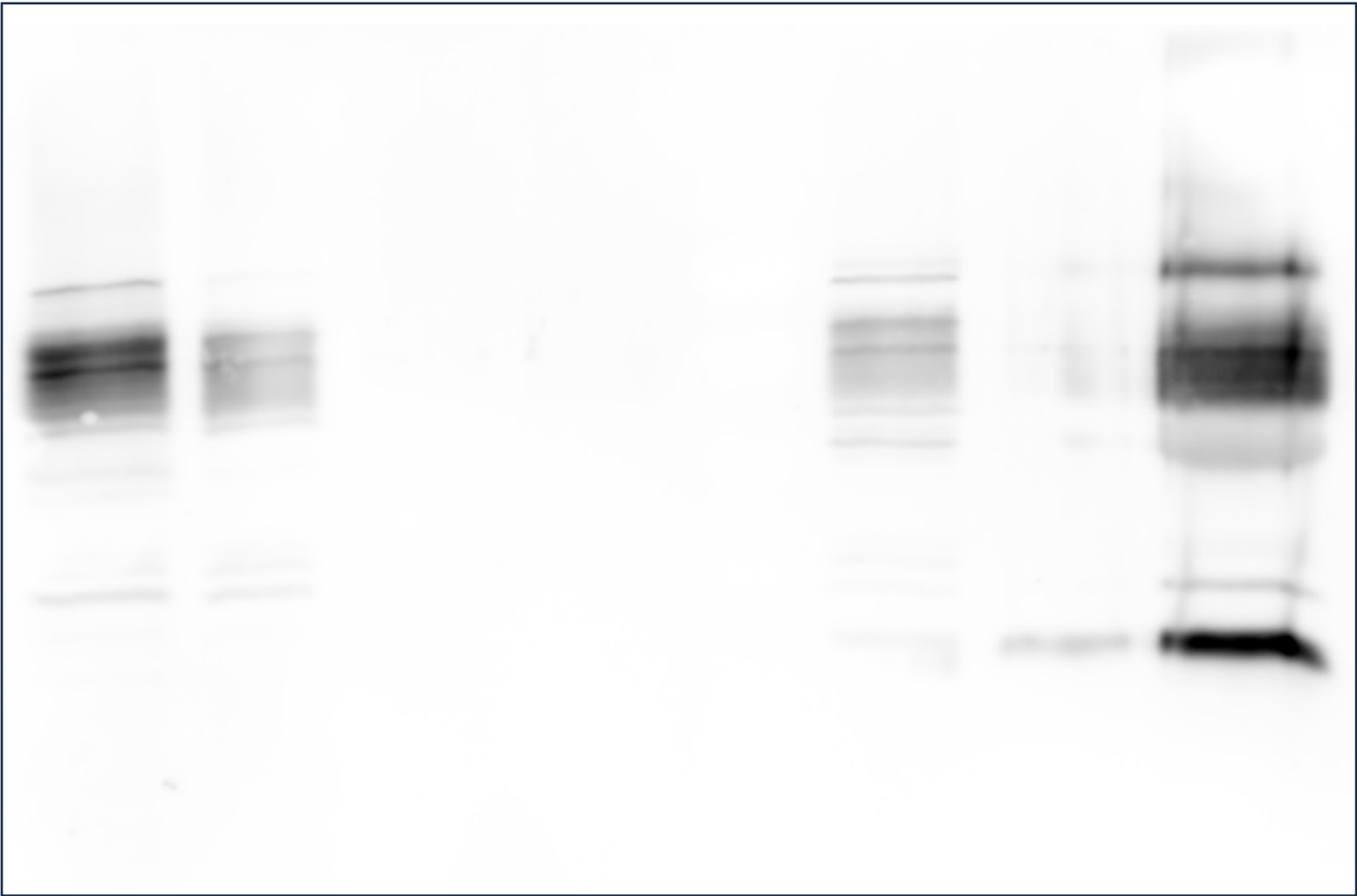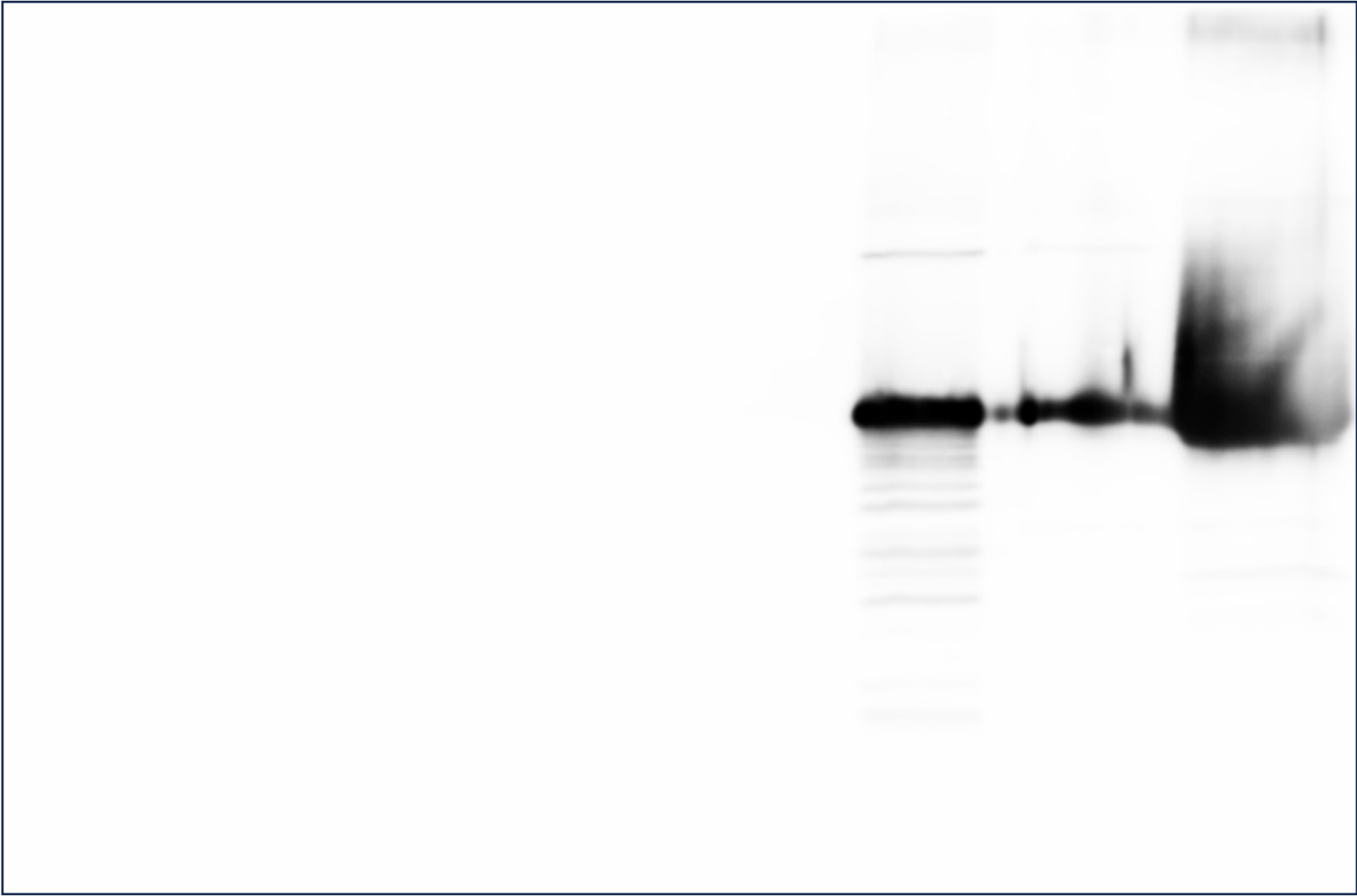

Supplement: Supplementary file 9 — Source data Fig. 4 [file 44319_2025_547_MOESM9_ESM.zip › Figure 4/Fig. 4B /Fig. 4B.pdf]

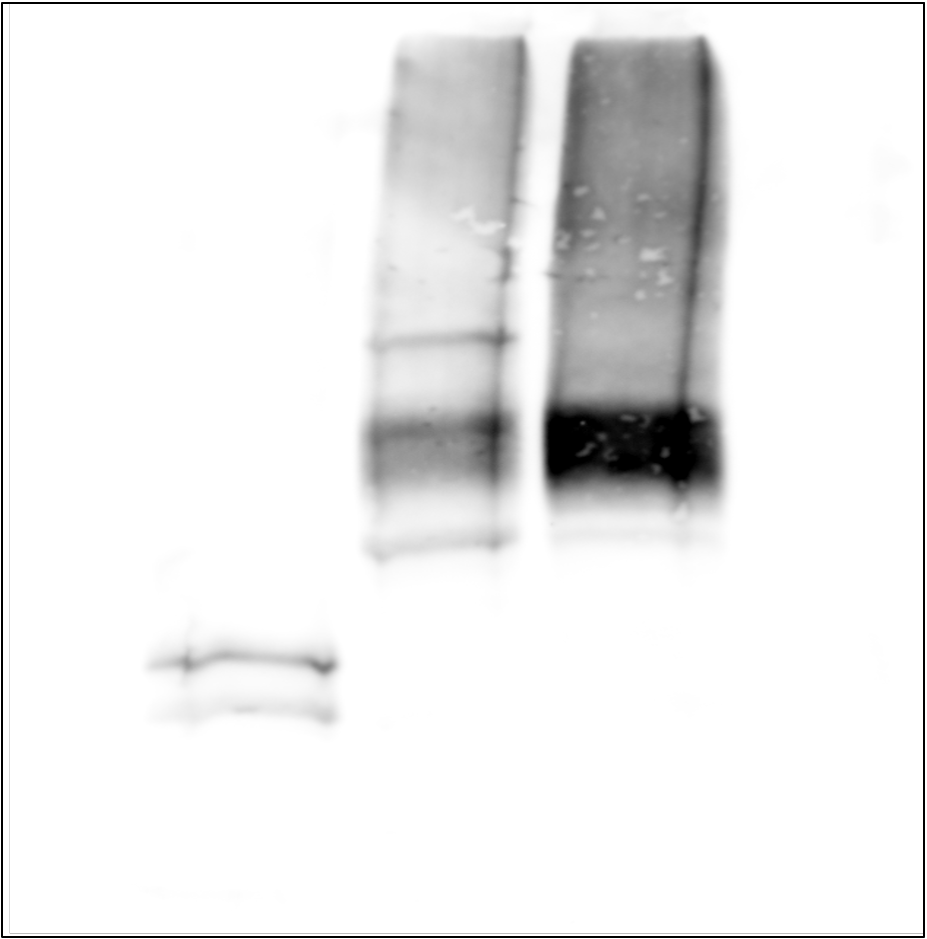

Supplement: Supplementary file 9 — Source data Fig. 4 [file 44319_2025_547_MOESM9_ESM.zip › Figure 4/Fig. 4C/3.tiff]

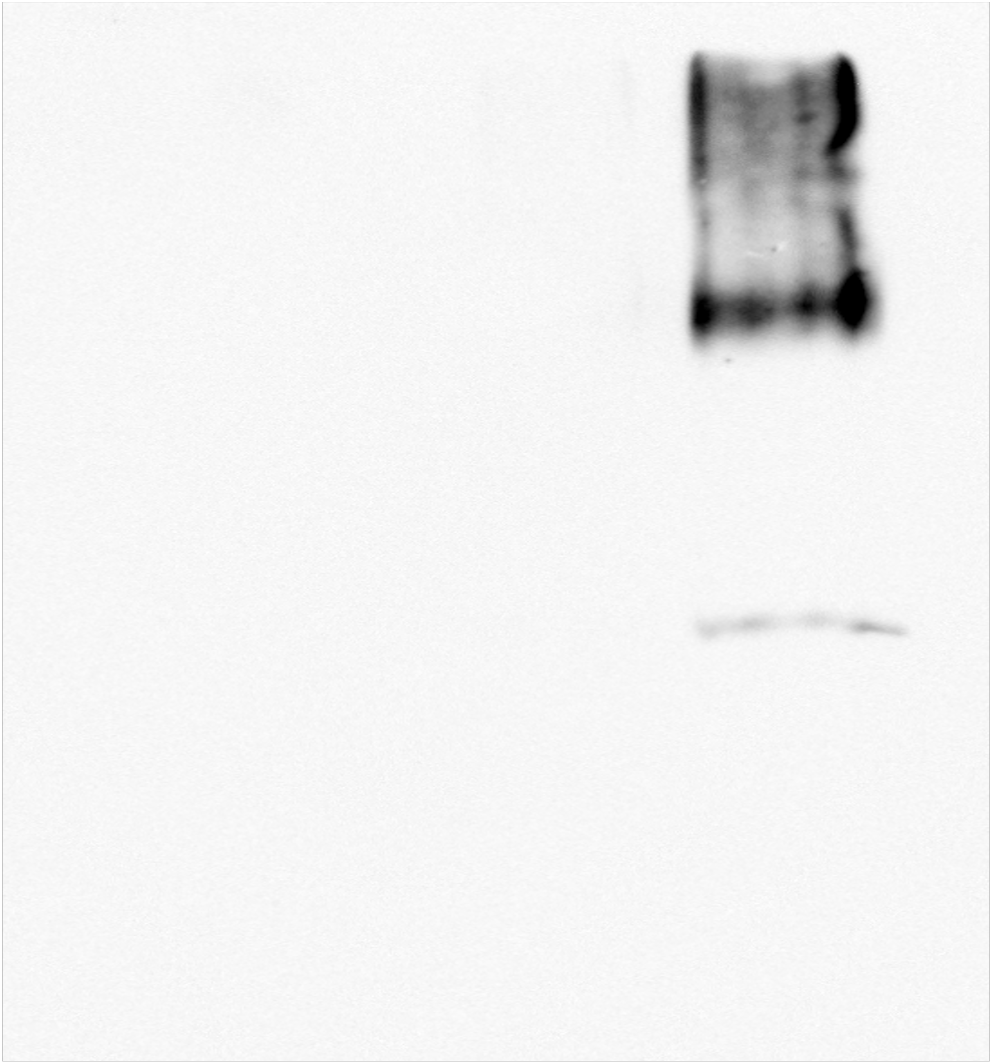

Supplement: Supplementary file 9 — Source data Fig. 4 [file 44319_2025_547_MOESM9_ESM.zip › Figure 4/Fig. 4C/4.tiff]

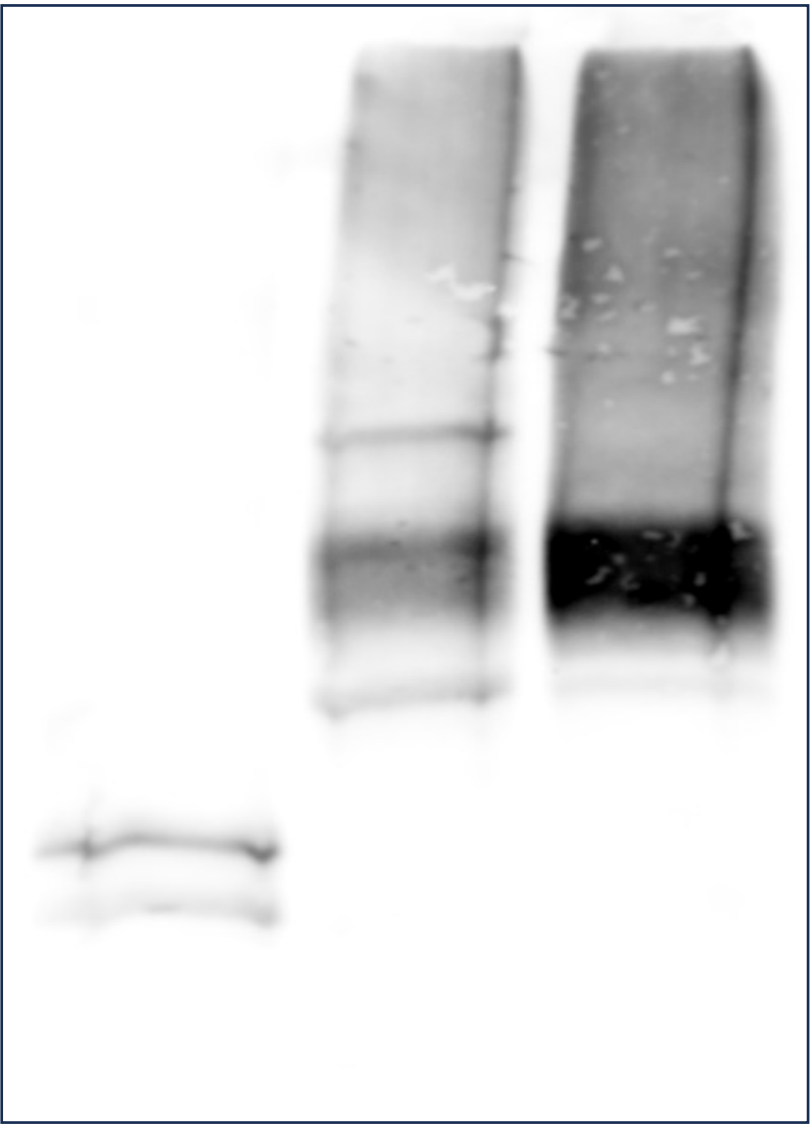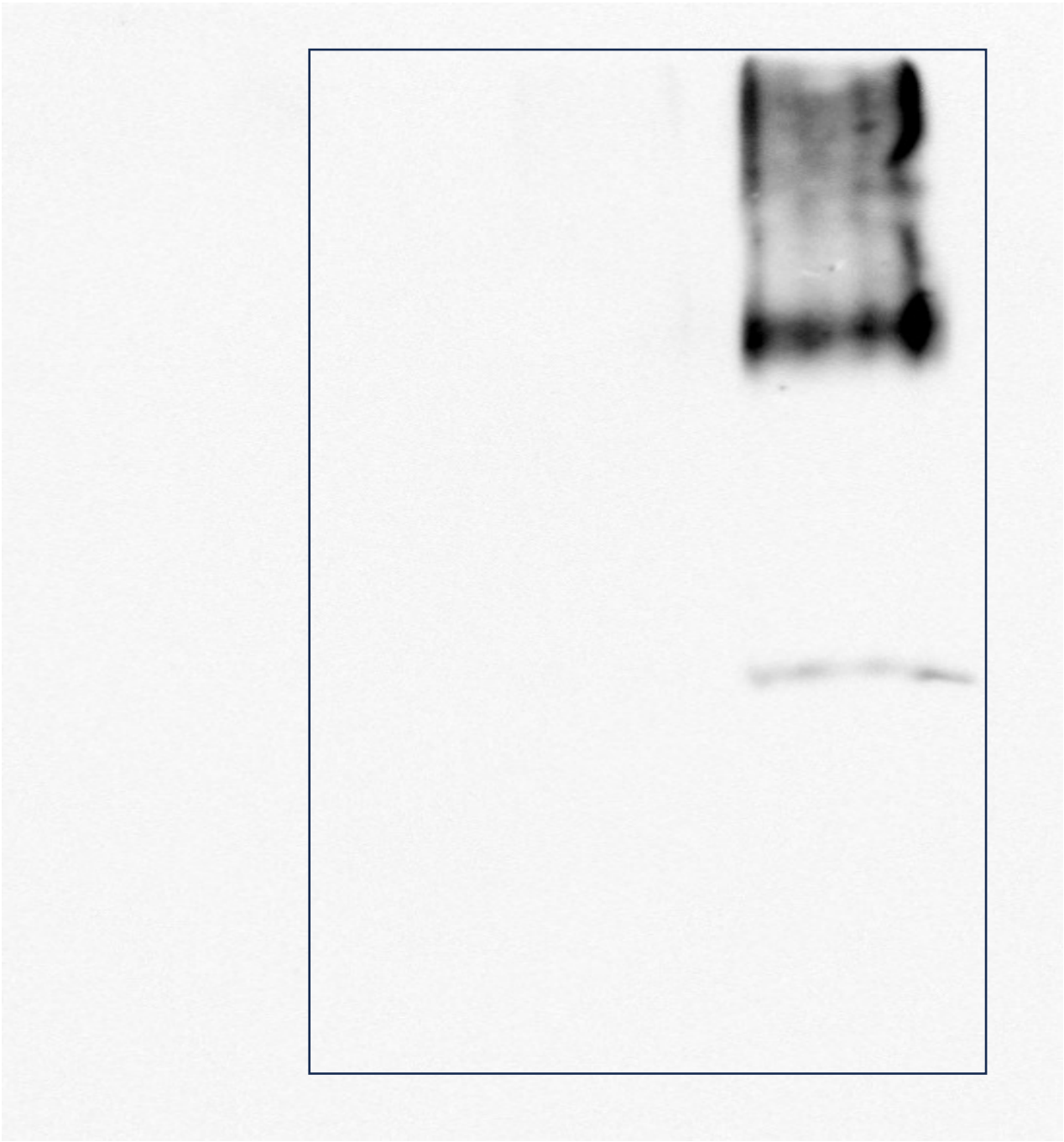

Supplement: Supplementary file 9 — Source data Fig. 4 [file 44319_2025_547_MOESM9_ESM.zip › Figure 4/Fig. 4C/Fig. 4C.pdf]

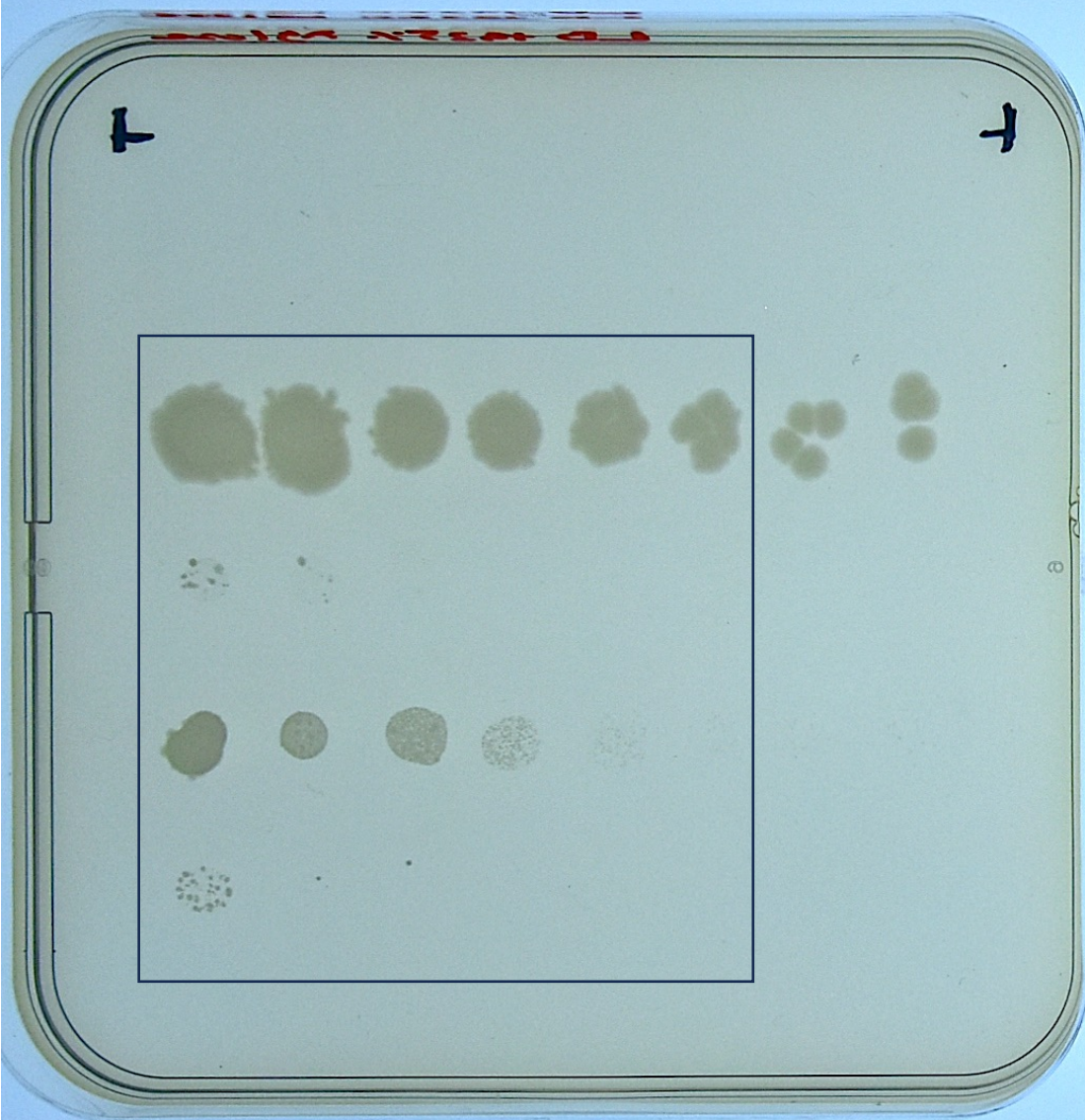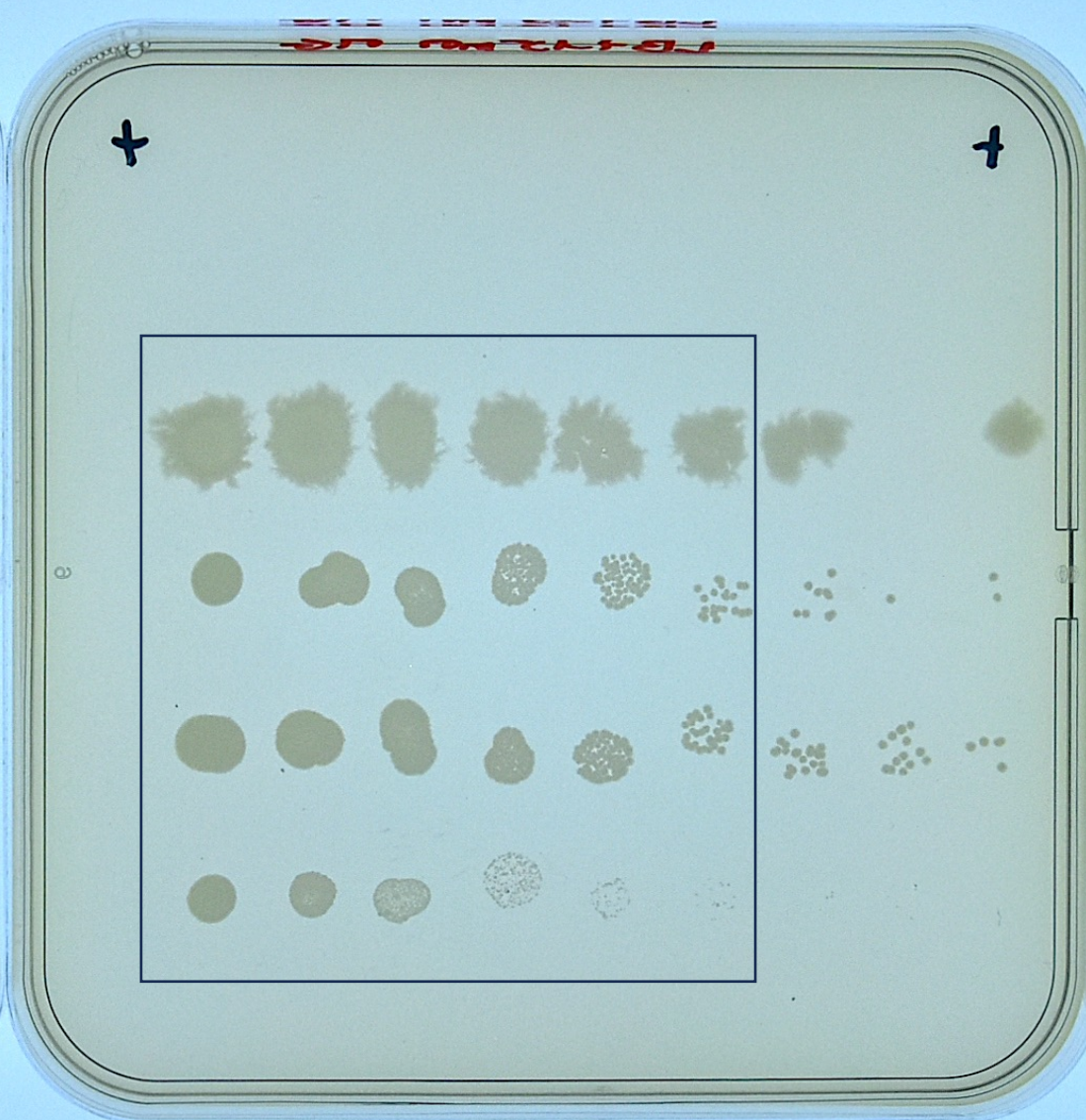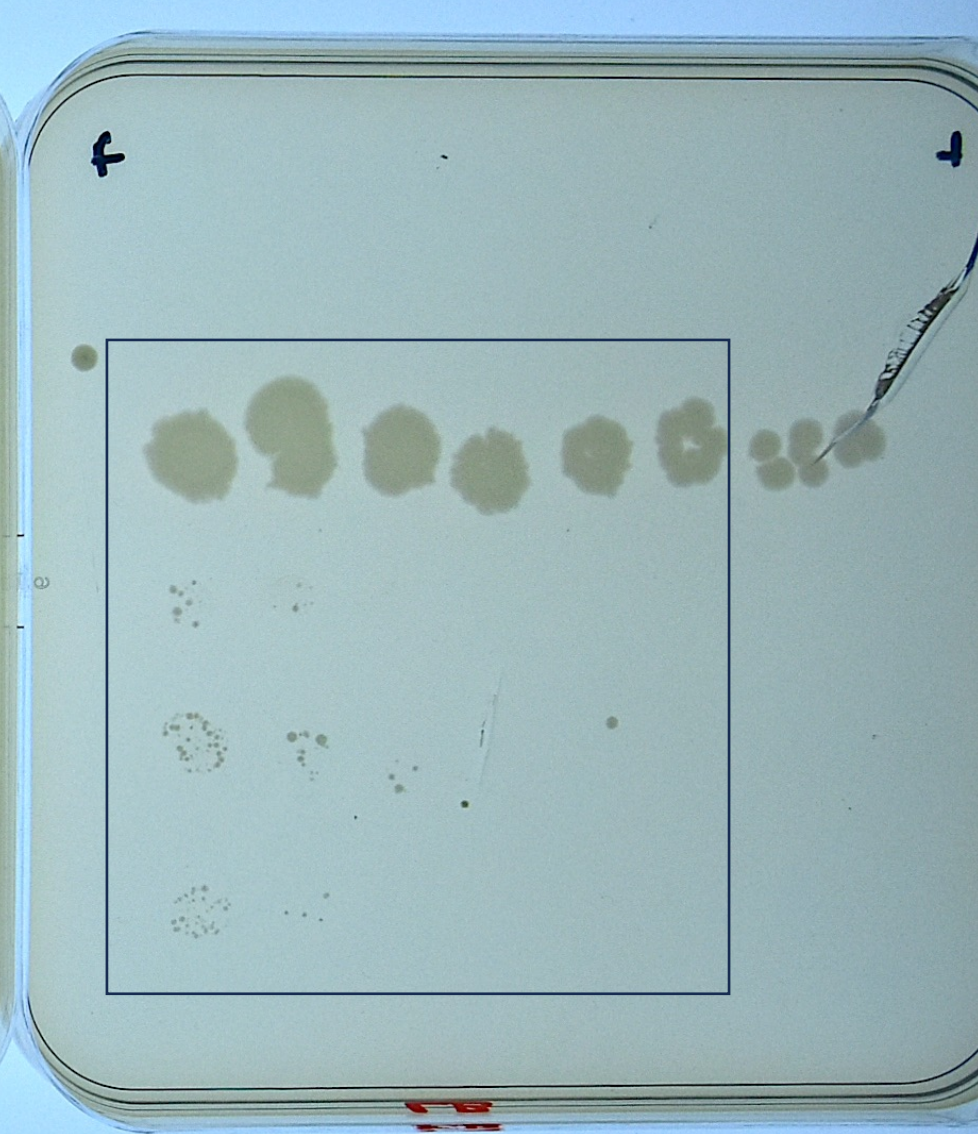

Supplement: Supplementary file 10 — Source data Fig. 5 [file 44319_2025_547_MOESM10_ESM.zip › Figure 5/Fig. 5A/Fig. 5A.pdf]

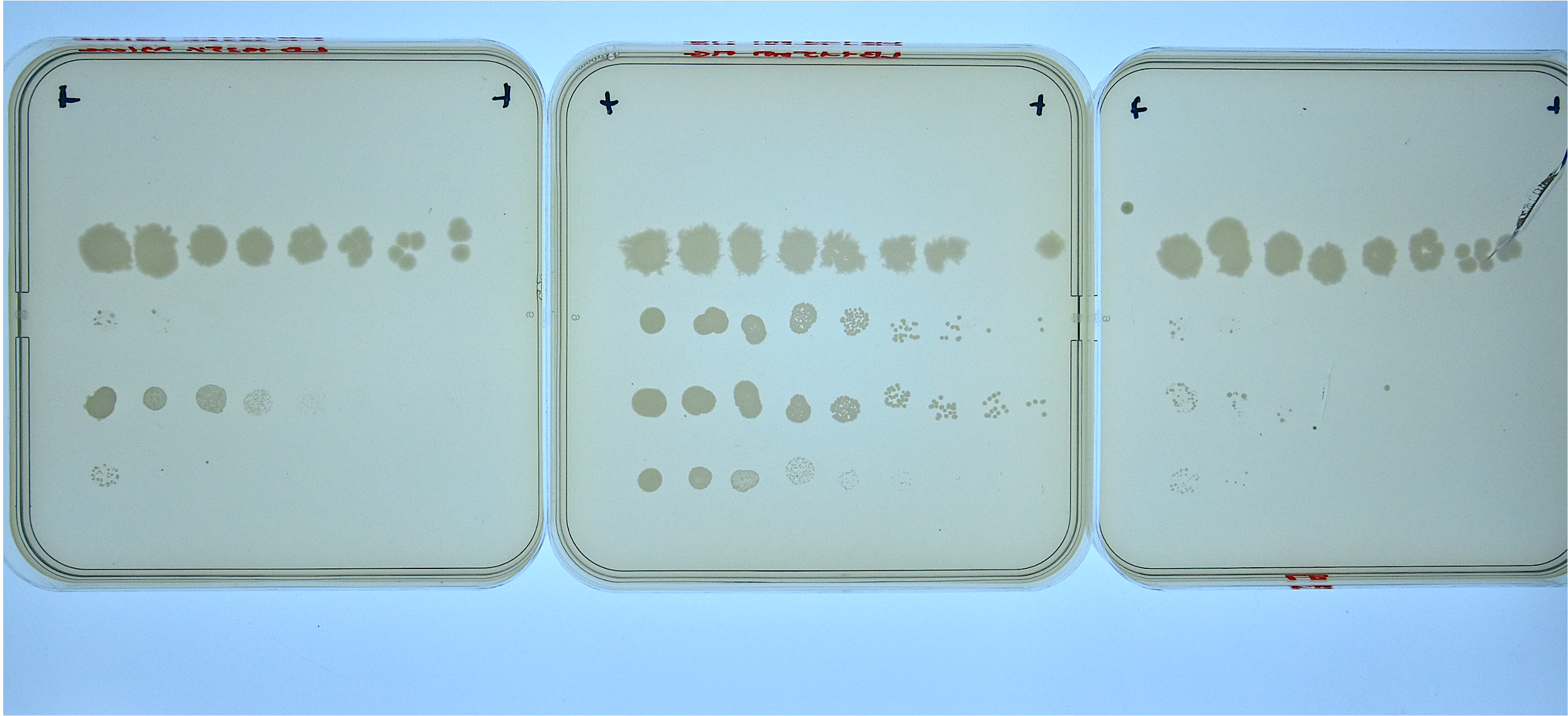

Supplement: Supplementary file 10 — Source data Fig. 5 [file 44319_2025_547_MOESM10_ESM.zip › Figure 5/Fig. 5A/Fig. 5A.tiff]

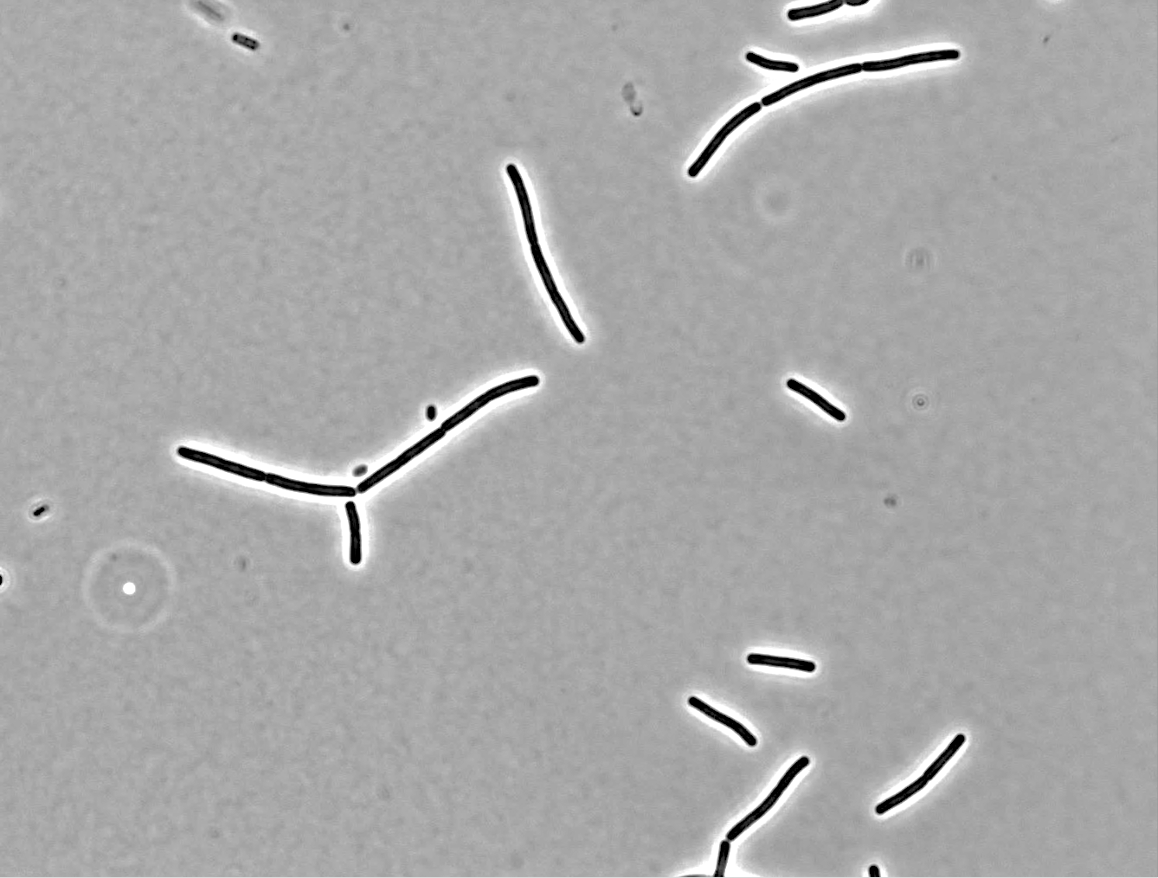

Supplement: Supplementary file 10 — Source data Fig. 5 [file 44319_2025_547_MOESM10_ESM.zip › Figure 5/Fig. 5B/delta4 and rodA.tiff]

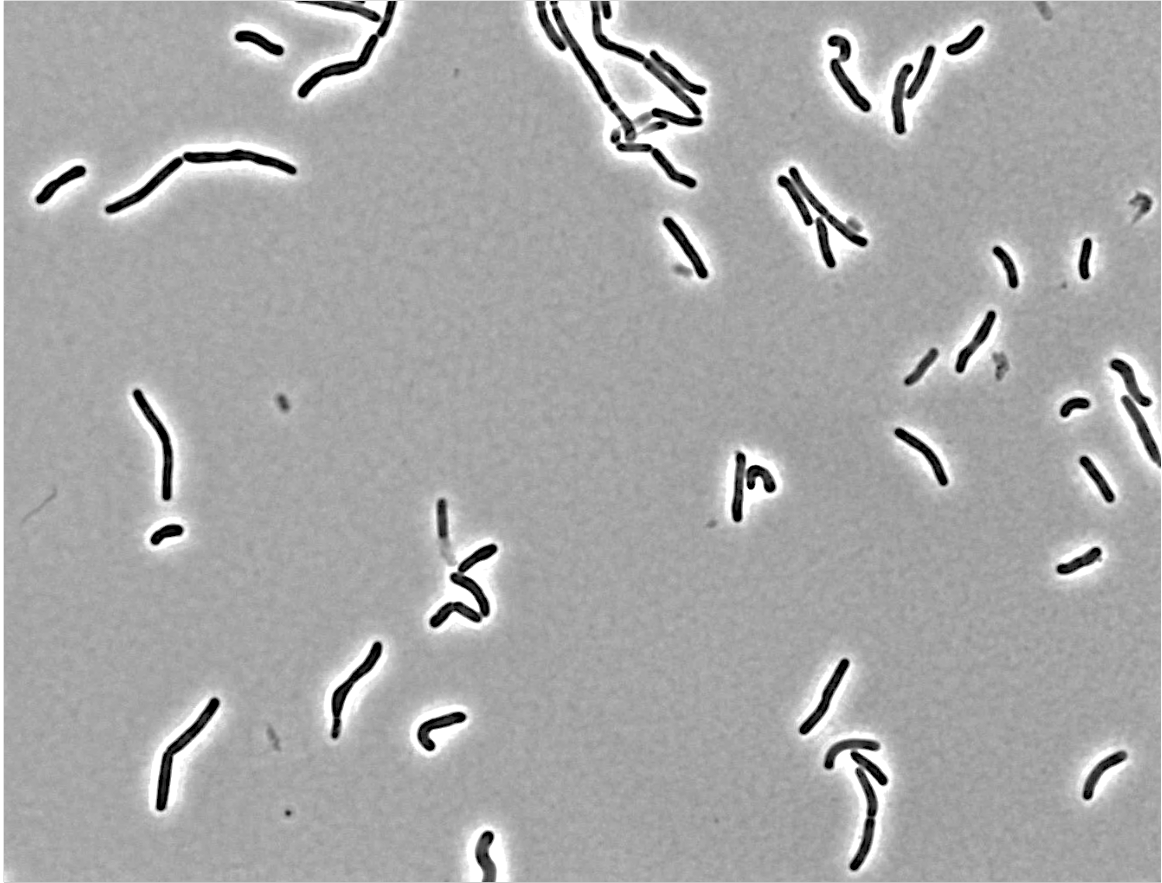

Supplement: Supplementary file 10 — Source data Fig. 5 [file 44319_2025_547_MOESM10_ESM.zip › Figure 5/Fig. 5B/delta4 delta ragB and RodA.tiff]

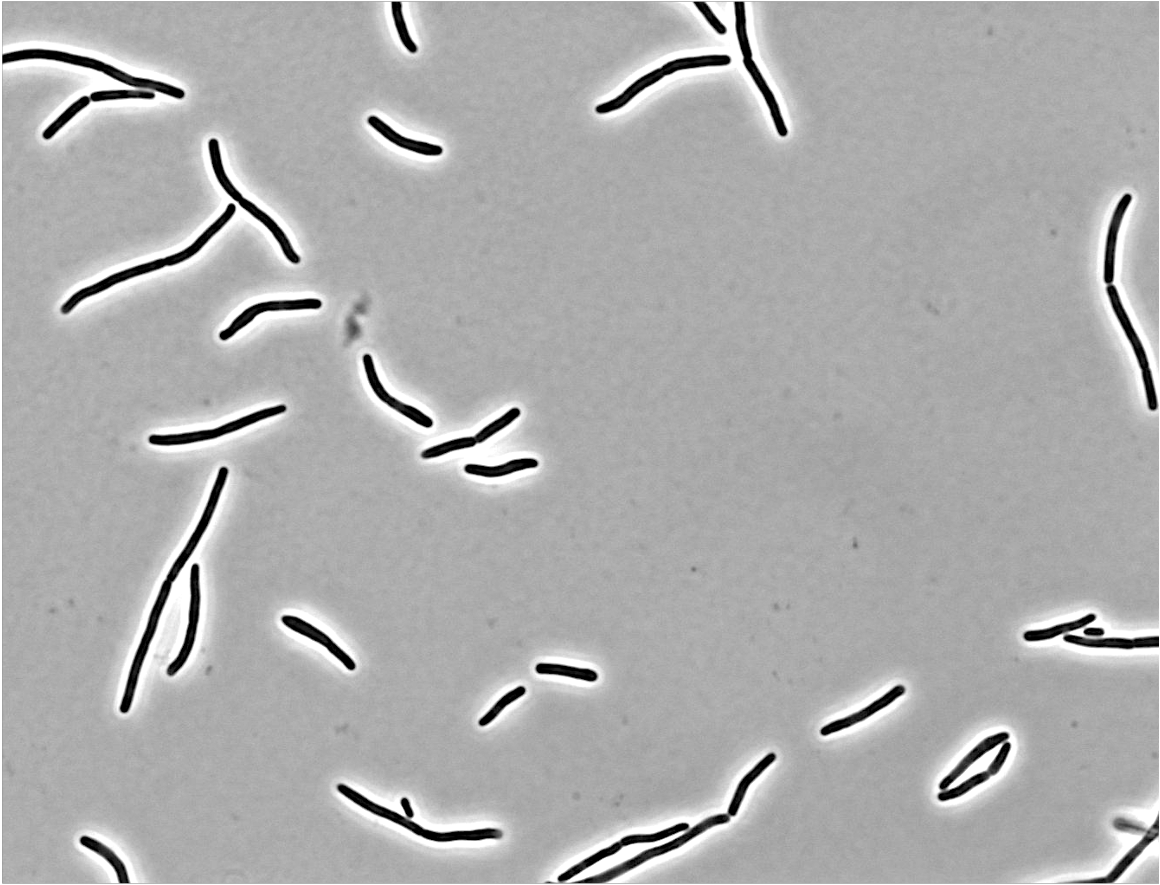

Supplement: Supplementary file 10 — Source data Fig. 5 [file 44319_2025_547_MOESM10_ESM.zip › Figure 5/Fig. 5B/Delta4.tiff]

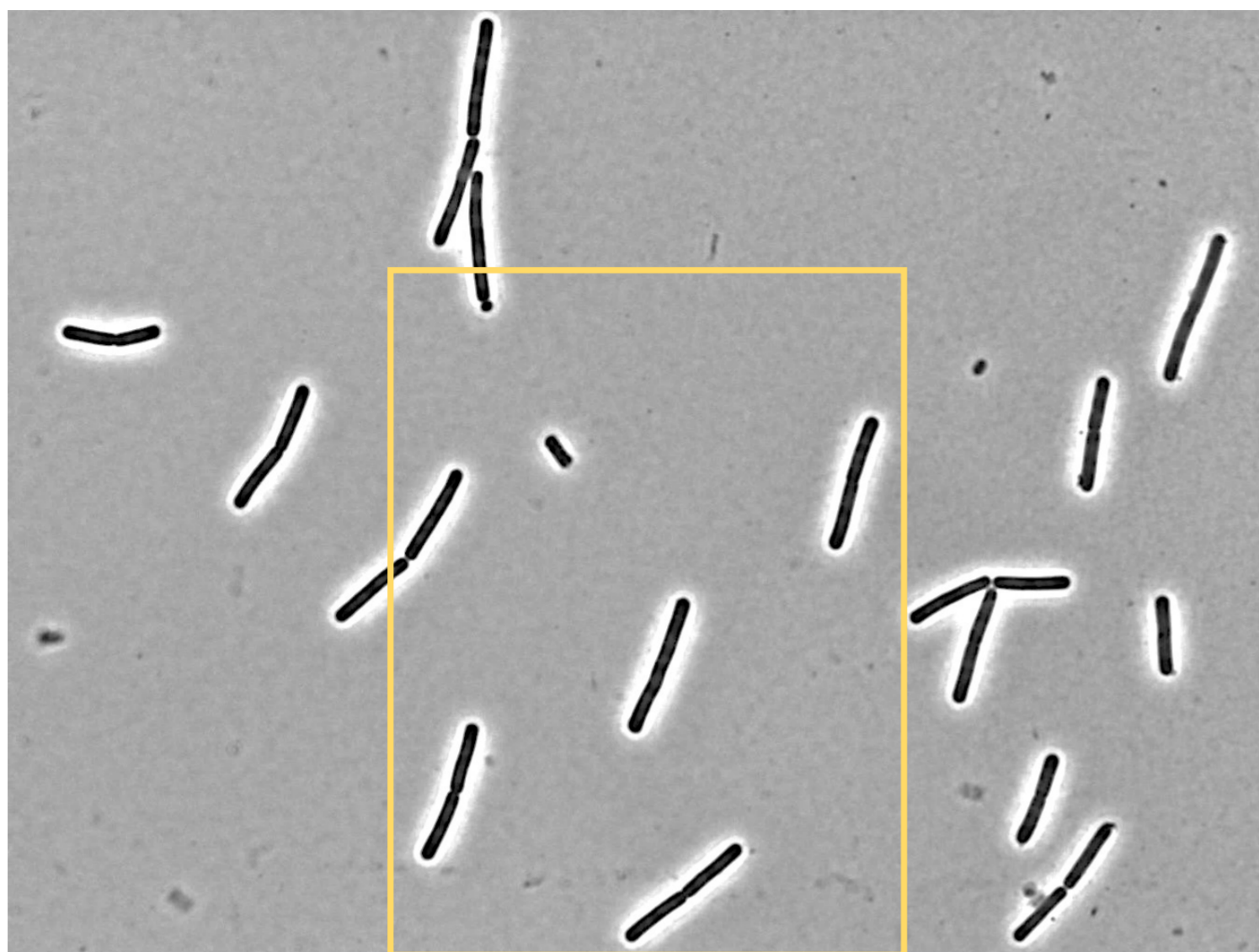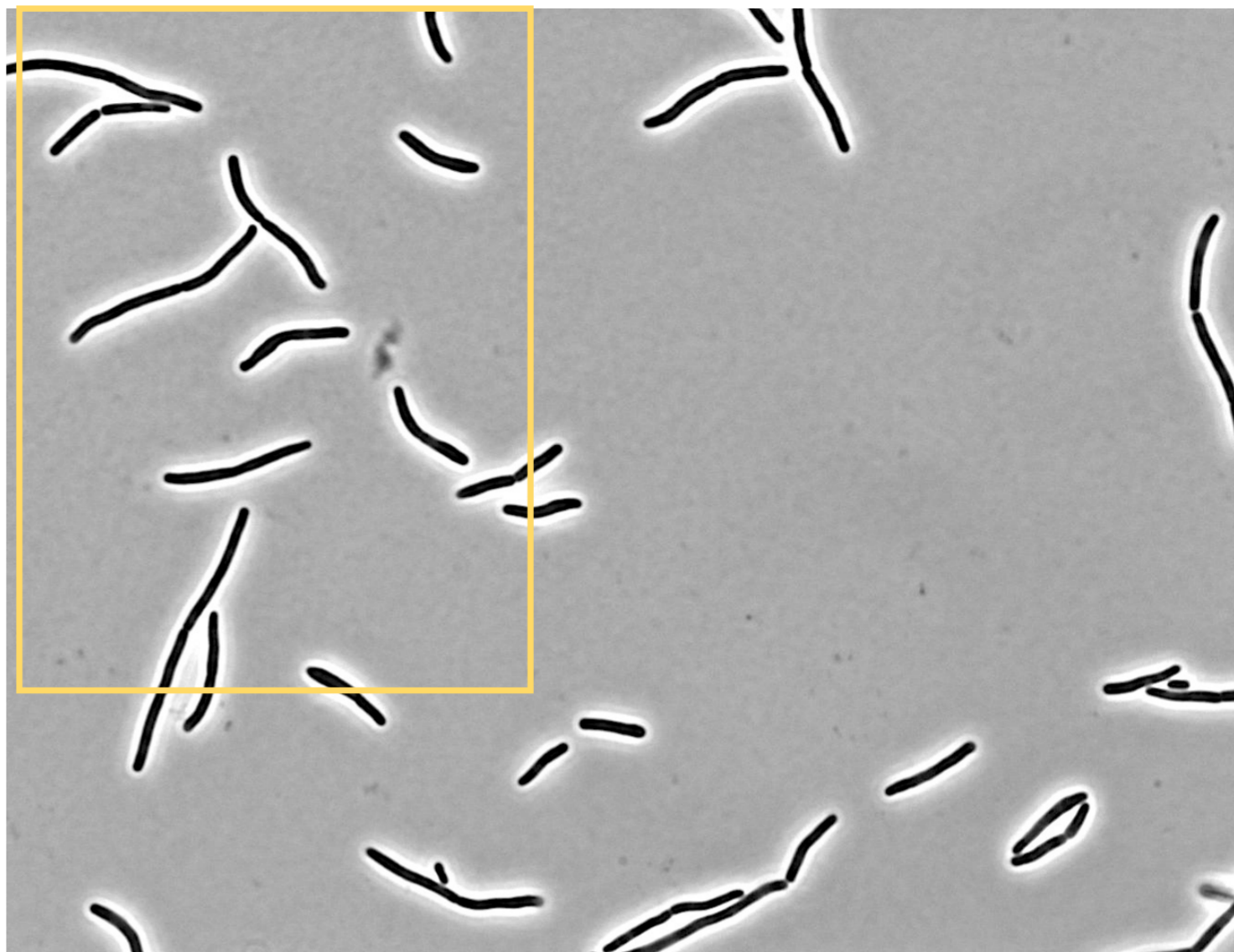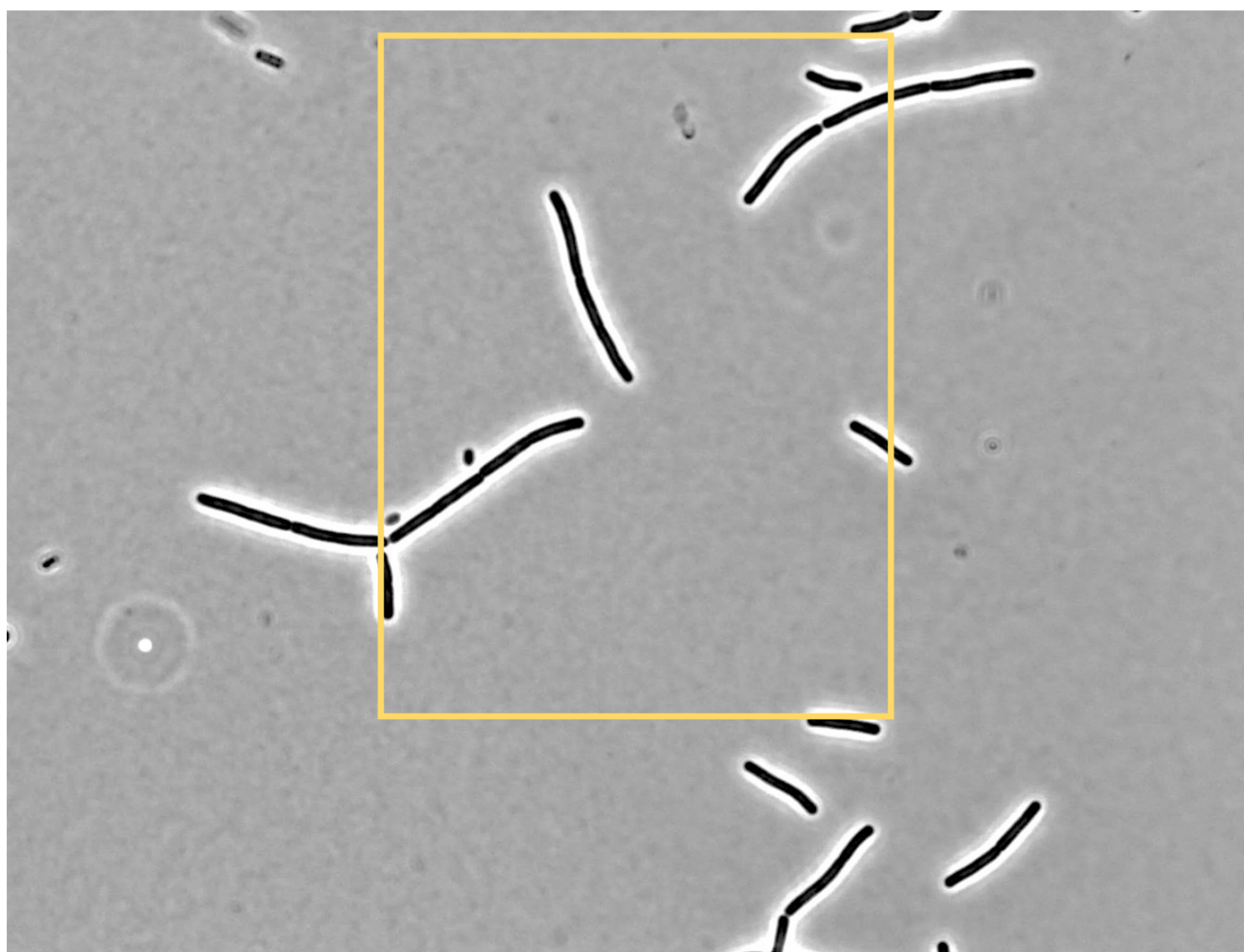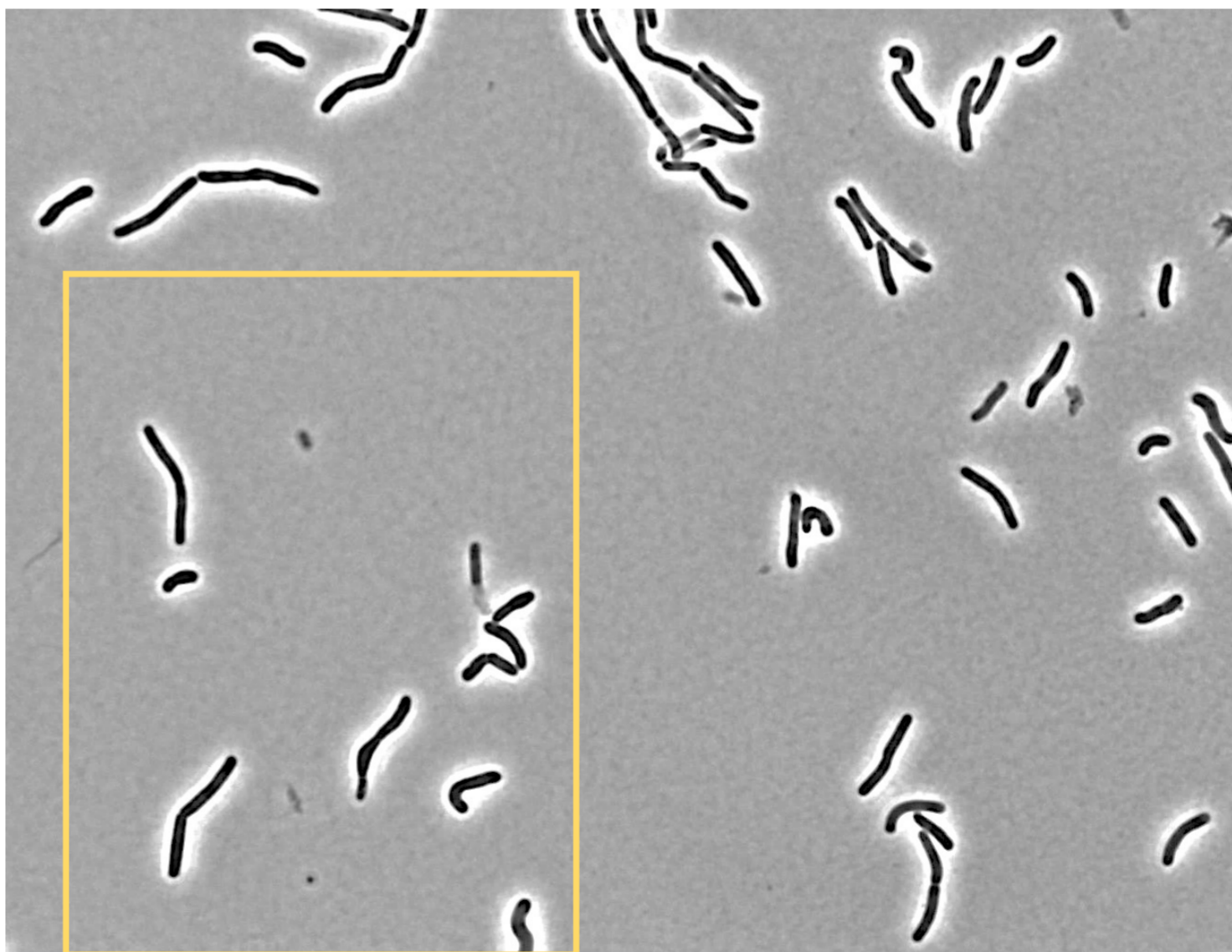

Supplement: Supplementary file 10 — Source data Fig. 5 [file 44319_2025_547_MOESM10_ESM.zip › Figure 5/Fig. 5B/Fig. 5B.pdf]

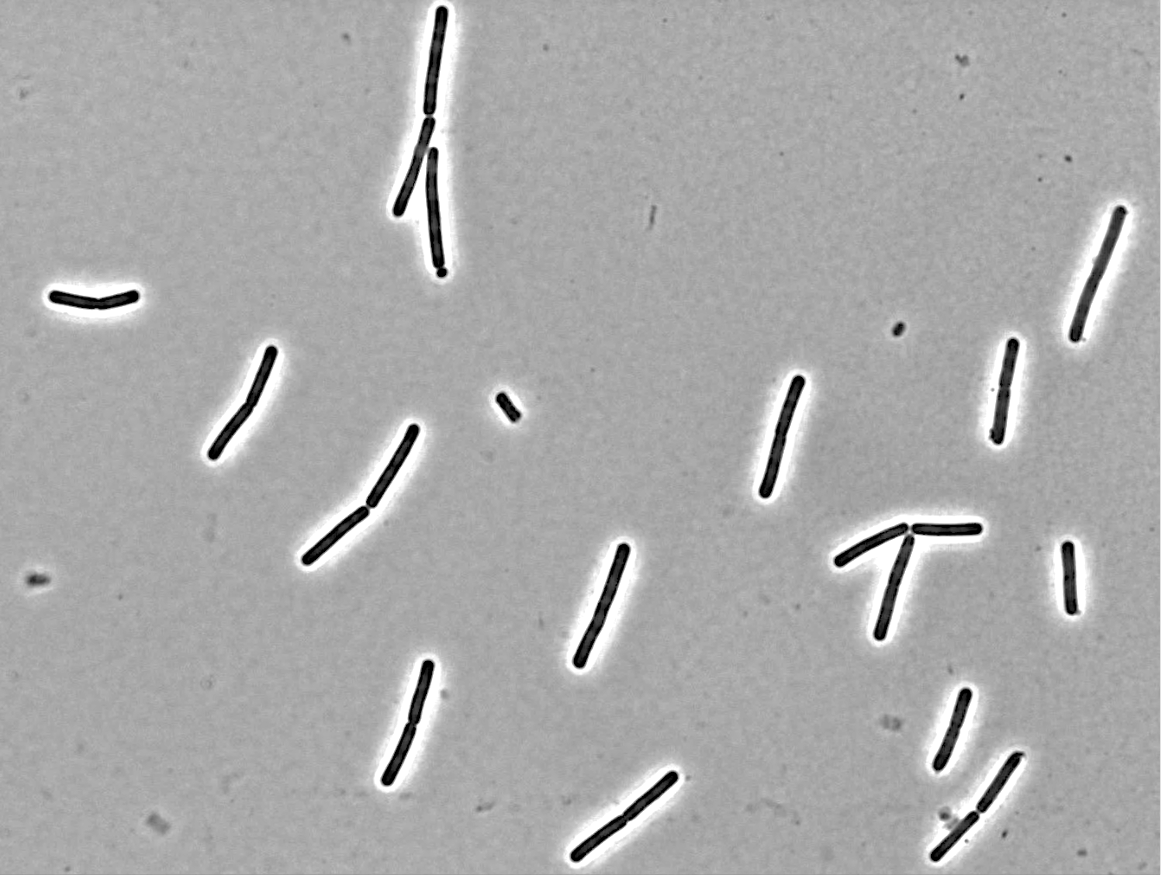

Supplement: Supplementary file 10 — Source data Fig. 5 [file 44319_2025_547_MOESM10_ESM.zip › Figure 5/Fig. 5B/WT.tiff]

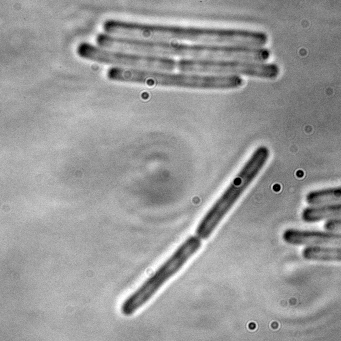

Supplement: Supplementary file 11 — Source data Fig. 6 [file 44319_2025_547_MOESM11_ESM.zip › Figure 6/Fig. 6D.tif]

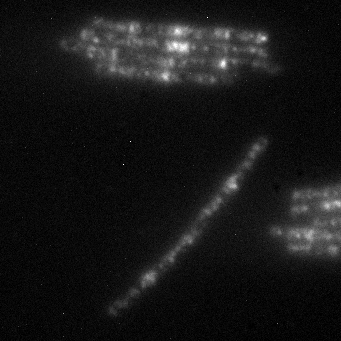

Supplement: Supplementary file 11 — Source data Fig. 6 [file 44319_2025_547_MOESM11_ESM.zip › Figure 6/Fig. 6E.tif]

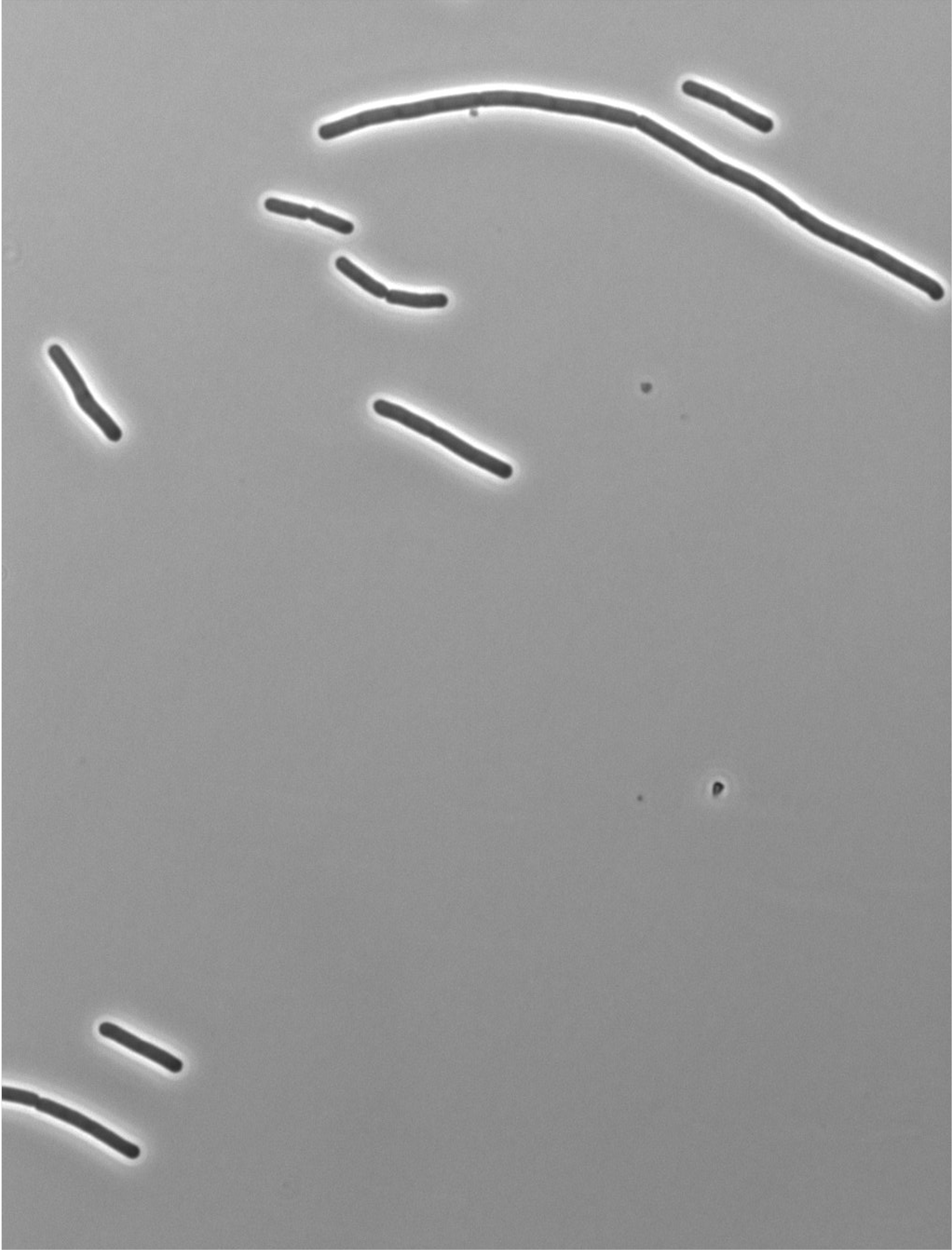

Supplement: Supplementary file 11 — Source data Fig. 6 [file 44319_2025_547_MOESM11_ESM.zip › Figure 6/Fig. 6B/B1.tiff]

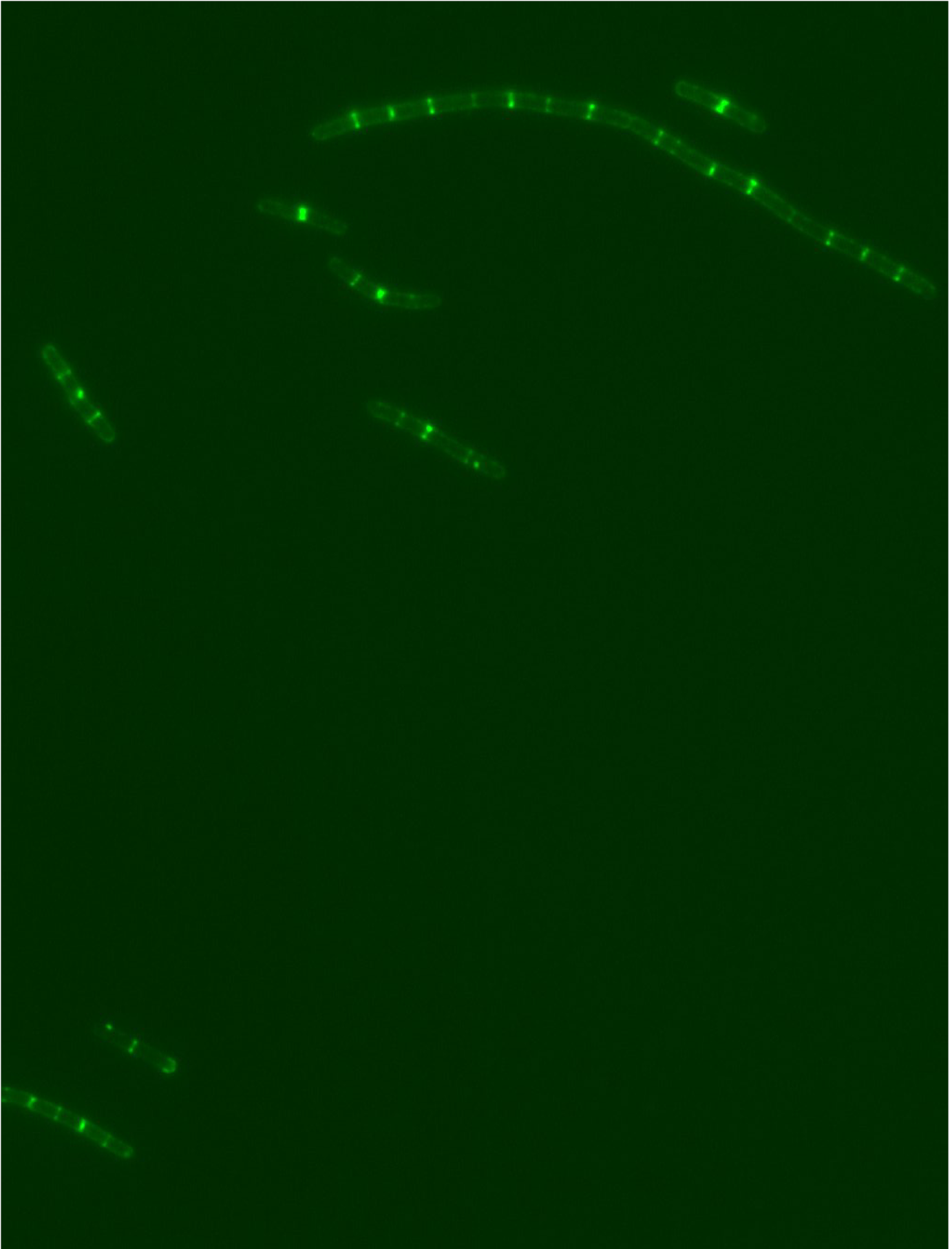

Supplement: Supplementary file 11 — Source data Fig. 6 [file 44319_2025_547_MOESM11_ESM.zip › Figure 6/Fig. 6B/B2.tiff]

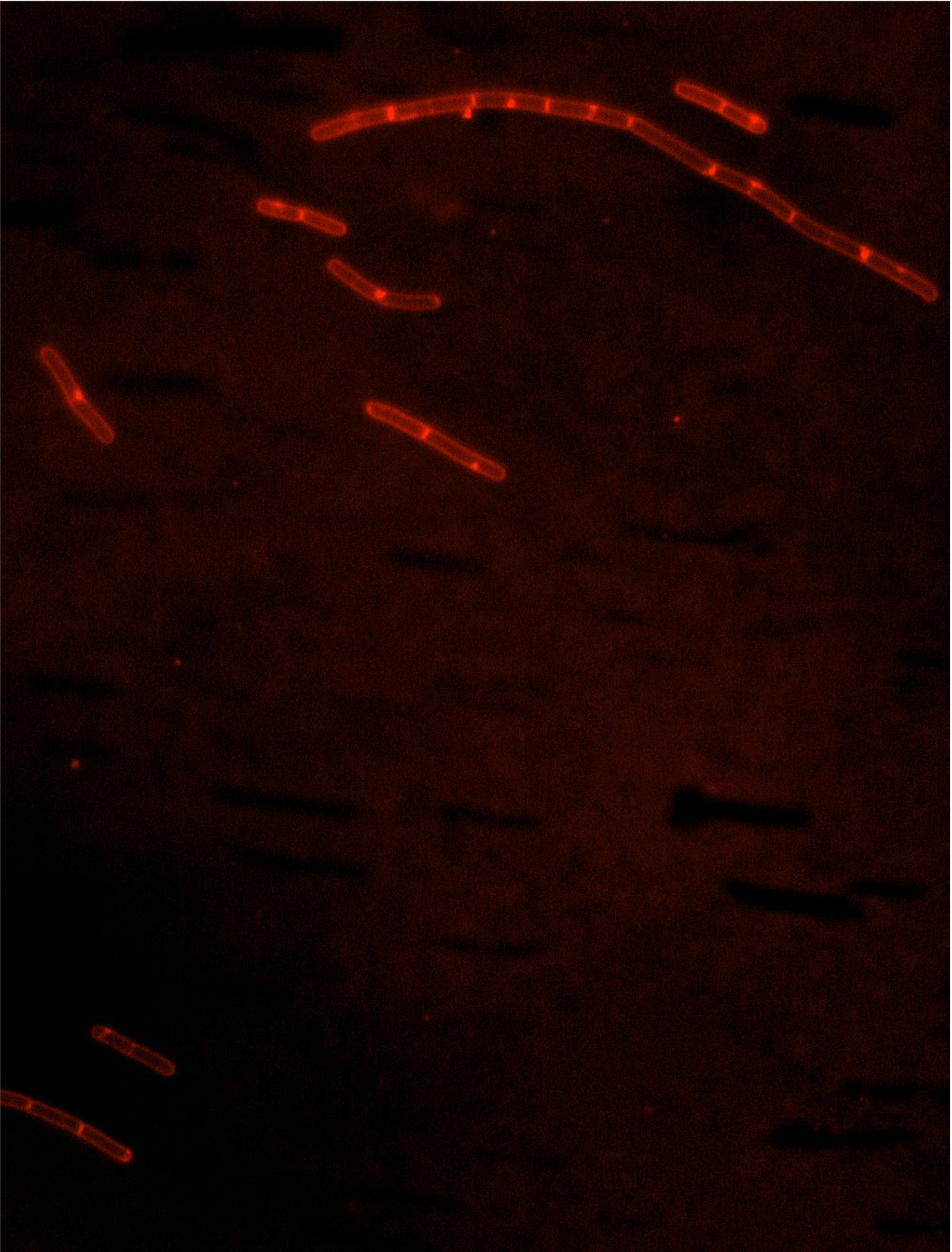

Supplement: Supplementary file 11 — Source data Fig. 6 [file 44319_2025_547_MOESM11_ESM.zip › Figure 6/Fig. 6B/B3.tiff]

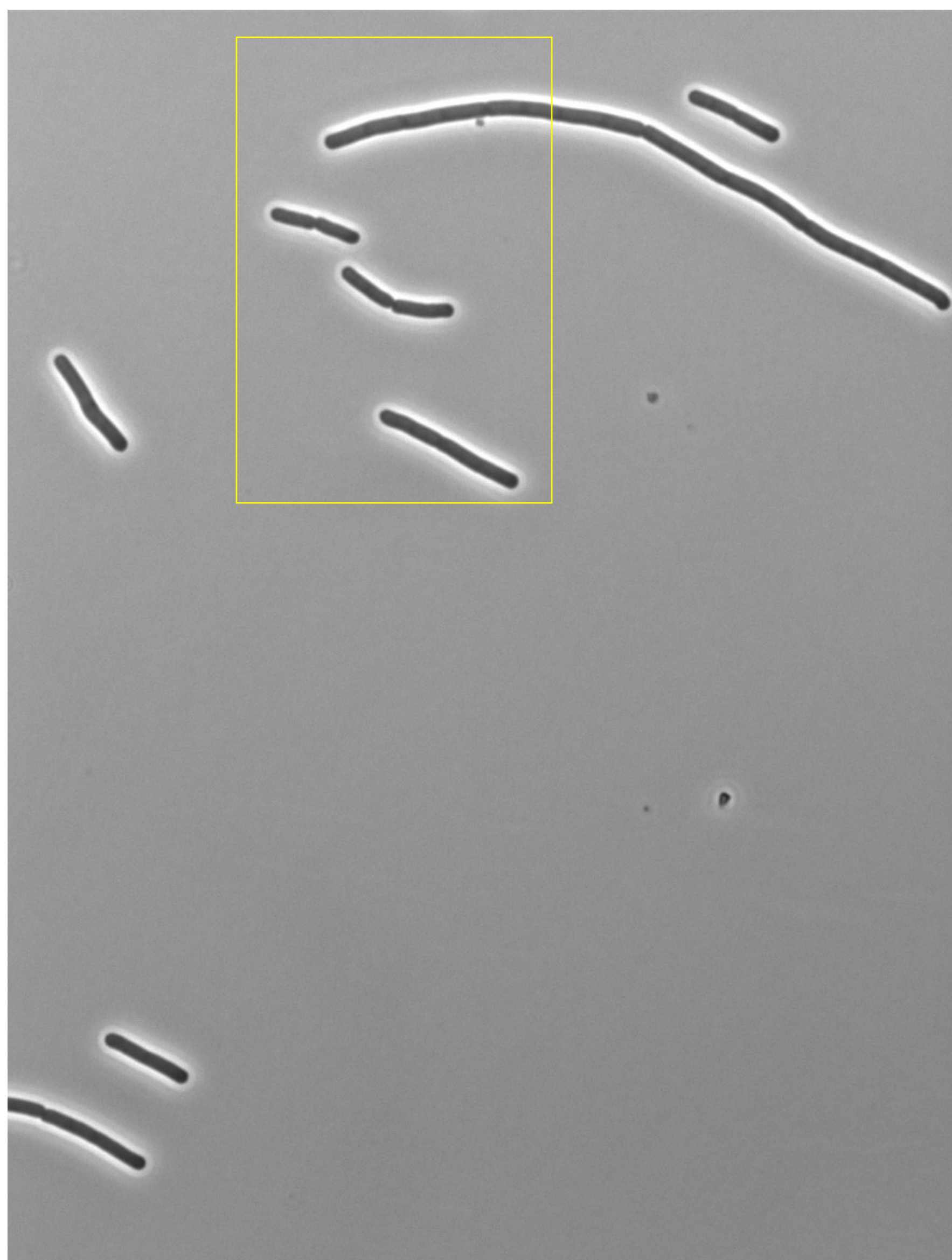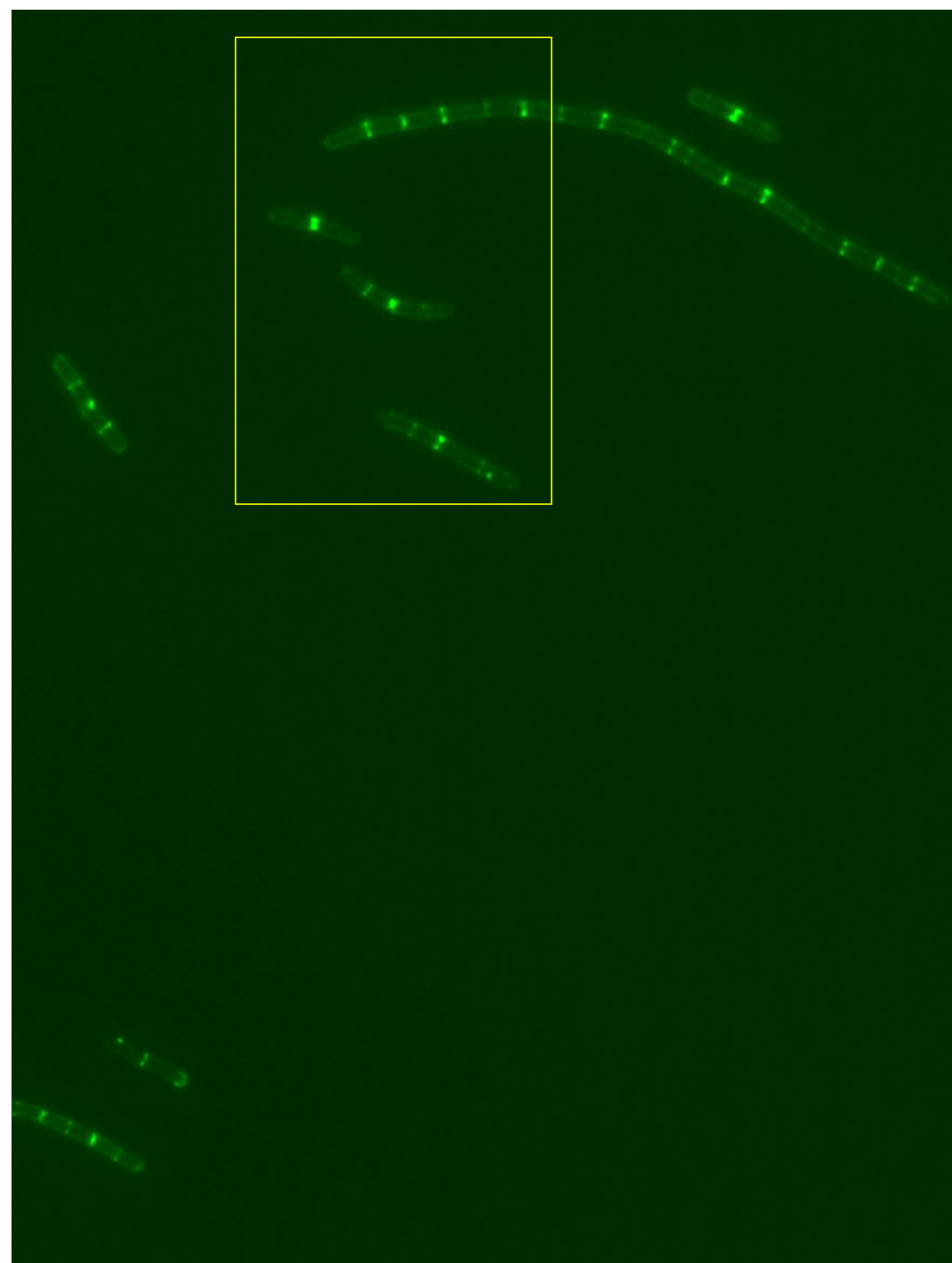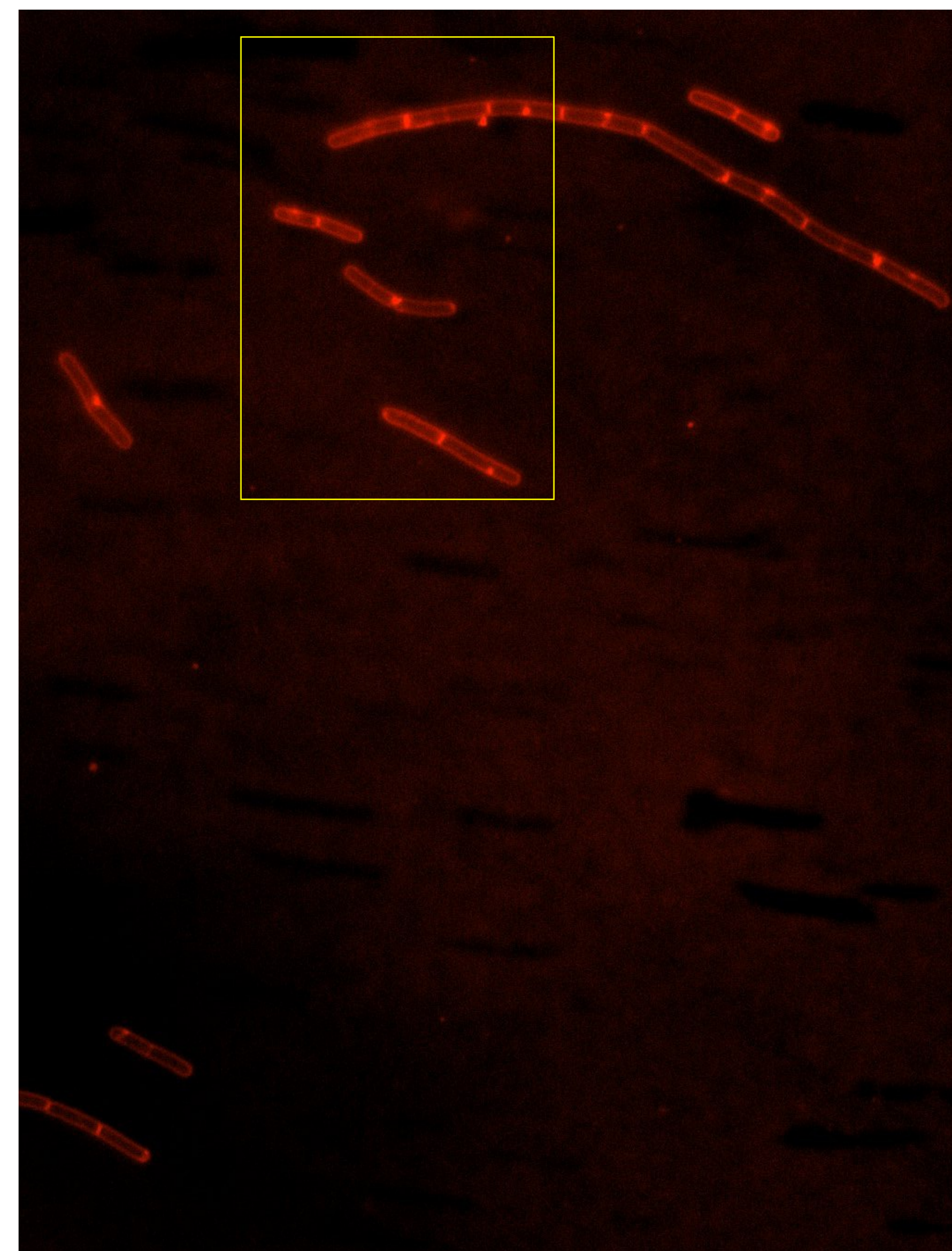

Supplement: Supplementary file 11 — Source data Fig. 6 [file 44319_2025_547_MOESM11_ESM.zip › Figure 6/Fig. 6B/Figure 6B.pdf]

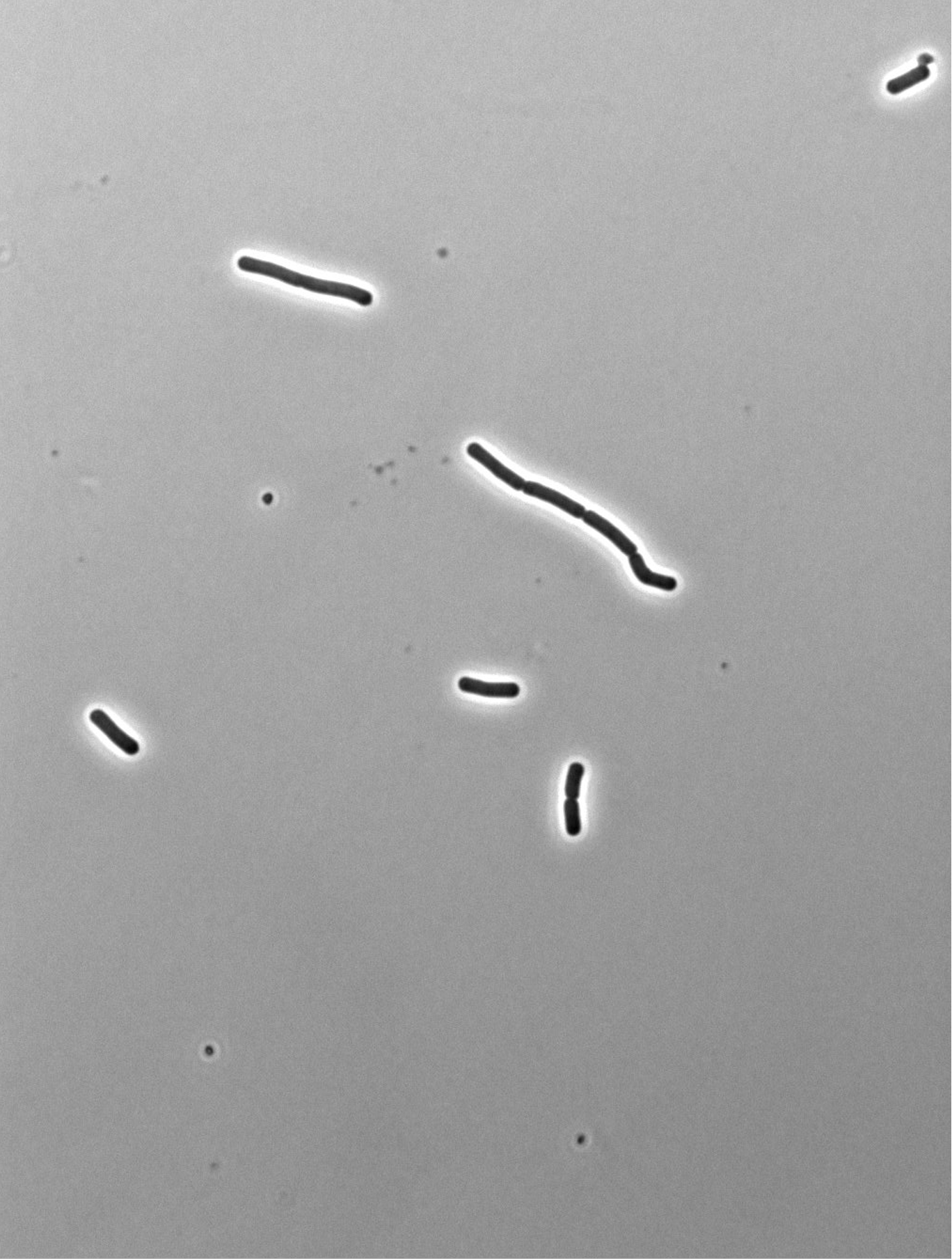

Supplement: Supplementary file 11 — Source data Fig. 6 [file 44319_2025_547_MOESM11_ESM.zip › Figure 6/Fig. 6C/C1.tiff]

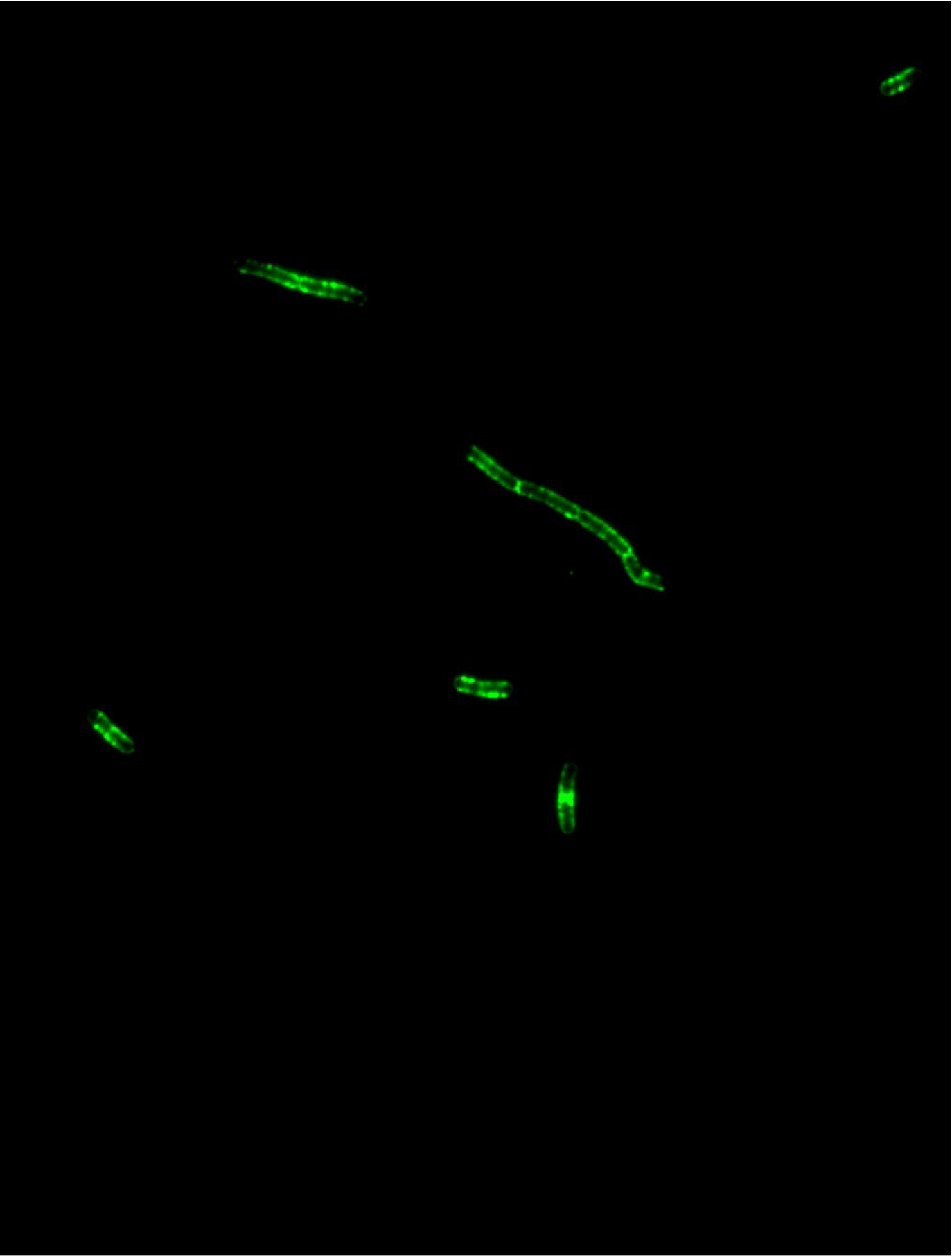

Supplement: Supplementary file 11 — Source data Fig. 6 [file 44319_2025_547_MOESM11_ESM.zip › Figure 6/Fig. 6C/C2.tiff]

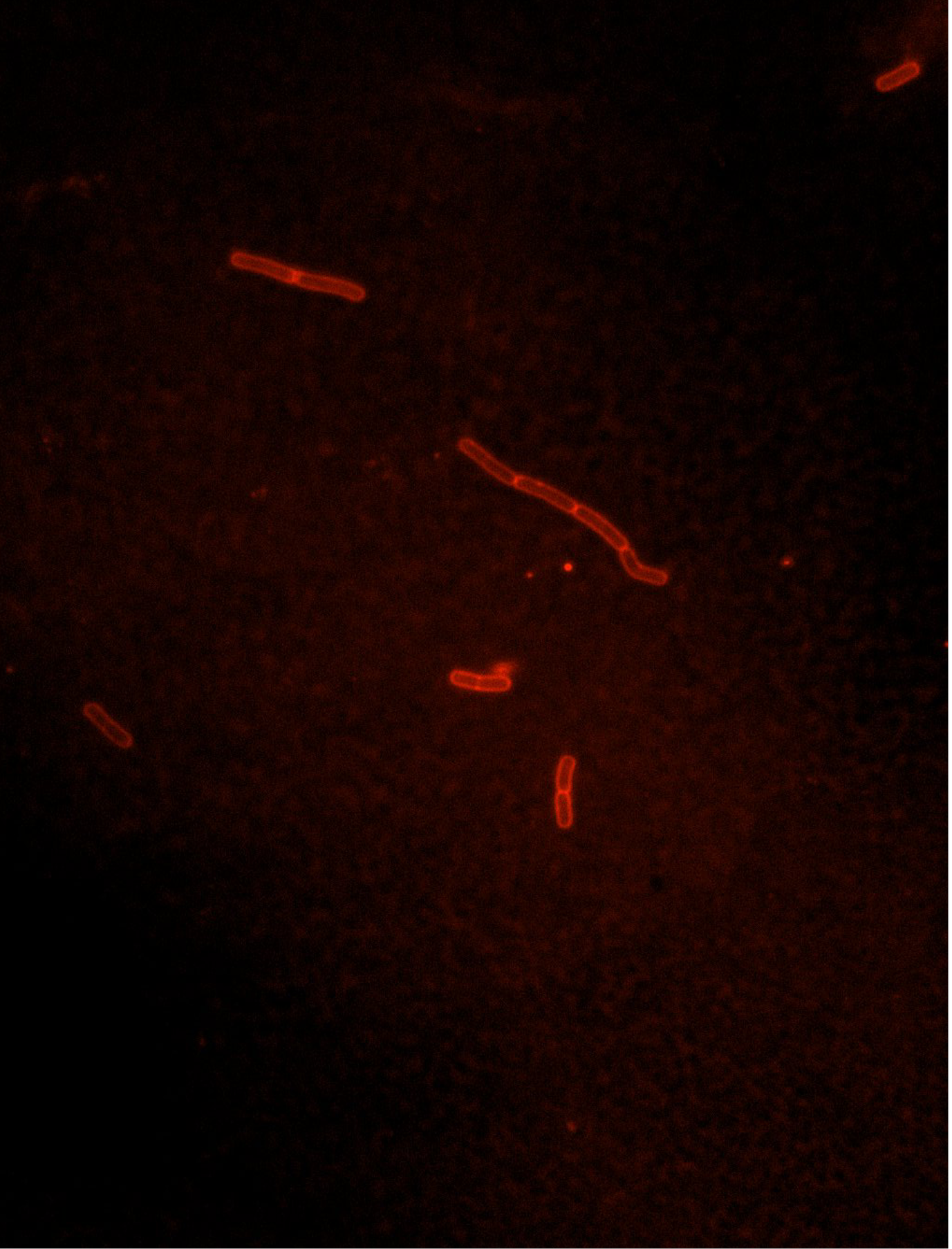

Supplement: Supplementary file 11 — Source data Fig. 6 [file 44319_2025_547_MOESM11_ESM.zip › Figure 6/Fig. 6C/C3.tiff]

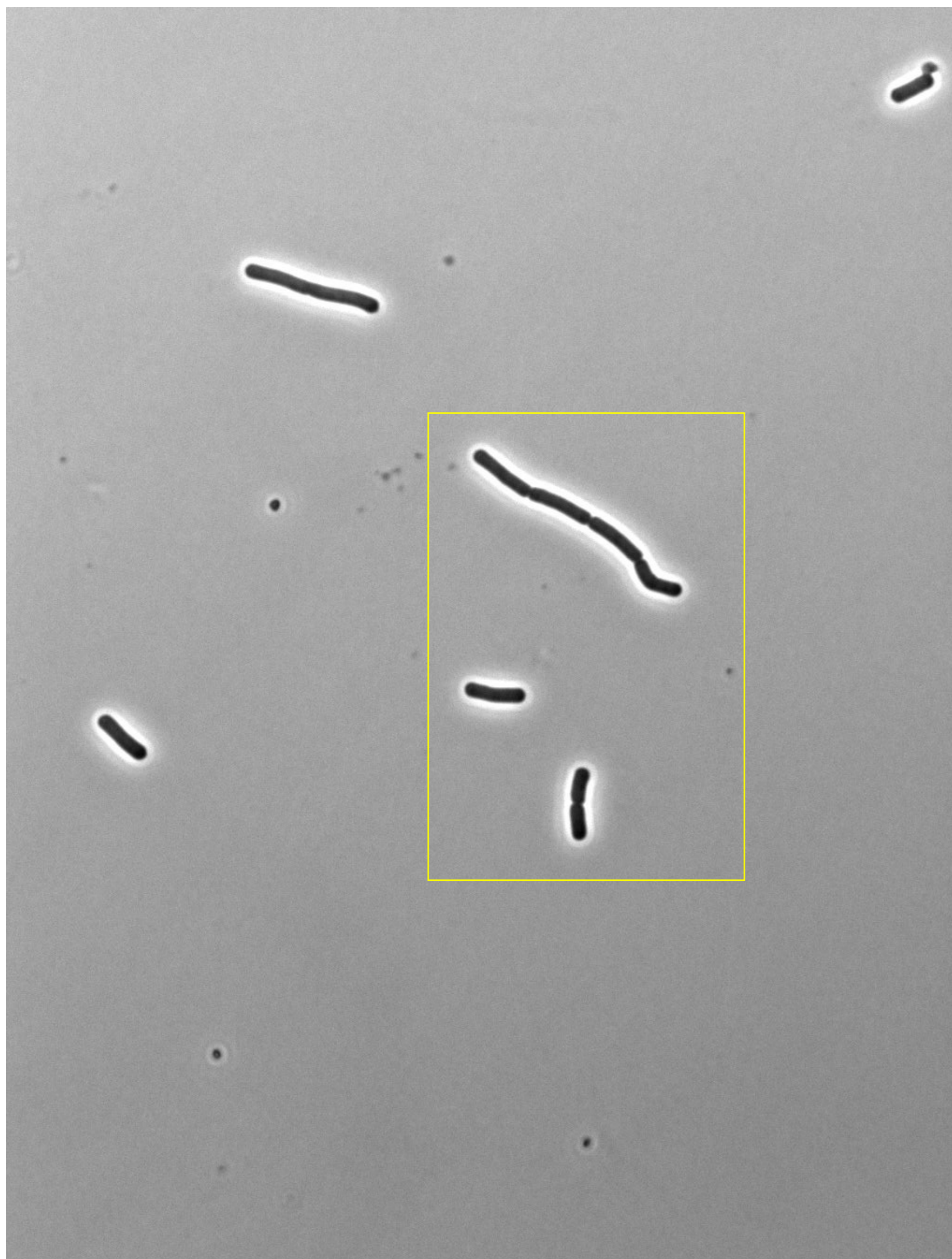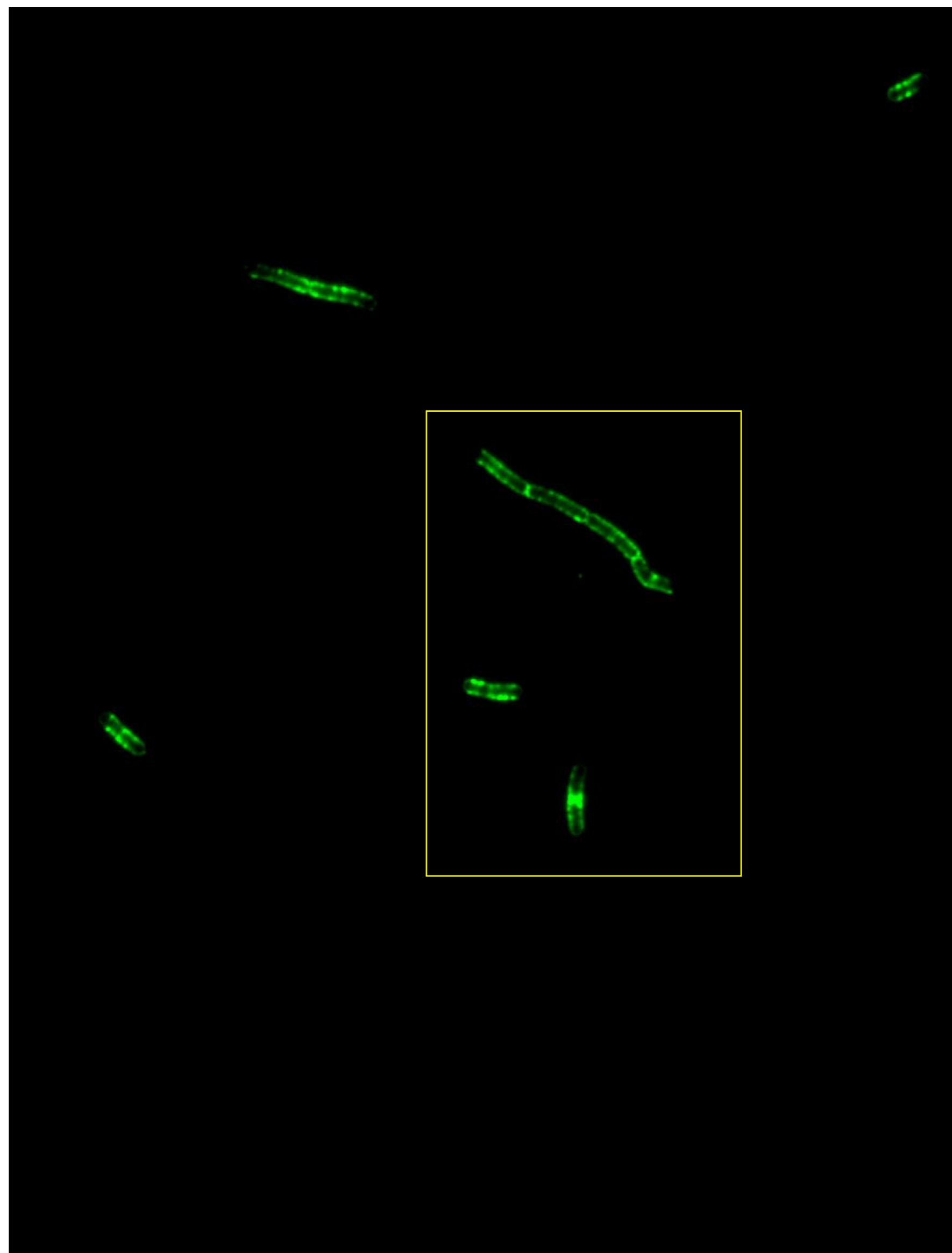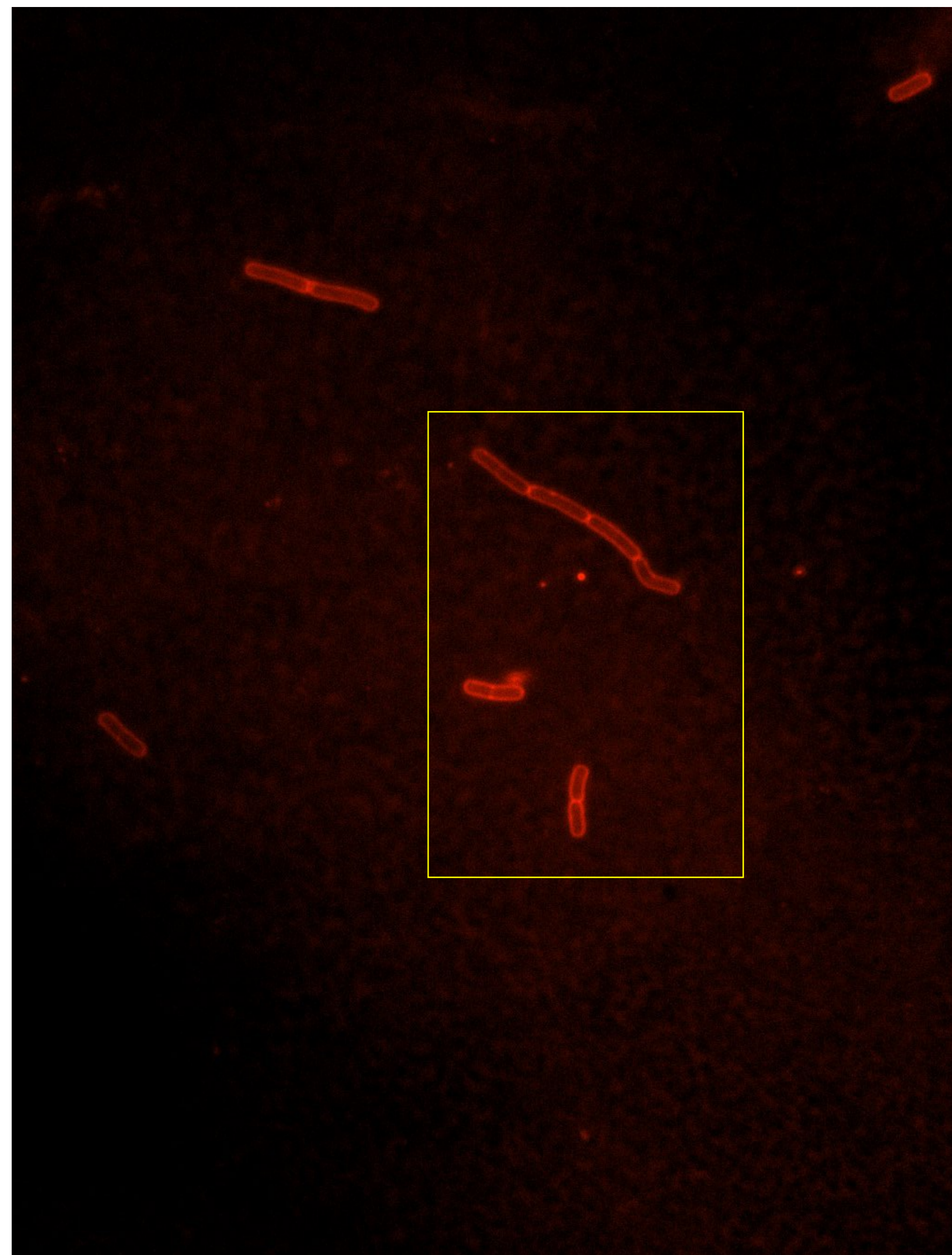

Supplement: Supplementary file 11 — Source data Fig. 6 [file 44319_2025_547_MOESM11_ESM.zip › Figure 6/Fig. 6C/Figure 6C.pdf]

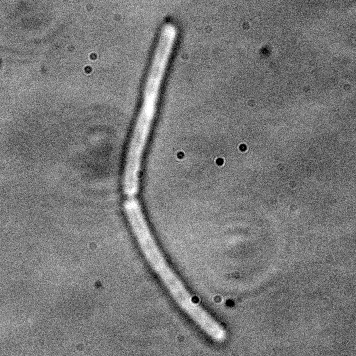

Supplement: Supplementary file 12 — Source data Fig. 7 [file 44319_2025_547_MOESM12_ESM.zip › Figure 7/Fig. 7B.tif]

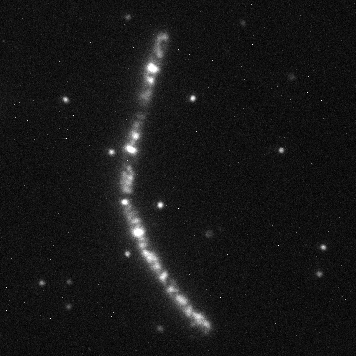

Supplement: Supplementary file 12 — Source data Fig. 7 [file 44319_2025_547_MOESM12_ESM.zip › Figure 7/Fig. 7C.tif]
